# What drives academic peer effects in middle school classrooms in China: Peer composition or peer performance?

**DOI:** 10.1016/j.heliyon.2023.e16840

**Published:** 2023-06-01

**Authors:** Qihui Chen, Chunchen Pei, Yuhe Guo, Shengying Zhai

**Affiliations:** a Beijing Food Safety Policy & Strategy Research Base & College of Economics and Management, China Agricultural University, Beijing, China; b Research Center for Future Education, School of Economics and Resource Management, Beijing Normal University, Beijing, 100875, China; c Agricultural Information Institute, Chinese Academy of Agricultural Sciences, Beijing, China; d College of Economics and Management, China Agricultural University, Beijing, China

**Keywords:** Peer effects, Random class assignment, Quasi-experimental design, Middle school, China

## Abstract

This quasi-experimental study estimates academic peer effects in China's middle school (7th–9th grade) classrooms, using data from a large-scale nationally representative survey of middle schoolers in China. Our study design circumvents endogenous sorting by focusing on 52 schools that randomly assigned incoming 7th graders to different 7th-grade classes. Further, reverse causality is addressed by regressing students' 8th-grade test scores on their (randomly assigned) classmates' average 7th-grade test scores. Our analysis reals that all else equal, a one-standard-deviation increase in (8th-grade) classmates' average 7th-grade test scores raises an individual student's 8th-grade mathematics and English test scores, respectively, by 0.13–0.18 and 0.11–0.17 standard deviations. These estimates remain stable when peer characteristics examined in related peer-effect studies are included in the model. Further analysis reveals that peer effects work through raising individual students' time spent studying per week and their confidence in learning. Finally, classroom peer effects are found to be heterogeneous across subgroups: larger for boys, academically stronger students, students attending better schools (i.e., schools with smaller classes and urban schools), and students with relatively disadvantaged family backgrounds (e.g., lower levels of parental education and family wealth).

## 1 Introduction

Peers', especially classmates' personal characteristics, cognitive ability, and academic performance are widely believed to be key determinants of individual children's schooling attainments, such as school enrollment, test scores, and degree completion [[1], [2], [3], [4], [5], [6], [7]]. Concrete evidence of the direction and scale of classroom peer effects can thus inform educational policy regarding ability tracking, teacher assignment, and class management, such that available (but usually limited) resources can be better utilized to foster school-age children's educational development.

However, despite the considerable effort devoted to estimating academic peer effects in the classroom, existing findings have remained inconclusive. While some studies found strong positive impacts of classmates' academic performance on individual students' performance [[3], [4], [5], [6]], others found moderate to insignificant peer effects in the classroom [8,9]. This inconclusive picture is perhaps not surprising since the driving force of peer effects might vary greatly across study populations and contexts. Differences in students' cognitive ability, family backgrounds, and school quality may all be reasons for the varying magnitudes of existing estimates of peer effects. The discrepancy in findings may also lie in the difficulty of obtaining *causal* estimates of peer effects rooted in the three problems that plague the peer-effect literature [[10], [11], [12]]: 1) endogenous sorting—students form peer groups with others with similar characteristics; 2) reverse causality—individual students' outcomes are affected by their peers' outcome; 3) contextual confounding—there exist common unobserved factors that affect both individual students' and their peers’ educational outcomes.

Owing to the recent implementation of a large-scale, nationally representative survey of middle schoolers (7th–9th graders) in China, namely, the China Education Panel Study (CEPS), researchers have made significant progress in understanding peer effects in China's middle-school classrooms. A particularly useful feature of the CEPS for studying classroom peer effects is that nearly half of the 112 project schools assigned newly-enrolled 7th-graders *randomly* to different 7th-grade classes, helping researchers circumvent potential sorting issues (e.g., students' “self-selection” into different classes based on unobserved characteristics) in their analyses. Taking advantage of random student/class/assignments in this subset of CEPS schools, recent studies have managed to identify the impacts of many types of peer composition and/or peer characteristics on individual students' school performance.^1^ The types of peer characteristics/composition examined include the proportions of female students [13,14], migrant children [15,16], left-behind children [17], only-child classmates [18], classmates with alcoholic fathers [19], and (primary school) repeaters in the class [20,21].

As illuminating as they are, these studies have left an important question unanswered: Do the above-mentioned peer characteristics affect individual students' academic performance *through peers' academic performance* or other channels? Since the CEPS-based studies cited above all focused on estimating reduced-form specifications applied to the first wave of the CEPS data [[13], [20], [14], [15], [16], [17], [18], [19], [21]], none of these studies has directly estimated the effects of classmates' academic performance. Understandably, a cross-section of data lacks the power to address the potential reverse causality running from individual students' own performance to their peers’ performance. Although some CEPS-based studies provided suggestive evidence that the peer characteristics they examined work through changing the learning environment in the classroom [14,19,21], none has directly examined the possibility that peers' academic performance may serve as a working channel.

The current study exploits the panel/longitudinal structure of the CEPS data to circumvent the reverse-causality problem, thereby providing more direct evidence of whether peers' academic performance plays a role in driving classroom peer effects. Our analysis involves several steps. We first estimated the impacts of (randomly assigned) classmates' *7*th*-grade* performance on individual students' performance in 8th grade. Next, we assessed how adding the peer characteristics examined in previous CEPS-based studies to our model may affect our peer-effect estimates, in particular, whether the estimated impacts of classmates’ academic performance were driven by the influence of these characteristics. Finally, we explored several channels that have not been examined in previous CEPS-based studies.

Our analysis, involving 4018 8th-graders in the 52 CEPS schools that reported randomly assigning income 7th graders into 7th-grade classes, yields three important findings. First, there are statistically significant and positive (albeit practically modest) classroom peer effects on individual students' test scores in 8th-grade mathematics and English; peer effects on their Chinese scores are also positive but statistically and quantitively insignificant. All else equal, an increase in 8th-grade classmates' 7th-grade mathematics and English scores of one standard deviation (SD) raise one's 8th-grade mathematics and English score by 0.13–0.18 and 0.11–0.17 SDs, respectively, implying a social multiplier effect of about 1.20 for both mathematics and English learning. These estimates remain robust when previously examined peer characteristics are added to the model. Second, these peer effects work by urging students to spend more time studying and enhancing their confidence in learning. Finally, the effects of classmates' academic performance vary across subgroups: they are larger for boys, academically stronger students, students attending better schools (i.e., schools with smaller classes and urban schools), and students with relatively disadvantaged family backgrounds (i.e., with lower levels of parental education and family wealth).

These analyses help make three contributions to the peer-effect literature. Firstly, our study complements previous CEPS-based studies by identifying the effect of classmates' academic performance, an important form of peer effect that has been overlooked by previous CEPS-based studies. The identified peer effects also allow us to compute the associated “social multiplier” effect of peer performance, which is, no doubt, of policy relevance. Secondly, we explored several working channels of these effects, deepening our understanding of why classmates’ academic performance matters in China's middle school classrooms. Finally, compared with most previous non-CEPS-based studies on academic peer effects in China, which usually employed data from a single province or several counties [3,8,22], our findings based on a nationally representative dataset have much stronger external validity.

## 2 Materials and methods

### 2.1 Ethics statement

The current study was conducted in accordance with the Declaration of Helsinki, and approved by the Human Research Ethics Committee of China Agricultural University (protocol code/approval number: CAUHR-2020-05).

### 2.2 Data source

The analysis performed in this study makes use of two waves of the CEPS data. The CEPS (China Education Panel Study) is a large-scale, nationally representative, school-based survey of middle-school students (7th–9th graders) in China. The CEPS project was designed and conducted by the China Data and Survey Center of the Renmin University of China, and was reviewed and approved by the Institutional Review Board of the Remin University. In the survey, written informed consent to participate project was provided by the participant's legal guardian/next of kin. The data used in this study are publicly available, second-hand data, which do not include any private information of the study subjects and are not individually identifiable.

In the 2013-14 academic year (the baseline), the CEPS adopted a multi-stage, stratified PPS (Probability-Proportional-to-Size) strategy to sample and select participating students. Several steps were involved. First, 28 urban districts or rural counties were selected using the average schooling level of the local population and the proportion of migrants in the local population as stratifiers. Next, four middle schools from each of the 28 chosen districts or counties were selected, using school type (public schools, private schools, etc.) and enrollment size as stratifiers. A total of 112 schools were thus sampled. In each sampled school, two 7th-grade and two 9th-grade classes were randomly chosen, yielding a total of 438 sampled classes.^2^ All (19,487) students enrolled in these 438 sampled classes in the 2013-14 academic year (and their parents or legal guardians) participated in the CEPS project. Four waves of follow-up surveys took place in 2014–2018, but currently, only the first two waves of data (collected in the 2013–14 and 2014-15 academic years) are made publicly available. Thus, we base our empirical analysis on the two publicly available waves of the CEPS data. Moreover, we restrict our analysis to those 7th graders interviewed at the baseline (*N* = 10,279, 92% of whom participated in the second wave) as most of the baseline 9th graders had graduated by the second wave and could not be tracked.

**Fig. 1:**
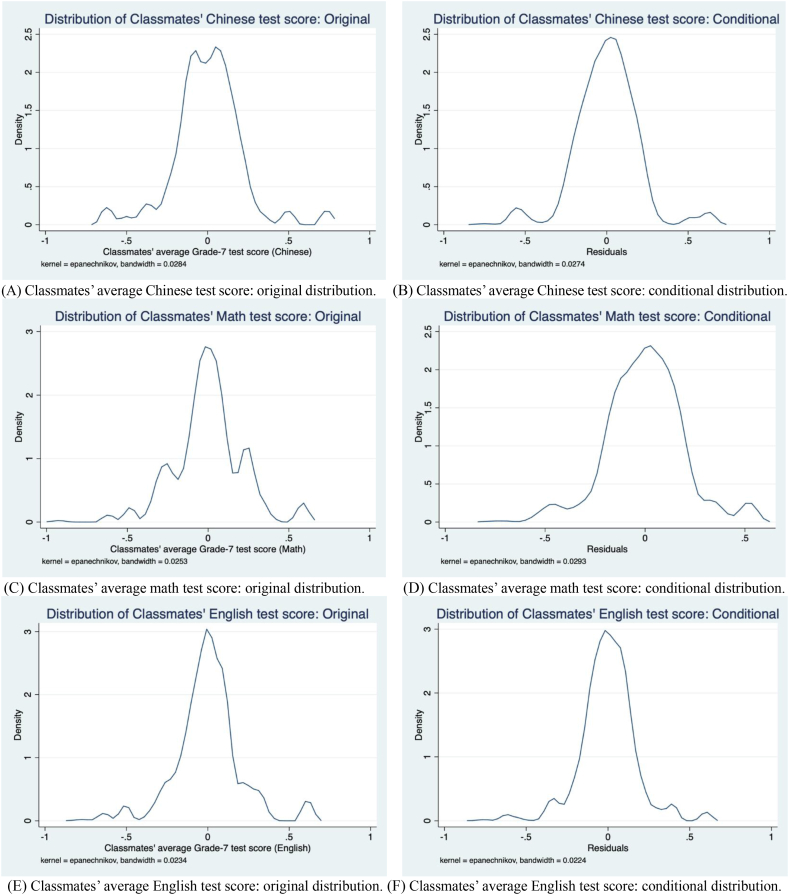
Distributions of classmates' average 7th-grade test scores. (A) Classmates' average Chinese test score: original distribution. (B) Classmates' average Chinese test score: conditional distribution. (C) Classmates' average mathematics test score: original distribution. (D) Classmates' average mathematics test score: conditional distribution. (E) Classmates' average English test score: original distribution. (F) Classmates' average English test score: conditional distribution. Notes: The conditional distribution is the distribution of residuals obtained from regressing classmates' average test scores on the full set of controls (reported in Table 1) and school FEs. The corresponding (1-R^2^) measures from the regression are, respectively, 0.8327, 0.8280, and 0.7129 in panel (B), (D), and (F).

During each wave of the survey, information on sampled students' educational development was collected through interviews using questionnaires separately administered to sampled students themselves, their parents (or legal guardians), and their teachers (including subject teachers, grade headteachers, and school principals). The most important variables in this study are students' academic performance in Chinese, mathematics, and English, the three most important subjects in China's middle school curriculum. Presumably, due to logistical considerations, the CEPS collected only scores on the midterm exam of the first (Fall) semester in each grade. Because the tests were designed by the project schools (rather than the CEPS team), the specific contents of them differ somewhat across schools. As such, to facilitate comparison and interpretation, test scores were standardized *within schools* (with a mean of zero and a SD of one). Fig. 1, panels A–F, plotting both the original and conditional distributions of 8th-grade classmates' average 7th-grade test scores for our analytical sample (—see the next subsection for sample construction), suggests that these peer-performance measures have sufficient variations for identification purposes.

Also collected were data on sampled students’ individual characteristics (gender, age, cognitive skills, etc.), household characteristics (sibship size, parental education, parental employment, family income, etc.), teacher characteristics (gender, years of schooling, teaching experience, etc.), and class/school characteristics (class size, conditions of school facilities, etc.).

### 2.3 Sample characteristics

To implement our quasi-experimental design, we followed previous CEPS-based studies [13,23] and imposed several sample restrictions to construct our study sample. First, we began with the 93 CEPS project schools (of a total of 112) whose principals reported randomly assigning incoming 7th graders to different 7th-grade classes. Next, 34 sampled schools were excluded, where 7th-grade headteachers did *not* uniformly confirm that class assignments in 7th grade were *not* based on students’ (previous) test scores.^3^ Finally, for reasons that will become clear in Section 2.5, seven schools that reassigned their students to different classes in 8th grade were further excluded. Applying these restrictions yielded a final analytical sample of 52 focal schools and 4018 students attending these schools. These students were attending 7th grade at the baseline and 8th grade at the time of the second wave.

Table 1 depicts the sample profile of these students. Slightly more than half (51%) of the 4018 sampled students are boys. On average, the students were 14 years old in 2015, having 0.6 siblings. Their fathers and mothers had completed, on average, 11.1 and 10.5 years of formal schooling, respectively. The average class had an enrollment size of 49, taught by a Chinese/mathematics/English teacher with 15.6/15.6/15.5 years of education and 15.2/17.1/16.6 years of teaching experience. These figures are very close to official statistics for China as a whole [24], verifying the representativeness of our analytical sample.^4^

**Table 1:** Summary statistics.

Variables	Mean (proportion)	SD	Variables	Mean (proportion)	SD
*A. Outcome variables (8*th *grade)*			*E. Explanatory/control variables (8*th *grade)*		
Chinese test scores (standardized)	0.032	0.969	*Personal characteristics*		
Mathematics test scores (standardized)	0.033	0.983	Age (months)	166.46	7.622
English test scores (standardized)	0.031	0.980	Boy (dummy, = 1 if yes)	0.510	0.500
Hours spent doing homework on weekdays	2.138	1.089	Cognitive ability test score (standardized)	0.399	0.832
Hours spent doing homework on weekends	1.575	0.989	Birth order	1.270	0.569
Perceived difficulty in learning (dummies):			*Family-level characteristics*		
Not feeling difficulty in learning Chinese in 8th grade	0.723		Number of siblings	0.552	0.770
Not feeling difficulty in learning Mathematics in 8th grade	0.498		Father's education (years)	11.084	3.329
Not feeling difficulty in learning English in 8th grade	0.517		Mother's education (years)	10.529	3.506
Not feeling difficulty in learning Chinese in 6th grade	0.808		Rural residential permit (dummy, = 1 if yes)	0.422	0.494
Not feeling difficulty in learning Mathematics in 6th grade	0.677		Household income (dummies):		
Not feeling difficulty in learning English in 6th grade	0.673		Very poor	0.026	
*B. Test scores (7*th *grade)*			Poor	0.117	
			Average	0.767	
Chinese test scores (standardized)	0.024	0.981	Rich	0.084	
Mathematics test scores (standardized)	0.021	0.985	Very rich	0.006	
English test scores (standardized)	0.027	0.974	*School-level characteristics*		
Classmates' average 7th-grade test score (Chinese)	0.001	0.209	Class size	49.058	15.816
Classmates' average 7th-grade test score (mathematics)	0.000	0.211	Headteacher is male (dummy, = 1 if yes)	0.308	0.462
Classmates' average 7th-grade test score (English)	0.000	0.199	Headteacher's education (years)	15.618	0.792
*C. Peer family-background characteristics (7*th *grade)*			Headteacher's teaching experience (years)	15.990	8.107
			Chinese teacher is headteacher (dummy, = 1 if yes)	0.239	0.426
Classmates' average maternal education (years)	11.065	1.956	Chinese teacher is male (dummy, = 1 if yes)	0.179	0.383
Classmates' average paternal education (years)	10.511	2.123	Chinese teacher's education (years)	15.643	0.834
Classmates' household income: “rich” & “very rich” (%)	7.843		Chinese teacher's teaching experience (years)	15.247	7.855
*D. Pre-determined peer characteristics (7*th *grade)*			Mathematics teacher is headteacher (dummy, = 1 if yes)	0.315	0.465
			Mathematics teacher is male (dummy, = 1 if yes)	0.390	0.488
Proportion of girl peers	0.483	0.074	Mathematics teacher's education (years)	15.582	0.577
Proportion of migrant peers	0.248	0.215	Mathematics teacher's teaching experience (years)	17.067	9.082
Proportion of low-ability peers	0.097	0.141	English teacher is headteacher (yes = 1)	0.281	0.450
Proportion of disruptive peers	0.071	0.053	English teacher is male (dummy, = 1 if yes)	0.105	0.307
Proportion of only-child peers	0.551	0.241	English teacher's education (years)	15.534	0.744
			English teacher's teaching experience (years)	16.602	8.304

Notes: Number of observations (*N*) = 4018. The sample includes 52 schools that randomly assigned newly-enrolled students to 7th-grade classes but did not reassign students in 8th grade. The sample includes only students who were attending 7th grade at the baseline (i.e., the 2013–2014 academic year).

### 2.4 Random student/class assignments in 7th grade in focal schools: evidence

To check whether class assignments in 7th grade were indeed performed randomly in the 52 focal schools, we examined the correlations between individual students' own and classmates' 7th-grade test scores in these schools (—as noted in Section 2.2, these are midterm exam scores of the Fall semester in 7th grade). Table 2, panel A, reports the results for the three core subjects: columns (1)–(2) for Chinese test scores, columns (3)–(4) for mathematics test scores, and columns (5)–(6) for English test scores. The results reported in odd-numbered columns suggest that without controlling for any other covariates, the correlations between one's own and classmates' average 7th-grade test scores are large and statistically significant for all three subjects. In contrast, the correlations became much smaller and statistically insignificant once conditional on school fixed effects and students' observed individual and household characteristics reported in Table 1.

**Table 2:** Correlations between one's and classmates' test scores in 7th grade.

	(1)	(2)	(3)	(4)	(5)	(6)
	Chinese		Mathematics		English	
	A. 52 focal schools with random class assignments in 7th grade but did not reassign students in 8th grade					
Classmates' average 7th-grade Chinese test score rowhead	0.454*** (0.123)	0.163 (0.127)				
Classmates' average 7th-grade mathematics test score			0.553*** (0.109)	0.182 (0.118)		
Classmates' average 7th-grade English test score					0.446*** (0.141)	0.120 (0.147)
Personal characteristics	No	Yes	No	Yes	No	Yes
Family characteristics	No	Yes	No	Yes	No	Yes
Teacher characteristics	No	Yes	No	Yes	No	Yes
School fixed effects	No	Yes	No	Yes	No	Yes
N	4018	4018	4018	4018	4018	4018
R2	0.009	0.225	0.015	0.207	0.009	0.224
	B. 60 schools excluded from the final analytical sample					
Classmates' average 7th-grade Chinese test score	0.779*** (0.041)	0.488*** (0.048)				
Classmates' average 7th-grade mathematics test score			0.768*** (0.046)	0.405*** (0.060)		
Classmates' average 7th-grade English test score					0.823*** (0.036)	0.564*** (0.036)
Personal characteristics	No	Yes	No	Yes	No	Yes
Family characteristics	No	Yes	No	Yes	No	Yes
Teacher characteristics	No	Yes	No	Yes	No	Yes
School fixed effects	No	Yes	No	Yes	No	Yes
N	5099	5099	5099	5099	5099	5099
R2	0.054	0.263	0.046	0.234	0.075	0.276

Notes: Standard errors in parentheses, clustered at the class level. ***p < 0.01.

A further “falsification” test is to estimate the correlations between individual students' 7th-grade test scores and their classmates' average 7th-grade test scores in the 60 CEPS project schools that were excluded from our final analytical sample (—recall section 2.3), which potentially assigned their newly-enrolled 7th graders into 7th-grade classes in a non-random manner. The results reported in Table 2, panel B, indicate that in these 60 non-focal schools, the correlations between individual students' 7th-grade test scores and their classmates’ average 7th-grade test scores remained large and statistically significant, even after school fixed effects and observed individual and household characteristics were included in the models. The contrast in the patterns observed in these two sets of schools provides further support to the presumption that class assignments in 7th grade were indeed done randomly in the 52 focal schools.

A third way to test the presumption of random student/class assignments is to test whether the distribution of educational resources was indeed “balanced” across different 7th-grade classes within given focal schools. More specifically, we followed recent CEPS-based studies and tested if the means of individual students' observed personal and family characteristics (e.g., age, gender, cognitive ability test scores that measure one's general logical thinking and analytical skills, birth order, sibship size, parental education, household registration status, and household income) within a class are correlated with class characteristics (e.g., class size, teacher education, teaching experiences, teaching load, and teaching style) [16,17]. Table 3 reports the results of the test: conditional on the school an individual student was attending, virtually all the correlations are statistically insignificant. Only two correlations (i.e., those between students' average age and their teachers' time spent grading homework and between students' household income level and teachers’ time spent on lesson planning) are statistically significant. Yet given the large number of correlations being examined in Table 3, these few significant ones are likely driven by sampling variations.

**Table 3:** Balancing test for the allocation of educational resources across classes within schools.

	(1)	(2)	(3)	(4)	(5)	(6)	(7)	(8)	(9)
	Average student age	Proportion of boys	Average cognitive ability test score	Average birth order	Average number of siblings	Average years of paternal education	Average years of maternal education	Prop. Of classmates with rural “Hukou”	Prop. Of classmates with “above average” household income
Class size	0.078 (0.068)	−0.001 (0.001)	−0.010 (0.019)	−0.007 (0.005)	−0.012 (0.008)	−0.008 (0.022)	−0.006 (0.028)	−0.003 (0.005)	−0.005 (0.005)
Teacher's: Age	−0.025 (0.055)	0.003 (0.002)	0.007 (0.011)	−0.003 (0.005)	−0.003 (0.007)	−0.005 (0.023)	−0.010 (0.027)	−0.003 (0.005)	−0.003 (0.002)
Gender	0.132 (0.514)	−0.005 (0.017)	−0.077 (0.130)	0.038 (0.036)	0.047 (0.061)	−0.274 (0.176)	0.009 (0.218)	0.018 (0.033)	−0.019 (0.019)
Education	−0.229 (0.258)	−0.004 (0.006)	−0.013 (0.053)	0.001 (0.018)	0.017 (0.026)	−0.035 (0.101)	0.114 (0.124)	0.005 (0.014)	−0.003 (0.010)
Teaching experience	0.048 (0.038)	−0.001 (0.001)	0.001 (0.007)	0.002 (0.003)	0.001 (0.005)	−0.006 (0.014)	0.003 (0.016)	0.002 (0.003)	0.002 (0.001)
Total number of classes taught	0.124 (0.218)	−0.003 (0.005)	−0.020 (0.033)	0.005 (0.014)	−0.001 (0.019)	−0.108 (0.091)	−0.155 (0.102)	−0.011 (0.021)	0.005 (0.005)
Weekly hours spent teaching	0.028 (0.054)	−0.004 (0.003)	0.015 (0.016)	0.003 (0.005)	0.006 (0.007)	−0.034 (0.031)	−0.030 (0.033)	0.004 (0.004)	0.003 (0.005)
Weekly hours spent planning lessons	0.031 (0.053)	0.001 (0.001)	0.000 (0.009)	−0.001 (0.004)	−0.003 (0.004)	0.008 (0.014)	−0.014 (0.020)	0.004 (0.003)	0.005** (0.002)
Weekly hours spent grading homework	−0.072*** (0.020)	−0.000 (0.001)	−0.002 (0.006)	0.003 (0.002)	0.005 (0.003)	0.002 (0.011)	0.009 (0.014)	0.001 (0.002)	−0.001 (0.001)
Graduated from teacher-training schools/majors	0.381 (0.669)	−0.022 (0.024)	0.020 (0.165)	−0.038 (0.065)	−0.011 (0.087)	−0.232 (0.320)	−0.206 (0.470)	−0.015 (0.041)	0.022 (0.036)
Received awards	−0.046 (0.207)	0.011 (0.009)	−0.034 (0.049)	−0.013 (0.016)	0.015 (0.020)	0.122 (0.079)	0.061 (0.111)	−0.008 (0.011)	−0.013 (0.008)
Backbone teacher	−0.072 (0.410)	0.008 (0.014)	−0.005 (0.113)	−0.009 (0.040)	−0.084 (0.057)	−0.167 (0.186)	0.159 (0.225)	−0.041 (0.039)	−0.009 (0.022)
School fixed effects	Yes	Yes	Yes	Yes	Yes	Yes	Yes	Yes	Yes
Constant	154.700*** (4.359)	0.601*** (0.145)	0.487 (1.042)	1.951*** (0.442)	1.241** (0.608)	12.826*** (1.765)	9.903*** (2.173)	0.556 (0.335)	1.125*** (0.288)
*N*	98	98	98	98	98	98	98	98	98
R^2^	0.971	0.913	0.840	0.906	0.940	0.971	0.967	0.958	0.916

Notes: Analyses reported in this table are performed at the class level. Standard errors in parentheses. **p < 0.05, ***p < 0.01.

The tests discussed in this subsection lend strong support to the presumption that in the 52 focal schools, incoming 7th-graders were assigned to 7th-grade classes in a random manner *within schools*, which offers a unique opportunity to identify within-class academic peer effects in these schools.

### 2.5 Estimation framework

To develop an empirical framework for estimating the effects of classmates’ academic performance, we begin with the standard “linear-in-mean” specification [11].

(1) $$y_{ics}=\beta_{0}+\beta_{1}{\bar{y}}_{-i,cs}+\beta_{2}Z_{ics}+u_{ics}$$

In Equation (1), the outcome variable $y_{ics}$ stands for a test score in a core subject (Chinese, mathematics, or English) for student *i* in class *c* of school *s*; ${\bar{y}}_{-i,cs}$ represents the “leave-me-out” mean of the corresponding test scores of this student's classmates (who are denoted by “−i”); **Z** is a set of individual, family, class, and school characteristics reported in Table 1; as no set of observed factors can fully explain variations in $y_{ics}$, a disturbance term u is added to balance the two sides of the equation, capturing potential influences of unobserved factors and measurement errors of the observed factors and the outcome variable. If Equation (1) is well-specified, $\beta_{1}$ is the parameter of primary interest, which captures the impact of (randomly assigned) classmates' (average) academic performance on individual students' performance; and the method of OLS (ordinary least-squares) can provide consistent estimates of $\beta_{1}$.

However, OLS estimates of $\beta_{1}$ may be biased due to two potential identification problems. First, since any student is also his/her classmates' classmate, there may exist reverse causality operated from a student *i*'s own academic performance, $y_{ics}$, to *i*'s classmates' (average) performance, ${\bar{y}}_{-i,cs}$. In that case, OLS will overestimate the actual peer effects. Second, there might exist unobserved confounding factors (in u) that affect a student's own and his/her classmates' academic performance simultaneously, thereby creating a spurious correlation between $y_{ics}$ and ${\bar{y}}_{-i,cs}$. In the case of “endogenous sorting”, for instance, where sampled schools assigned incoming 7th-graders to different 7th-grade classes based on their talent unobserved by the researcher, then $y_{ics}$ and ${\bar{y}}_{-i,cs}$ may be correlated even if there is no real causal relationship between them.

As discussed above, the CEPS data provide a unique opportunity to circumvent endogenous sorting. More than half of the CEPS project schools (59 out of 112) reported that they *randomly* assigned incoming 7th graders to different classes upon enrollment (—we have provided evidence for the plausibility of this claim in Section 2.4). Because 7th grade is the first grade in the middle school system in China, such an assignment mechanism created an “experiment” that randomly mixed an incoming 7th-grader with other incoming 7th-graders with different demographic markups, personality traits, and talents. Conditioning on the school that a student is attending, the within-school random classmate assignment also balances out the influence of unobserved confounders. Our analysis, therefore, focuses on the 59 schools with random classmate assignments. Yet, for reasons discussed immediately below, we further excluded 7 schools that reassigned students to different classes in 8th grade.

To circumvent the potential reverse causality from $y_{ics}$ to ${\bar{y}}_{-i,cs}$, we replace ${\bar{y}}_{-i,cs}$ with its (one-year) lagged measure. Specifically, we estimate:

(2) $$y_{ics}^{G8}=\beta_{0}+\beta_{1}{\bar{y}}_{-i,cs}^{G7}+\beta_{2}Z_{ics}+u_{ics}$$

where $y_{ics}^{G8}$ is a student *i*'s 8th-grade test score of a given subject (i.e., the midterm exam score of the Fall semester in 8th grade) and ${\bar{y}}_{-i,cs}^{G7}$ is his/her classmates' average 7th-grade test score in that subject (i.e., the midterm score of the Fall semester in 7th grade). Since ${\bar{y}}_{-i,cs}^{G7}$ was generated before $y_{ics}^{G8}$, the latter cannot affect the former, which eliminates the concern about reverse causality. But in schools that “reshuffled” students in 8th grade, many students' 7th-grade classmates were no longer their 8th-grade classmates; as such, ${\bar{y}}_{-i,cs}^{G7}$ would not really capture one's 8th-grade classmates' 7th-grade performance in these schools. Therefore, we excluded seven schools that “reshuffled” students in 8th grade in the analysis.

A potential concern is that the lagged peer-performance measure available in the CEPS data, ${\bar{y}}_{-i,cs}^{G7}$, was constructed based on classmates' *midterm exam* scores of the Fall semester in 7th grade—i.e., it was measured about two months *after* random class assignments took place in 7th grade. As such, peer interactions during these two months may open some room for individual students to affect their classmates' academic performance, suggesting individual students' performance during this period as a potential omitted channel variable. To address this issue, we include student *i*'s own *midterm exam* scores, $y_{ics}^{G7}$, in the model:

(3) $$y_{ics}^{G8}=\beta_{0}+\beta_{1}{\bar{y}}_{-i,cs}^{G7}+\delta_{1}y_{ics}^{G7}+\beta_{2}Z_{ics}+u_{ics}$$

Since $y_{ics}^{G7}$ was also measured about two months *after* random class assignments took place in 7th grade, the inclusion of it effectively “blocks” the channel through which peer interactions occurring during the first two months of 7th grade enter the model. Put differently, with $y_{ics}^{G7}$ being held fixed, $\beta_{1}$ captures the effect of classmates' academic performance after both ${\bar{y}}_{-i,cs}^{G7}$ and $y_{ics}^{G7}$ were observed.

## 3 Empirical results

### 3.1 Main findings

This subsection reports the main findings of this paper. Table 4 presents estimates of classroom peer effects on individual students' 8th-grade test scores in three core subjects: Chinese (columns 1–3), mathematics (columns 4–6), and English (columns 7–9). Since, as discussed above, random student/class assignments of incoming 7th graders were done *within* the 52 focal schools, all estimates reported in Table 4 have been adjusted for school fixed effects. Three empirical specifications were adopted for each of the three core subjects. The first controls only for students' personal characteristics (e.g., one's own 7th-grade test score, gender, age, and the score of a cognitive ability test that measures one's general logical and analytical skills rather than his/her subject-specific knowledge), in addition to school fixed effects. The second specification adds the household characteristics reported in Table 1, panel E, to the model. The final specification further includes the characteristics of subject teachers reported in Table 1, panel E, as additional controls.

**Table 4:** Effects of classmates' average 7th-grade test scores on individual students’ 8th-grade test scores.

Outcome variable: standardized 8th-grade test scores	(1)	(2)	(3)	(4)	(5)	(6)	(7)	(8)	(9)
	Chinese			Mathematics			English		
Classmates' average 7th-grade Chinese test score	0.044 (0.061)	0.051 (0.061)	0.038 (0.068)						
Classmates' average 7th-grade mathematics test score				0.180*** (0.061)	0.177*** (0.061)	0.134** (0.062)			
Classmates' average 7th-grade English test score							0.169*** (0.042)	0.163*** (0.041)	0.106*** (0.034)
Own 7th-grade test scores (subject-specific)	0.578*** (0.019)	0.576*** (0.019)	0.576*** (0.018)	0.617*** (0.021)	0.614*** (0.021)	0.612*** (0.020)	0.766*** (0.017)	0.764*** (0.017)	0.764*** (0.017)
Age (months)	−0.003 (0.002)	−0.002 (0.002)	−0.002 (0.002)	−0.004** (0.002)	−0.003** (0.002)	−0.003* (0.002)	−0.001 (0.001)	−0.001 (0.001)	−0.000 (0.001)
Boy (dummy, = 1 if yes)	−0.234*** (0.024)	−0.233*** (0.024)	−0.232*** (0.024)	−0.095*** (0.025)	−0.103*** (0.025)	−0.105*** (0.025)	−0.130*** (0.020)	−0.133*** (0.020)	−0.133*** (0.020)
Cognitive ability test score (standardized)	0.273*** (0.023)	0.270*** (0.023)	0.273*** (0.023)	0.303*** (0.020)	0.302*** (0.020)	0.301*** (0.020)	0.138*** (0.017)	0.137*** (0.017)	0.135*** (0.017)
Birth order		−0.011 (0.030)	−0.014 (0.030)		0.035 (0.026)	0.042 (0.026)		0.025 (0.025)	0.025 (0.025)
Number of siblings		0.016 (0.022)	0.018 (0.021)		−0.035* (0.021)	−0.041** (0.021)		0.008 (0.020)	0.008 (0.020)
Father's education (years)		0.007 (0.005)	0.008 (0.005)		0.007 (0.005)	0.006 (0.005)		0.010** (0.004)	0.009** (0.004)
Mother's education (years)		0.007 (0.005)	0.006 (0.005)		0.004 (0.004)	0.006 (0.004)		0.004 (0.004)	0.004 (0.004)
Rural household (dummy, = 1 if yes)		−0.001 (0.027)	−0.002 (0.027)		0.031 (0.029)	0.029 (0.029)		0.020 (0.023)	0.021 (0.023)
Household income level (dummies for four levels)		Yes	Yes		Yes	Yes		Yes	Yes
Class size			0.008* (0.005)			0.005 (0.005)			−0.007*** (0.002)
Subject teacher is headteacher (dummy, = 1 if yes)			0.048 (0.047)			0.093** (0.043)			0.056** (0.024)
Subject teacher is male (dummy, = 1 if yes)			0.073 (0.062)			−0.070 (0.053)			0.016 (0.047)
Subject teacher's education (years)			0.057 (0.041)			−0.037 (0.044)			0.030** (0.015)
Subject teacher's teaching experience (years)			0.004 (0.003)			−0.008*** (0.002)			−0.001 (0.002)
School fixed effects	Yes	Yes	Yes	Yes	Yes	Yes	Yes	Yes	Yes
Constant	0.445 (0.293)	0.217 (0.294)	−1.128 (0.800)	0.561** (0.268)	0.355 (0.276)	0.791 (0.760)	0.154 (0.232)	−0.090 (0.249)	−0.252 (0.338)
*N*	4018	4018	4018	4018	4018	4018	4018	4018	4018
R^2^	0.548	0.550	0.552	0.596	0.598	0.601	0.697	0.699	0.699

Notes: The sample includes 52 schools that randomly assigned newly-enrolled students to 7th-grade classes but did not reassign students in 8th grade. “Household income” levels include “very poor” (reference group), “poor,” “average,” “rich,” and “very rich.” Standard errors in parentheses, clustered at the class level. *p < 0.1, **p < 0.05, ***p < 0.01.

Two findings are notable from the table. Firstly, being (randomly) assigned to a 7th-grade class with high-achieving classmates has a beneficial effect on individual students' academic performance in 8th grade. More specifically, a one-SD increase in classmates' average 7th-grade mathematics test score is associated with an increase of 0.13–0.18 SDs in one's 8th-grade mathematics score (columns 4–6); classmates' average 7th-grade English score has a very similar effect on one's 8th-grade English score (columns 7–9). These effects are within the range of previous estimates, especially those found in East Asian countries.^5^ In contrast, while peer effects on one's Chinese test scores are positive, the effects are not statistically significant. These differences in academic peer effects across subjects may reflect that there is limited room for peer interaction in learning Chinese (a native language) than in learning mathematics (a technical subject) and English (a foreign language). They may also result from the different shapes of learning curves across subjects. For example, Chinese skills may require more time to develop and accumulate than mathematics and English skills; thus, peer effects on individual students' Chinese skills may need a longer time to realize [3].

Secondly, and perhaps more importantly, conditional on school fixed effects, the estimated academic peer effects in the classroom remain quite robust to different empirical specifications. The robustness of empirical findings reported in Table 4 lends further support to our key identifying assumption, echoing the evidence reported in Table 2, Table 3: student/class assignments in 7th grade were indeed done randomly in the focal schools.

For comparison purposes, we repeated the analyses reported in Table 4 with the sample of the 60 CEPS schools whose teachers did not uniformly report random class assignments in 7th grade. Appendix Table A1 reports the results: the peer effects estimated using this sample are generally smaller than those estimated using the sample of schools with random *student/class* assignments in 7th grade (Table 4). This contrast is consistent with the possibility that the 60 schools with potential non-random class assignments sorted incoming 7th graders into 7th-grade classes based on their (previous) academic performance (—Table 2, panel B, has already provided some suggestive evidence for this possibility). There are two reasons for the smaller estimates of peer effects in this sample. Mechanically speaking, with “performance sorting,” classmates are likely to have more similar 7th-grade test scores than schoolmates in different classes. Thus, once individual students' own 7th-grade test scores have been controlled for, the relative importance of their classmates' average 7th-grade test scores in explaining their 8th-grade test scores declines. Econometrically speaking, as suggested in panel B of Table 2, sorting by previous performance creates a much higher correlation between individual students' and their classmates' 7th-grade test scores. This higher correlation, in turn, introduces multicollinearity issues in the models: once individual students' own (previous) test scores have been controlled for, the coefficients of classmates' average test scores become less statistically significant (especially for English test scores). Note that when subject teachers’ characteristics are further included in the models, the estimated classroom academic peer effects vanish (Appendix Table A1).

### 3.2 The role of peer characteristics examined in previous CEPS-based studies

A related question is: Do the academic peer effects discussed above pick up the effects of peer composition in the class? Or do they represent separate effects of peers' academic performance? Recall from the Introduction that by exploiting random class assignments within schools, previous CEPS-based studies have provided important insights into the impact of classroom peer composition, such as proportions of girls [14], migrant children [15,16], primary-school repeaters [21], classmates with alcoholic fathers [19], only-child classmates [17], on individual students' academic performance. While many of these studies provided suggestive evidence that the effects of classmate composition work through a better learning environment (with more high-performing classmates),^6^ none has provided direct evidence on whether classmates’ academic performance indeed serves as a key channel.

The following analysis helps provide an answer. Table 5 examines how adding those peer-composition measures examined in previous CEPS-based studies in our models affects the estimated effects of peer academic performance—for Chinese (panel A), mathematics (panel B), and English (panel C) test scores. Columns 1–5 of Table 5 show that the estimated effects of peer academic performance remained similar after adding previously examined peer-composition measures. Further examining how including other commonly-used peer characteristics (i.e., parental education and family income) may affect our estimation results yields a similar pattern (columns 6–8). These findings suggest that classmates' academic performance exerts an additional and separate effect on individual students’ academic performance rather than merely picking up the peer-composition effects found in previous CEPS-based studies.

**Table 5:** Effects of peer characteristics examined in previous CEPS-based studies.

Outcome variable: standardized 8th-grade test scores	(1)	(2)	(3)	(4)	(5)	(6)	(7)	(8)
	A. Chinese							
Classmates' average 7th-grade Chinese test score	−0.034 (0.069)	−0.010 (0.072)	−0.043 (0.079)	−0.017 (0.071)	−0.033 (0.071)	−0.037 (0.071)	−0.041 (0.077)	−0.043 (0.070)
Proportion of [peers] in the class:								
Girls [14]	Yes							
Migrant children [15,16]		Yes						
Repeaters [21]			Yes					
Classmates with alcoholic fathers [19]				Yes				
Only-child classmates [18]					Yes			
Family backgrounds of Grade-7 classmates:								
Mean years of fathers' education						Yes		
Mean years of mothers' education							Yes	
Proportion of peers in non-poor households								Yes
Controls (full set, with school fixed effects)	Yes	Yes	Yes	Yes	Yes	Yes	Yes	Yes
*N*	4018	4018	4018	4018	4018	4018	4018	4018
R^2^	0.552	0.552	0.548	0.545	0.552	0.557	0.557	0.558
	B. Mathematics							
Classmates' average 7th-grade mathematics test score	0.131** (0.063)	0.141** (0.062)	0.093 (0.060)	0.137** (0.064)	0.133** (0.064)	0.119* (0.063)	0.114* (0.063)	0.121** (0.060)
Proportion of [peers] in the class:								
Girls [14]	Yes							
Migrant children [15,16]		Yes						
Repeaters [21]			Yes					
Classmates with alcoholic fathers [19]				Yes				
Only-child classmates [18]					Yes			
Family backgrounds of 7th-grade classmates:								
Mean years of fathers' education						Yes		
Mean years of mothers' education							Yes	
Proportion of peers in non-poor households								Yes
Controls (full set, with school fixed effects)	Yes	Yes	Yes	Yes	Yes	Yes	Yes	Yes
*N*	4018	4018	4018	4018	4018	4018	4018	4018
R^2^	0.600	0.601	0.601	0.598	0.601	0.606	0.606	0.613
	C. English							
Classmates' average 7th-grade English test score	0.111*** (0.035)	0.118*** (0.032)	0.140*** (0.044)	0.117*** (0.035)	0.124*** (0.036)	0.110*** (0.040)	0.090** (0.039)	0.128** (0.051)
Proportion of [peers] in the class:								
Girls [14]	Yes							
Migrant children [15,16]		Yes						
Repeaters in primary school [21]			Yes					
Classmates with alcoholic fathers [19]				Yes				
Only-child classmates [18]					Yes			
Family backgrounds of 7th-grade classmates':								
Mean years of fathers' education						Yes		
Mean years of mothers' education							Yes	
Proportion of peers in non-poor households								Yes
Controls (full set, with school fixed effects)	Yes	Yes	Yes	Yes	Yes	Yes	Yes	Yes
*N*	4018	4018	4018	4018	4018	4018	4018	4018
R^2^	0.700	0.699	0.699	0.698	0.700	0.709	0.709	0.710

Notes: The sample includes 52 schools that randomly assigned newly-enrolled students to 7th-grade classes but did not reassign students in 8th grade. “Controls” include the full set of control variables (i.e., students' own subject-specific 7th-grade test scores, personal, family, and teacher characteristics, and school fixed effects) reported in Table 4. Standard errors in parentheses, clustered at the class level. *p < 0.1, **p < 0.05, ***p < 0.01.

### 3.3 Potential working channels

Classroom peer effects may also work through multiple channels (besides those examined in previous CEPS-based studies). The CEPS data enable us to explore two potential channels: increased time spent studying and raised confidence in learning. Table 6 reports the results.

**Table 6:** Potential working channels of academic peer effects in the classroom.

Outcome variables:	(1)	(2)	(3)	(4)	(5)	(6)
	A. Time spent studying per day (weekdays)			B. Time spent studying per day (weekends)		
Classmates' average 7th-grade Chinese score	0.083 (0.133)			0.119 (0.092)		
Classmates' average 7th-grade mathematics score		0.042* (0.023)			0.073*** (0.019)	
Classmates' average 7th-grade English score			0.057*** (0.020)			0.057*** (0.021)
Controls (full set, with school fixed effects)	yes	yes	yes	yes	yes	yes
*N*	4018	4018	4018	4018	4018	4018
R^2^	0.190	0.190	0.191	0.182	0.185	0.185
Outcome variables:	C. *Not* feeling difficult in learning specific subjects in 8th grade			D. *Not* feeling difficult in learning specific subjects in 6th grade (falsification test)		
Classmates' average 7th-grade Chinese score	0.008 (0.026)			0.017 (0.014)		
Classmates' average 7th-grade mathematics score		0.068** (0.032)			0.027 (0.022)	
Classmates' average 7th-grade English score			0.058** (0.028)			−0.011 (0.018)
Controls (full set, with school fixed effects)	yes	yes	yes	yes	yes	yes
*N*	4018	4018	4018	4018	4018	4018
R^2^	0.107	0.190	0.187	0.092	0.188	0.187

Notes: The sample includes 52 schools that randomly assigned newly-enrolled students to 7th-grade classes but did not reassign students in 8th grade. Controls include all control variables (i.e., students' own subject-specific 7th-grade test scores, personal, family, and teacher characteristics, and school fixed effects) reported in Table 4. Standard errors in parentheses, clustered at the class level. *p < 0.1, **p < 0.05, ***p < 0.01.

Firstly, given China's competitive high-school admission system, seeing one's classmates perform better, one may decide to spend more time studying to either “catch up” with or even surpass them. To test this prediction, we estimate the effects of classmates' average 7th-grade test scores on one's time spent doing homework for each of the three core subjects in 8th grade. The results suggest that classmates' better academic performance induces individual students to spend more time working on homework assignments—on both weekdays and weekends. Specifically, a one-SD increase in classmates' average 7th-grade test score in a given subject raises one's time spent doing homework by 0.04–0.06 h on weekdays (Table 6, panel A) and 0.06–0.07 h on weekends (Table 6, panel B).

Yet, spending more time doing homework does not necessarily mean that one would actually learn more. Low-achieving students may need to spend more time on homework because of their relatively low learning efficiency. Thus, it is also informative to explore other channels. Another potential channel is students' perceived difficulty in learning (which reflects their “confidence in learning”). We use sampled students' responses to the following survey question, “Do you feel difficulty in learning [subject]? ("subject" = "Chinese", "mathematics", or "English")” to measure their perceived difficulty in learning. As shown in Table 6, Panel C, classmates’ better academic performance in a given subject is associated with a lower level of difficulty a student perceives in learning that subject, which may improve his/her learning efficiency.

Note that the CEPS also asked sampled students to recall their perceived levels of difficulty in learning the three core subjects in 6th grade (the last grade of primary school), which provides an opportunity to perform a falsification test for our quasi-experimental design. If one's classmates in 7th grade were indeed randomly assigned, their academic performance in 7th grade should not have any predictive power for one's perceived difficulty in learning in 6th grade. The results reported in Table 6, Panel D, verify this expectation: the associations between classmates' 7th-grade test scores and one's perceived difficulty in learning in 6th grade are statistically insignificant for all three subjects. Thus, the estimates reported in Panel C of Table 6 can be considered causal. Arguably, it is still possible that students' lowered perceived difficulty in learning is an outcome of their improved academic performance rather than a channel to achieve the latter (—a similar argument applies to their increased learning time). But in that case, these results provide corroborative evidence that significant academic peer effects exist in China's middle school classrooms.

Note also that the above explorations also suggest another line of robustness checks. If improved confidence in learning and increased learning time are driven by better academic performance, then individual students' academic performance may also be driven by high-achieving classmates' confidence in learning and learning time, but not classmates' better academic performance *per se*. To test this possibility, we include classmates' average weekly learning time and confidence in learning (i.e., the proportion of classmates feeling difficulty in learning a subject) in our models. As shown in Appendix Table A2, the estimated effects of classmates’ academic performance remain robust to the inclusion of these two variables, regardless of whether these two variables were measured in 7th grade (odd-numbered columns) or 8th grade (even-numbered columns).

### 3.4 Heterogeneity in classroom peer effects

More insights into how classroom peer effects work may also be learned by examining how these effects vary across different subgroups of students. Thus, we repeated the analyses reported in Table 4, but this time separately by gender, previous academic performance, parental education, family income level, school location, and class size.

Table 7 reports the results, revealing three informative patterns. First, classroom peer effects differ greatly between boys and girls: compared with girls (panel A), boys (panel B) benefit more from peer influence in the classroom, which is consistent with the findings of many previous studies [15,25,26].^7^ Boys may be more sensitive to their learning environment and thus easier to be affected by their classmates [26,27]. No matter what causes these gender differences, it is likely to widen the existing gender gap in educational attainment in China [28] through classroom peer effects. Second, students from families with relatively disadvantaged backgrounds, e.g., less-educated parents (panels F–H) and less wealthy (panels I–J), benefit more from peer interaction. For these students, classmates’ better academic performance serves to compensate for their disadvantaged backgrounds to some extent. Finally, students attending schools presumably of higher quality, e.g., urban schools (panel L) and schools with smaller class sizes (panel N), also gain more from peer interaction, suggesting that school quality and peer effects complement each other in education production. Thus, it is not surprising that academically stronger students (measured by their academic performance in 7th grade (panels *C*–D)) enjoy larger peer effects.

**Table 7:** Heterogeneity in academic peer effects in the classroom.

Outcome variables	(1)	(2)	(3)	(4)	(5)	(6)
	Chinese	Mathematics	English	Chinese	Mathematics	English
	A. Girls			B. Boys		
Classmates' average 7th-grade test score (corresponding subject)	0.070 (0.075)	0.171*** (0.064)	0.107 (0.066)	0.065 (0.082)	0.185** (0.078)	0.096** (0.043)
*N*	1968	1968	1964	2050	2050	2042
R^2^	0.473	0.577	0.683	0.547	0.630	0.683
	C. Baseline subject test score < Median			D. Baseline subject test score ≥ Median		
Classmates' average 7th-grade test score (corresponding subject)	0.048 (0.091)	0.117 (0.106)	0.020 (0.075)	0.117* (0.069)	0.193*** (0.050)	0.184*** (0.045)
*N*	2009	2009	1999	2009	2009	2007
R^2^	0.468	0.475	0.553	0.211	0.278	0.344
	E. Father's education < Median (12 years)			F. Father's education ≥ Median (12 years)		
Classmates' average 7th-grade test score (corresponding subject)	−0.029 (0.067)	0.151** (0.068)	0.149*** (0.041)	−0.111 (0.159)	0.050 (0.097)	0.043 (0.058)
*N*	2972	2972	2961	1042	1042	1041
R^2^	0.549	0.616	0.697	0.584	0.583	0.723
	G. Mother's education < Median (9 years)			H. Mother's education ≥ Median (9 years)		
Classmates' average 7th-grade test score (corresponding subject)	0.011 (0.064)	0.236*** (0.072)	0.200*** (0.047)	−0.129 (0.125)	0.007 (0.096)	−0.060 (0.046)
*N*	2096	2096	2088	1920	1920	1916
R^2^	0.563	0.635	0.693	0.558	0.582	0.719
	I. Family wealth: poor			J. Family wealth: not poor		
Classmates' average 7th-grade test score (corresponding subject)	0.029 (0.065)	0.148** (0.065)	0.154* (0.080)	−0.144 (0.108)	0.103 (0.137)	0.082** (0.033)
*N*	572	572	570	3445	3445	3435
R^2^	0.600	0.670	0.690	0.555	0.598	0.708
	K. Rural schools			L. Urban schools		
Classmates' average 7th-grade test score (corresponding subject)	0.106 (0.087)	0.172 (0.143)	0.064 (0.099)	−0.007 (0.085)	0.322* (0.162)	0.108** (0.042)
*N*	1415	1415	1409	2603	2603	2597
R^2^	0.545	0.589	0.686	0.559	0.610	0.711
	M. Class size ≥ median (47)			N. Class size < median (47)		
Classmates' average 7th-grade test score (corresponding subject)	−0.476* (0.231)	−0.431*** (0.083)	0.001 (0.075)	−0.184 (0.139)	0.176** (0.078)	0.125** (0.053)
*N*	1349	1349	1342	2669	2669	2664
R^2^	0.595	0.665	0.683	0.537	0.577	0.709

Notes: The sample includes 52 schools that randomly assigned newly-enrolled students to 7th-grade classes but did not reassign students in 8th grade. All models include the full set of “control variables” (i.e., students' own subject-specific 7th-grade test scores, personal, family, and teacher characteristics, and school fixed effects) reported in Table 4. In Panel J, the “not poor” group includes the “average,” “rich,” and “very rich” groups. Standard errors in parentheses, clustered at the class level. *p < 0.1, **p < 0.05, ***p < 0.01.

## 4 Discussion and conclusion

Exploiting random class assignments of incoming 7th-graders in 52 middle schools participating in the CEPS, our quasi-experimental analysis performed in this study discovered significant peer effects in China's middle-school classrooms. A one-SD increase in classmates' average 7th-grade mathematics/English test score is associated with an increase of 0.13–0.18/0.11–0.17SDs in one's 8th-grade mathematics/English test score, which implies a social multiplier effect of about 1.2 for mathematics/English learning.^8^ While these effects seem modest, with reference to a recent study on the effect of private tutoring in China [29], the peer effects found in this study (at least those on mathematics test scores) are nearly double those of one year's private mathematics tutoring.

Also discovered are two working channels of classroom academic peer effects: increased time spent studying and raised confidence in learning. Perhaps most importantly, classroom peer effects are found to be heterogeneous across subgroups of students: the effects are more sizable (and thus more statistically significant) for students with relatively disadvantaged family backgrounds, academically stronger students, as well as students attending better schools. While the overall positive peer effects suggest ability tracking as a means to improve learning efficiency (especially for students with relatively disadvantaged family backgrounds), the heterogeneity in peer effects in favor of students enjoying stronger academic ability and higher school quality raises equity concerns.

Despite these important findings, a note on the limitations of our study is in order. First, as pointed out in Section 3, the CEPS data lack detailed information on sampled students' academic performance before middle-school enrollment. Thus, there is a possibility that our estimates of academic peer effects are still tainted with reverse causality operated from one's own academic performance to one's classmates' performance, in that our primary peer-performance measures were recorded roughly two months after students' enrollment in 7th grade. However, as we have argued above, this possibility is likely to be small because two months may be too short for reverse causality to occur. And the inclusion of students' own 7th-grade test scores can help break the potential reverse causality to a large extent. Nonetheless, studies with access to students' pre-enrollment test scores should be conducted to provide more accurate estimates of classroom peer effects.

Second, the CEPS data do not permit us to examine students' behavior in the classroom in response to differences in classmate composition. While we did explore several possible channels through which classroom academic peer effects work, these channels do not provide direct indications of how classmates interact in the classroom. More research investigating students’ classroom behavior and peer interaction is needed to gain further insights into the working channels of classroom peer effects.

Third, also due to data limitations, we only examined peer effects among students in a single grade (8th grade). Yet, the role classmates play may differ greatly across different grades, since the level of difficulty in learning new materials and the approach to grasping these materials would evolve as students advance to a higher grade [25]. Future studies employing follow-up data from the CEPS (or other suitable data) might help discover informative, dynamic patterns of academic peer effects in China's middle school classrooms.

Finally, the measures of peers' academic performance used in this study are defined based on classmate averages. Yet, as pointed out in the recent literature [30,31], students may form peer subgroups within the class—after all, not all classmates will influence an individual student in the same way. As such, our measures of peer academic performance may be tainted with measurement errors, thereby masking some meaningful interactions within peer subgroups in the classroom. Future studies with more advanced research designs and sophisticated empirical methods that can identify students’ actual peer (sub)groups are expected to be fruitful.

Despite these limitations, however, the current study still provides valuable new evidence of the existence, scale, and working channels of classroom academic peer effects that can help deepen our understanding of the nature of education production and inform educational policy in China and in countries alike.

## Author contribution statement

Qihui Chen: Conceived and designed the experiments; Wrote the paper.

Chunchen Pei: Performed the experiments; Analyzed and interpreted the data; Contributed reagents, materials, analysis tools or data; Wrote the paper.

Yuhe Guo: Conceived and designed the experiments; Performed the experiments; Analyzed and interpreted the data.

Shengying Zhai: Performed the experiments; Contributed reagents, materials, analysis tools or data.

## Additional information

Supplementary content related to this article has been published online at [URL].

## Data availability statement

The data that support the findings of this study are available in Chinese National Survey Data.

Archive at http://www.cnsda.org/index.php?r=projects/view&id=72810330 and http://www.cnsda.org/index.php?r=projects/view&id=61662993. The data are also available from the corresponding author, Dr. Qihui Chen (chen1006@umn.edu).

## Declaration of competing interest

The authors declare that they have no known competing financial interests or personal relationships that could have appeared to influence the work reported in this paper.

## Figures and Tables

**Appendix Table A1:** Effects of classmates' average 7th-grade test scores on individual students' 8th-grade test scores (60 schools with non-random class assignments)

Outcome variable: standardized 8th-grade test scores	(1)	(2)	(3)	(4)	(5)	(6)	(7)	(8)	(9)
	Chinese			Mathematics			English		
Classmates' average 7th-grade Chinese test score	0.008 (0.054)	0.008 (0.054)	−0.006 (0.056)						
Classmates' average 7th-grade mathematics test score				0.130*** (0.045)	0.130*** (0.045)	0.040 (0.046)			
Classmates' average 7th-grade English test score							0.093* (0.048)	0.089* (0.049)	−0.047 (0.047)
Own 7th-grade test scores (subject-specific)	0.567*** (0.016)	0.566*** (0.016)	0.564*** (0.017)	0.578*** (0.023)	0.577*** (0.023)	0.564*** (0.024)	0.666*** (0.021)	0.666*** (0.021)	0.655*** (0.023)
Age (months)	−0.002 (0.001)	−0.002 (0.001)	−0.002 (0.001)	−0.004** (0.001)	−0.004*** (0.001)	−0.003** (0.001)	−0.004*** (0.001)	−0.004** (0.001)	−0.004** (0.002)
Boy (dummy, = 1 if yes)	−0.265*** (0.021)	−0.267*** (0.021)	−0.270*** (0.022)	−0.092*** (0.022)	−0.096*** (0.023)	−0.092*** (0.025)	−0.191*** (0.024)	−0.201*** (0.025)	−0.200*** (0.027)
Cognitive ability test score (standardized)	0.266*** (0.021)	0.266*** (0.021)	0.264*** (0.022)	0.300*** (0.024)	0.300*** (0.024)	0.302*** (0.025)	0.203*** (0.023)	0.200*** (0.023)	0.211*** (0.023)
Birth order		0.004 (0.018)	0.010 (0.019)		0.016 (0.021)	0.007 (0.022)		0.025 (0.019)	0.029 (0.020)
Number of siblings		−0.001 (0.017)	−0.003 (0.018)		−0.004 (0.018)	−0.000 (0.019)		−0.029* (0.015)	−0.030* (0.017)
Father's education (years)		0.009* (0.005)	0.007 (0.005)		0.008 (0.005)	0.007 (0.005)		0.015*** (0.004)	0.016*** (0.004)
Mother's education (years)		−0.007* (0.004)	−0.006 (0.004)		−0.002 (0.004)	−0.003 (0.005)		−0.006* (0.003)	−0.006* (0.004)
Rural household (dummy, = 1 if yes)		−0.020 (0.025)	−0.006 (0.028)		0.036 (0.024)	0.038 (0.026)		0.039 (0.024)	0.042 (0.026)
Household income level (dummies for four levels)		Yes	Yes		Yes	Yes		Yes	Yes
Class size			0.011*** (0.004)			0.011** (0.004)			0.011*** (0.004)
Subject teacher is headteacher (dummy, = 1 if yes)			0.014 (0.041)			−0.030 (0.036)			0.263*** (0.066)
Subject teacher is male (dummy, = 1 if yes)			−0.033 (0.072)			−0.084*** (0.031)			−0.071 (0.064)
Subject teacher's education (years)			−0.115*** (0.032)			−0.105*** (0.029)			−0.037 (0.028)
Subject teacher's teaching experience (years)			−0.010*** (0.003)			−0.004 (0.003)			−0.005* (0.003)
School fixed effects	Yes	Yes	Yes	Yes	Yes	Yes	Yes	Yes	Yes
Constant	0.422* (0.236)	0.450* (0.252)	1.866*** (0.560)	0.571** (0.229)	0.485* (0.246)	1.662*** (0.471)	0.724*** (0.251)	0.564** (0.265)	0.602 (0.638)
*N*	5099	5099	5099	5099	5099	5099	5099	5099	5099
R^2^	0.529	0.531	0.536	0.550	0.551	0.542	0.618	0.619	0.616

Notes: The sample includes 60 schools with potential non-random class assignments in 7th grade. “Household income” levels include “very poor” (reference group), “poor,” “average,” “rich,” and “very rich.” Standard errors in parentheses, clustered at the class level. *p < 0.1, **p < 0.05, ***p < 0.01.

**Appendix Table A2:** Further robustness checks

Outcome variable: standardized Grade-8 test scores	(1)	(2)	(3)	(4)	(5)	(6)
	Chinese		Mathematics		English	
Classmates' average 7th-grade Chinese test score	−0.008 (0.065)	0.026 (0.086)				
Classmates' average 7th-grade mathematics test score			0.132* (0.077)	0.196*** (0.060)		
Classmates' average 7th-grade English test score					0.173*** (0.050)	0.171*** (0.058)
Classmates' average time spent studying per day in 7th grade	−0.071 (0.050)		−0.069 (0.064)		−0.029 (0.037)	
Classmates' average time spent studying per day in 8th grade		−0.025 (0.075)		−0.246*** (0.059)		−0.017 (0.042)
Proportion of classmates not feeling difficult in learning specific subjects in 7th grade	−0.092 (0.150)		−0.034 (0.209)		−0.546*** (0.134)	
Proportion of classmates not feeling difficult in learning specific subjects in 8th grade		−0.414** (0.197)		−0.327 (0.216)		−0.178 (0.155)
7th grade test scores (subject-specific)	0.579*** (0.020)	0.578*** (0.019)	0.625*** (0.020)	0.619*** (0.020)	0.768*** (0.017)	0.764*** (0.017)
Age (months)	−0.002 (0.002)	−0.002 (0.002)	−0.003 (0.002)	−0.002 (0.002)	0.000 (0.001)	−0.001 (0.001)
Boy (dummy, = 1 if yes)	−0.226*** (0.026)	−0.223*** (0.025)	−0.103*** (0.026)	−0.102*** (0.026)	−0.133*** (0.020)	−0.135*** (0.021)
Cognitive ability test score (standardized)	0.270*** (0.024)	0.265*** (0.023)	0.297*** (0.020)	0.302*** (0.020)	0.145*** (0.018)	0.135*** (0.018)
Birth order	−0.026 (0.031)	−0.027 (0.030)	0.039 (0.027)	0.044* (0.026)	0.015 (0.025)	0.018 (0.026)
Number of siblings	0.024 (0.023)	0.028 (0.022)	−0.034 (0.022)	−0.036* (0.019)	0.008 (0.019)	0.013 (0.020)
Father's education (years)	0.007 (0.005)	0.006 (0.005)	0.005 (0.005)	0.007 (0.005)	0.008* (0.004)	0.010** (0.004)
Mother's education (years)	0.006 (0.005)	0.006 (0.005)	0.008* (0.004)	0.006 (0.004)	0.006 (0.004)	0.004 (0.004)
Rural household (dummy, = 1 if yes)	−0.014 (0.029)	−0.019 (0.027)	0.030 (0.030)	0.027 (0.030)	0.010 (0.025)	0.009 (0.023)
Household income level (dummies for four levels)	Yes	Yes	Yes	Yes	Yes	Yes
Class size	0.008* (0.004)	0.009** (0.004)	0.008 (0.005)	0.012** (0.005)	−0.006 (0.004)	−0.008* (0.004)
Subject teacher is headteacher (dummy, = 1 if yes)	0.053 (0.046)	0.026 (0.046)	0.045 (0.044)	0.021 (0.043)	0.074*** (0.025)	0.059** (0.027)
Subject teacher is male (dummy, = 1 if yes)	0.066 (0.060)	0.109* (0.064)	−0.116** (0.049)	−0.162*** (0.044)	0.013 (0.056)	−0.008 (0.055)
Subject teacher's education (years)	0.096* (0.052)	0.132** (0.051)	−0.029 (0.045)	−0.067 (0.043)	0.030 (0.018)	0.029* (0.016)
Subject teacher's teaching experience (years)	0.004 (0.003)	0.007** (0.003)	−0.008*** (0.002)	−0.009*** (0.002)	−0.003 (0.002)	−0.001 (0.003)
School fixed effects	Yes	Yes	Yes	Yes	Yes	Yes
Constant	−1.518 (1.007)	−2.082** (0.953)	0.582 (0.826)	1.513* (0.766)	0.061 (0.445)	0.019 (0.397)
*N*	4018	4018	4018	4018	4018	4018
R^2^	0.546	0.547	0.608	0.609	0.707	0.699

Notes: The sample includes 52 schools that randomly assigned new-enrolled students to 7th-grade classes but did not reassign students in 8th grade. “Household income” levels include “very poor” (reference group), “poor,” “average,” “rich,” and “very rich.” Standard errors in parentheses, clustered at the class level. *p < 0.1, **p < 0.05, ***p < 0.01.

## Footnotes

1 Some studies also examined the effects of peer characteristics on individual students' non-cognitive outcomes [13,20].

2 Ten of the 112 schools have only one class in a target grade; these classes are all included in the sample.

3 China's middle schools use various methods to assign incoming students to different classes at the beginning of 7th grade. In some schools, students are assigned based on their entrance test scores or academic performance in the primary school. Recently, more and more schools have started to assign incoming 7th graders to different 7th-grade classes randomly. The random student/class assignment method is strongly promoted by China's Ministry of Education to ensure equal and fair opportunities among students [24]. Schools that adopted random student/class assignments in 7th grade typically use a computer program incorporating data on incoming 7th graders' gender, residential registration status (hukou), primary school attended, and other factors to find a suitable assignment algorithm, which ensures educational resources are allocated equally across different classes.

4 According to official statistics [24], in China's middle schools, the average class size was 48 in 2013, and Chinese, mathematics, and English teachers completed on average 15.9, 15.8 and 15.9 years, respectively.

5 In the United States, one study found classroom peer effects of 0.15–0.24 SDs [5]. Another study found peer effects of 0.01–0.07 SDs [32]. In Brazil, classroom peer effects were found to be 0.11–0.17 SDs [33]. Peer effects found in East Asian countries tend to be larger. For example, A study done in South Korea found peer effects of 0.25–0.34 SDs [34]. A study done in China found peer effects of 0.10–0.52 SDs [3].

6 It has been found that girls outperformed boys in all three core subjects [14]; compared to students who never repeated a grade in primary schools, repeaters scored significantly lower in all three core subjects [21]; students with alcoholic fathers also had lower academic achievement [19]; students with no siblings scored significantly higher in mathematics compared with students who had siblings [21]. These findings suggest that having proportionally more girls, non-repeaters, classmates with non-alcoholic fathers, and only-child students in the class helps provide a better learning environment for all students in the class.

7 Note that not all existing studies found such a pattern. For example, in England peer effects were found to be larger for girls than boys in middle schools [9]; in a Chinese college, peer effects were only found for female students but not for male students [35].

8 Peer effects create social multipliers [36]. For example, if the peer effect in math is b (<1), a one-SD increase in peers' average math test scores increases one's math test score by b SDs, which would lead to another b^2^-SD increase in test scores in the next “round”, etc. Hence, the total effect would be 1+ b + b^2^ + … = 1/(1-b). When b = 0.18, the social multiplier effect is 1/(1–0.18) = 1.22.
