# Supplementary material for: What drives academic peer effects in middle school classrooms in China: Peer composition or peer performance?
Source: Heliyon. 2023 Jun 1;9(6):e16840. doi: 10.1016/j.heliyon.2023.e16840 (PMC10258448; doi:10.1016/j.heliyon.2023.e16840)
Supplement: Multimedia component 1 [file mmc1.pdf]

Class: __________ Student Name:____________

Name of Homeroom Teacher: __________ Name of School: ___________

Location of School: County (District):__________ City: ___________

Province (Autonomous Region/Municipality):___________

China Education Panel Survey
Academic Year 2013-2014
Student Questionnaire for Grade 7

Students answering this questionnaire do not need to fill in the blanks in the box below.

1. Code of Questionnaire: [____|____|____|____|____|____]

2. Code of County/District: [____|____|____|____|____|____]

3. Verifier: ____________________

Hi Dear Student,

China Education Panel Survey is the first large-scale, nationwide, and longitudinal social survey of junior high students in China. The purpose of the CEPS is to provide detailed and reliable data for the research of the current situation and future development of China's primary education.

CEPS is jointly conducted by the National Survey Research Center (NSRC) at Renmin University of China and academic institutions throughout the country. During the academic year of 2013-2014, we are going to survey nearly 20,000 students as well as their parents, homeroom teachers, subject teachers and school administrators. Through scientific sampling, the whole class of yours have been chosen as subjects of this survey. Therefore, we are asking you here to answer all the questions in this questionnaire truthfully based on your own ideas and experiences.

There are neither right nor wrong answers to these questions and your answers will not be evaluated as part of your academic record. We pledge here that under *the Statistics Law of the People's Republic of China*, we will hold all the information you are going to provide in strict confidence and it will never be given away to any individual or institution. Thank you for your cooperation.

When answering a multiple-choice question, please circle the number of your choice; when answering a blank-filling question, please write down words or numbers on the lines in the square brackets.

National Survey Research Center (NSRC), Renmin University of China

April, 2014

Part A: Personal Background

A1. Your sex is:

1. Male   2. Female

A2. Your date of birth is: [__|__|__|__] [__|__] ([YYYY] [MM])

A3. Your ethnic nationality is:

1. The Han nationality   2. The Mongol nationality
3. The Manchu nationality   4. The Hui nationality
5. The Tibetan nationality   6. The Zhuang nationality
7. The Uygur nationality   8. Other (Please specify: __________)

A4. Your birthplace is:

1. In the local county/district
2. Not in the local county/district
3. Not clear

A5. The location of your Hukou[1] at present is:

1. In the local county/district
2. Not in the local county/district:
   County/district: [_________], City: [_________],
   Province (Autonomous Region/Municipality): [_________]

A6. What is the type of your Hukou at present? (Agricultural Hukou refers to rural Hukou, a record that identifies a person as a rural resident. Non-agricultural Hukou refers to urban Hukou. Residential Hukou is a general record of residence assigned to all residents of a certain region, regardless of rural or urban background)

1. Agricultural Hukou   2. Non-agricultural Hukou
3. Residential Hukou   4. I have no Hukou

A7. How old were you when you came to this county/district?

[__|__] years old (Please fill in 00 if you are local born.)

A8. Where do you live at present?

1. In the local county/district   2. Not in the local county/district

A9. How is your birthweight?

1. Light   2. Normal   3. Heavy

Your exact birthweight is [___|___]. [__] jin

(Please notice that the unit of the weight is kilogram, and 1 decimal digit is reserved. Please fill in 00.0 if you are not clear about your exact birthweight.)

A10. Have you ever been seriously ill before you went to elementary school?

1. Yes, I have.   2. No, I haven't.   3. Not Clear.

[1] Hukou refers to a record of household registration system in China that identifies a person as a resident of an area.

A11. Which one of the following best describes the financial conditions of your family before you went to elementary school?

1. Very poor 2. Somewhat poor 3. Moderate
4. Somewhat rich 5. Very rich 6. Not clear

A12. How much do you agree with each of the following statements about your experiences in GRADE 6?

	Strongly disagree	Somewhat disagree	Somewhat agree	Strongly agree
I would try my best to go to school even if I was not feeling very well or I had other reasons to stay at home.	1	2	3	4
I would try my best to finish even the homework I dislike.	1	2	3	4
I would try my best to finish my homework, even if it would take me quite a long time.	1	2	3	4
I was able to express myself clearly.	1	2	3	4
I was able to give quick responses.	1	2	3	4
I was a fast learner.	1	2	3	4
I was curious about new stuff.	1	2	3	4

A13. Your current height is: [__|__|__] centimeters.

A14. Your current weight is: [__|__|__] jin (Please notice that the unit of the weight is 'kilogram' rather than 'jin')

A15. Are you short-sighted?

1. Yes, I am.
   The strength of my left eyeglass is [__|__|__] degrees[2].
   The strength of my right eyeglass is [__|__|__] degrees.
2. Yes, I am. But I am not clear about the strength of my glasses/lens.
3. No, I am not.

A16. Have you been hospitalized over the last year?

1. Yes, I have. 2. No, I haven't.

A17. Which one of the following best describes your general health condition at present?

1. Very poor 2. Not very good 3. Moderate 4. Good 5. Very good

A18. Do you have the feelings below in the last seven days?

---

[2] Degree is the unit of measurement of optical power of lens or glasses used in China, 100 degrees of short-sightedness = -1dioptre.

	Never	Seldom	Sometimes	Often	Always
Feeling blue	1	2	3	4	5
Depressed	1	2	3	4	5
Unhappy	1	2	3	4	5
Not enjoying life	1	2	3	4	5
Sad	1	2	3	4	5

(Please continue to finish Part B.)

Part B: Family Background

B1. Are you the only child of your family?

1. Yes, I am. **(SKIP TO B4)**
2. No, I am not.

B2. How many FULL OR HALF siblings do you have? (Please fill in 0 if you don't have any sibling.)

[___] elder brother(s)
[___] younger brother(s)
[___] elder sister(s)
[___] younger sister(s)

B3. If the Hukou of your family is not in the local county/district, please answer: do you have any sibling(s) attending schools located in your hometown?

1. The Hukou of my family is in the local county/district.
2. No, I don't have any.
3. Yes, I do. And the eldest one is in grade [___] at present.

B4. Which of the following people live in the same household with you at present? (Please mark all that apply.)

1. Mother
2. Father
3. Full or half sibling(s)
4. Grandparent(s) on mother's/father's side
5. Other relative(s)
6. Other non-relative(s)

B5. Which of the following immediate relatives of yours do not live in the same household with you at present? (Please mark all that apply.)

1. Mother
2. Father
3. Full or half sibling(s)
4. All my immediate relatives (if there is any) live with me.

B6. What is the highest education level your mother has completed?

1. None
2. Finished elementary school
3. Junior high school degree
4. Technical secondary school or technical school degree
5. Vocational high school degree
6. Senior high school degree
7. Junior college degree
8. Bachelor degree
9. Master degree or higher

B7. What is the highest education level your father has completed?

1. None
2. Finished elementary school
3. Junior high school degree
4. Technical secondary school or technical school degree
5. Vocational high school degree
6. Senior high school degree
7. Junior college degree
8. Bachelor degree
9. Master degree or higher

B8. What are the occupations of your parents?

	Mother	Father
Government official, staff of public institutions, civil servant	1	1
Middle/Senior management personnel of enterprises/corporations	2	2
Teacher, engineer, doctor, lawyer	3	3
Technical worker (including driver)	4	4
Ordinary staff or worker in production or manufacturing industry	5	5
Ordinary staff or worker in business or service industry	6	6
Self-employed worker	7	7
Peasant	8	8
Unemployed or laid-off worker	9	9
Other	(Please specify:________)	(Please specify:________)

B9. Which one of the following best describes the financial conditions of your family at present?

1. Very poor 2. Somewhat poor 3. Moderate 4. Somewhat rich 5. Very rich

B10. Do you agree with the following statements?

	No, I don't agree	Yes, I agree
My father often gets drunk.	1	2
My parents quarrel a lot.	1	2
My parents get along very well.	1	2

B11. Do you have a writing desk of your own at home?

1. Yes, I do. 2. No, I don't.

B12. How many books do your family own? (not including textbooks or magazines)

1. Very few 2. Not many 3. Some
4. Quite a few 5. A great number

B13. Do your family own a computer and have an access to the Internet?

0. No, we don't.
1. We have a computer but no access to the Internet.
2. Yes, we have both.

B14. How much time did you spend on homework assigned by your teachers at school last week? (Please fill in 00 hours and 00 minutes if no time is spent on the homework.)

[__|__] hours [__|__] minutes (on average) from Monday to Friday
[__|__] hours [__|__] minutes (on average) at weekends

B15. How much time ON AVERAGE did you spend on the following extra-curricular activities from Monday to Friday last week? (Please fill in 00 hours 00 minutes if no time is spent on these activities.)

	From Monday to Friday (on average per day)
Doing homework assigned by parents or cram school	[__|__] hours [__|__] minutes
Taking cram school courses (related to schoolwork)	[__|__] hours [__|__] minutes
Playing sports	[__|__] hours [__|__] minutes
Reading (not including textbooks)	[__|__] hours [__|__] minutes
Watching TV	[__|__] hours [__|__] minutes
Surfing on the Internet or playing video games	[__|__] hours [__|__] minutes
Helping your parents with housework	[__|__] hours [__|__] minutes

B16. How much time ON AVERAGE did you spend on the following extra-curricular activities LAST WEEKEND? (Please fill in 00 hours 00 minutes if no time is spent on these activities)

	At weekends (on average per day)
Doing homework assigned by parents or cram school	[__|__] hours [__|__] minutes
Taking cram school courses (related to schoolwork)	[__|__] hours [__|__] minutes
Playing sports	[__|__] hours [__|__] minutes
Reading (not including textbooks)	[__|__] hours [__|__] minutes
Watching TV	[__|__] hours [__|__] minutes
Surfing on the Internet or playing video games	[__|__] hours [__|__] minutes
Helping your parents with housework	[__|__] hours [__|__] minutes

B17. How often do you do the following either alone or with your schoolmates?

	Never	Once a year	Once every half year	Once a month	More than once a month
Visiting museums, zoos, science museums, etc.	1	2	3	4	5
Going out to watch movies, shows, sports games, etc.	1	2	3	4	5

B18. How much time on average do you sleep every night?

[__|__] hours [__|__] minutes

B19. What kind of extra-curricular courses do you take? (Please mark all that apply.)

0. None
1. Mathematical Olympiad
2. Ordinary Mathematics (not including Mathematical Olympiad)
3. Chinese/Chinese Composition Writing
4. English
5. Painting or drawing
6. Calligraphy
7. Music/Musical instrument
8. Dancing
9. Chess
10. Sports
11. Other (Please specify: __________)

B20. What hobbies do you have? (Please mark all that apply.)

0. None
1. Playing musical instruments (Please specify: __________)
2. Vocal practices/Singing/Dancing/Acting
3. Calligraphy
4. Painting or drawing/Animation
5. Chess (Please specify: __________)
6. Sports (Please specify: __________)
7. Other (Please specify: __________)

B21. What do you usually do in winter and summer vacations? (Please mark all that apply.)

1. Taking cram school courses (related to schoolwork)
2. Attending summer/winter camps
3. Helping parents out
4. Going back to hometown
5. Staying at home
6. Other (Please specify: __________)

B22. How often did your parents do the following to check up on your study last week?

	Never	One or two days	Three or four days	Almost everyday
Checking up on your homework	1	2	3	4
Giving instruction on your homework	1	2	3	4

B23. Do your parents care and are they strict with you about the following?

	They don't care.	They do care about it, but are not strict.	They are very strict about it.
Your homework and examination	1	2	3

Your behavior at school	1	2	3
Attendances at school everyday	1	2	3
Time when you get home everyday	1	2	3
Whom you make friends with	1	2	3
Your dress style	1	2	3
Time you spend on the Internet	1	2	3
Time you spend on watching TV	1	2	3

B24. How often do your parents discuss the following with you?

	**Your mother**			**Your father**		
	**Never**	**Sometimes**	**Often**	**Never**	**Sometimes**	**Often**
Things happened at school	1	2	3	1	2	3
The relationship between you and your friends	1	2	3	1	2	3
The relationship between you and your teachers	1	2	3	1	2	3
Your feelings	1	2	3	1	2	3
Your worries and troubles	1	2	3	1	2	3

B25. How is the general relationship between you and your parents?

	**Not close**	**Not too close nor too far**	**Very close**
Relationship between you and your mother	1	2	3
Relationship between you and your father	1	2	3

B26.Who will be the first one for you to turn to in the following situations?

	**Schoolmates or good friends**	**Parents**	**A relative of yours**	**Teachers**	**No one**
When you want to chat with someone	1	2	3	4	5
When you are in trouble	1	2	3	4	5
When you need help	1	2	3	4	5

B27.What language do you usually speak with your parents?

1. Mandarin Chinese
2. Sometimes dialect of my hometown, sometimes mandarin Chinese
3. Dialect of my hometown
4. Other (Please specify: __________)

B28. How often do you do the following with your parents?

	Never	Once a year	Once every half year	Once a month	Once a week	More than once a week
Having dinner	1	2	3	4	5	6
Reading	1	2	3	4	5	6
Watching TV	1	2	3	4	5	6
Playing sports	1	2	3	4	5	6
Visiting museums, zoos, science museums, etc.	1	2	3	4	5	6
Going out to watch movies, shows, sports games, etc.	1	2	3	4	5	6

B29. How much do you think your parents have done for you?

1. Very little
2. Not very much
3. Not too much nor too little
4. A fair amount
5. A great deal

B30. What is your parents' requirement on your academic record?

1. Being one of the top five of your class
2. Above the average
3. About the average
4. No special requirement

B31. What is the highest level of education your parents expect you to receive?

1. Drop out now
2. Graduate from junior high school
3. Go to technical secondary school or technical school
4. Go to vocational high school
5. Go to senior high school
6. Graduate from junior college
7. Get a bachelor degree
8. Get a Master degree
9. Get a Doctor degree
10. They don’t care

B32. How do you feel about such expectation?

1. Not stressed at all
2. Not very stressed
3. Normal
4. Somewhat stressed
5. Very stressed

B33. What kind of job do your parents most expect you to do in the future?

1. Government official, staff of public institutions, civil servant
2. Manager or administrator of enterprises/corporations
3. Scientist/engineer
4. Teacher/doctor/lawyer
5. Designer
6. Artistic performer/actor/host
7. Professional athlete
8. Technical worker (including driver)
9. Other (Please specify: __________)
10. They don't care
11. Not clear

B34. Where do you think your parents most expect you to live and work in the future?

1. In rural area
2. In small or medium cities
3. In big cities, such as Beijing/Shanghai/Guangzhou
4. Abroad
5. They don't care
6. Not clear

B35. Are your parents confident about your future?

1. Not confident at all
2. Not so confident
3. Somewhat confident
4. Very confident

(Please continue to finish Part C)

Part C: School Experiences and Activities

C1. Have you ever gone to a kindergarten/pre-school since you were three years old?
1. Yes, since [__] years old. 2. No, I haven't.

C2. When did you start to go to elementary school?
At [__] years old

C3. From grade 1 to grade 6, how many times have you skipped a grade?
[__] times (Please fill in 0 if you have never skipped a grade.)

C4. From grade 1 to grade 6, how many times have you repeated a grade?
[__] times (Please fill in 0 if you have never repeated a grade.)

C5. From grade 1 to grade 6, how many times have you suspended your schooling? (Suspension of schooling refers to at least one month of absence from school)
[__] times (Please fill in 0 if you have never suspended your schooling.)

C6. From grade 1 to grade 6, how many times have you transferred to a different school?
[__] times (Please fill in 0 if you have never transferred to a different school.)

C7. Have you ever taken English courses in your elementary school?
1. Yes, since grade [___] 2. No, I haven't.

C8. How did your academic record rank in your class when you were in grade 6?
I ranked about [___|___] in my class.

C9. Did you take an entrance examination/placement test when you entered your current school?
1. Yes, I did. 2. No, there's no such test.

C10. Think back when you were in GRADE 6, were the following courses difficult for you?

	Very difficult	A bit difficult	Not very difficult	Not difficult at all	I did not take this course in Grade 6.
Mathematics	1	2	3	4	5
Chinese	1	2	3	4	5
English	1	2	3	4	5

C11. AT PRESENT, are the following courses difficult for you?

	Very difficult	A bit difficult	Not very difficult	Not difficult at all
Mathematics	1	2	3	4
Chinese	1	2	3	4
English	1	2	3	4

C12. How does your academic record rank in your class at present?

1. Near the bottom 2. Below the average 3. About the average
4. Above the average 5. Around the top

C13. How much do you agree with each of the following statements about the main subjects?

	Strongly disagree	Somewhat disagree	Somewhat agree	Strongly agree
Mathematics helps a lot with my future development.	1	2	3	4
Chinese helps a lot with my future development.	1	2	3	4
English helps a lot with my future development.	1	2	3	4
My mathematics teacher always asks me to answer questions in class.	1	2	3	4
My Chinese teacher always asks me to answer questions in class.	1	2	3	4
My English teacher always asks me to answer questions in class.	1	2	3	4
My mathematics teacher always praises me.	1	2	3	4
My Chinese teacher always praises me.	1	2	3	4
My English teacher always praises me.	1	2	3	4

C14. Do you agree that boys are better at mathematics than girls?

1. Yes, I do. 2. No, I don't.

C15. Do your parents agree that boys are better at mathematics than girls?

1. Yes, they do. 2. No, they don't.

C16. Do most people around you agree that boys are better at mathematics than girls?

1. Yes, they do. 2. No, they don't.

C17. How much do you agree with each of the following statements about your school life?

	Strongly disagree	Somewhat disagree	Somewhat agree	Strongly agree
I am always late for class.	1	2	3	4
I always skip classes.	1	2	3	4
My parents always receive criticism on me from my teacher.	1	2	3	4
My homeroom teacher always praises me.	1	2	3	4

My homeroom teacher always criticizes me.	1	2	3	4
Most of my classmates are nice to me.	1	2	3	4
I think I am easy to get along with.	1	2	3	4
My class is in good atmosphere.	1	2	3	4
I often take part in school/class activities.	1	2	3	4
I feel close to people in this school.	1	2	3	4
I feel bored in this school.	1	2	3	4
I hope that I could transfer to another school.	1	2	3	4

C18. Do you live in school at night from Monday to Thursday?

1. Yes, I do.   2. No, I don't.

C19. How many best friends do you have?

[__|__] friends

C20. Please write down NAMES of 5 of your best friends truthfully in the first column of the table below, and then fill in the blanks with number that best describes their conditions. (Please write down as many best friends as you have if there are less than 5.)

**Names of 5 of your best friends**	**Sex<br>1. Male<br>2. Female**	**Location of Hukou<br>1. In the local county/district<br>2. Not in the local county/district**	**Does he/she attend the same school with you?<br>1. Yes.<br>2. No.**	**Is he/she in the same class with you?<br>1. Yes.<br>2. No.**
1____________	[____]	[____]	[____]	[____]
2____________	[____]	[____]	[____]	[____]
3____________	[____]	[____]	[____]	[____]
4____________	[____]	[____]	[____]	[____]
5____________	[____]	[____]	[____]	[____]

C21. How many of your best friends mentioned above fit in the following descriptions?

	**None of them**	**One or two of them**	**Most of them**
Doing well in academic performance	1	2	3
Studying hard	1	2	3
Expecting to go to college	1	2	3

Skipping classes	1	2	3
Criticized or punished for violating school rules	1	2	3
Always fighting with others	1	2	3
Smoking or drinking alcohol	1	2	3
Always going to net bars or video arcade	1	2	3
Having had or is having a romance	1	2	3
Dropped out of school	1	2	3

C22. What is the highest level of education you expect yourself to receive?

1. Drop out now
2. Graduate from junior high school
3. Go to technical secondary school or technical school
4. Go to vocational high school
5. Go to senior high school
6. Graduate from junior college
7. Get a bachelor degree
8. Get a Master degree
9. Get a Doctor degree
10. I don’t care

C23. What kind of job do you most expect to do in the future?

1. Government official, staff of public institutions, civil servant
2. Manager or administrator of enterprises/corporations
3. Scientist/engineer
4. Teacher/doctor/lawyer
5. Designer
6. Artistic performer/actor/host
7. Professional athlete
8. Technical worker (including driver)
9. Other (Please specify: __________)
10. I don’t care

C24. Where do you most expect to live and work when you grow up?

1. In rural area
2. In medium or small cities
3. In big cities, such as Beijing/Shanghai/Guangzhou
4. Abroad
5. I don’t care

C25. Are you confident in your future?

1. Not confident at all   2. Not so confident
3. Somewhat confident   4. Very confident

C26. Do you think your classmates from the local county/district will do the following to those who are not from the local county/district? (Please give your answers based on your assumption if you don't have any classmate from non-local counties/districts.)

	**Will they do this to students from other *urban* counties/districts?**<br>**1. Yes, they will.**<br>**2. No, they won't.**	**Will they do this to students from other *rural* counties/districts?**<br>**1. Yes, they will.**<br>**2. No, they won't.**
Making complaints or offensive remarks about them	[__]	[__]
Playing together with them	[__]	[__]
Discussing questions related to schoolwork	[__]	[__]
Making friends with them	[__]	[__]

(Please continue to finish Part D)

Part D: Contact Information

We hope to continue the tracking of your growth and improvement in the next year by follow-up surveys. Please leave the contact information of yours and that of your parents, so that we could keep in touch with you:

D1. Your contact information:
Your QQ number: _______________
Your mobile phone number: _________________

D2. Your family's telephone number:_________________

D3. Your father's contact information:
Name: _______________ Mobile phone number: _________________

D4. Your mother's contact information:
Name: _______________ Mobile phone number: _________________

This is the end of the questionnaire. Thank you for your engagement and cooperation! We wish you a happy life and a successful future!

Class: _________ Student Name: ____________

Name of Homeroom Teacher: ___________ Name of School: ____________

Location of School: County (District):___________ City: ____________

Province (Autonomous Region/Municipality):____________

China Education Panel Survey
Academic Year 2013-2014
Student Questionnaire for Grade 9

Students answering this questionnaire do not need to fill in the blanks in the box below.

1. Code of Questionnaire: [____|____|____|____|____|____]

2. Code of County/District: [____|____|____|____|____|____]

3. Verifier: ______________________

Hi Dear Student,

China Education Panel Survey is the first large-scale, nationwide, and longitudinal social survey of junior high students in China. The purpose of the CEPS is to provide detailed and reliable data for the research of the current situation and future development of China's primary education.

CEPS is jointly conducted by the National Survey Research Center (NSRC) at Renmin University of China and academic institutions throughout the country. During the academic year of 2013-2014, we are going to survey nearly 20,000 students as well as their parents, homeroom teachers, subject teachers and school administrators. Through scientific sampling, the whole class of yours have been chosen as subjects of this survey. Therefore, we are asking you here to answer all the questions in this questionnaire truthfully based on your own ideas and experiences.

There are neither right nor wrong answers to these questions and your answers will not be evaluated as part of your academic record. We pledge here that under *the Statistics Law of the People's Republic of China*, we will hold all the information you are going to provide in strict confidence and it will never be given away to any individual or institution. Thank you for your cooperation.

When answering a multiple-choice question, please circle the number of your choice; when answering a blank-filling question, please write down words or numbers on the lines in the square brackets.

National Survey Research Center (NSRC), Renmin University of China

April, 2014

Part A: Personal Background

A1. Your sex is:

1. Male   2. Female

A2. Your date of birth is: [__|__|__|__] [__|__] ([YYYY] [MM])

A3. Your ethnic nationality is:

1. The Han nationality   2. The Mongol nationality
3. The Manchu nationality   4. The Hui nationality
5. The Tibetan nationality   6. The Zhuang nationality
7. The Uygur nationality   8. Other (Please specify: __________)

A4. Your birthplace is:

1. In the local county/district
2. Not in the local county/district
3. Not clear

A5. The location of your Hukou[1] at present is:

1. In the local county/district
2. Not in the local county/district:
   County/district: [_________], City: [_________],
   Province (Autonomous Region/Municipality): [_________]

A6. What is the type of your Hukou at present? (Agricultural Hukou refers to rural Hukou, a record that identifies a person as a rural resident. Non-agricultural Hukou refers to urban Hukou. Residential Hukou is a general record of residence assigned to all residents of a certain region, regardless of rural or urban background)

1. Agricultural Hukou   2. Non-agricultural Hukou
3. Residential Hukou   4. I have no Hukou

A7. How old were you when you came to this county/district?

[__|__] years old (Please fill in 00 if you are local born.)

A8. Where do you live at present?

1. In the local county/district   2. Not in the local county/district

A9. How is your birthweight?

1. Light   2. Normal   3. Heavy

Your exact birthweight is [___|___]. [__] jin

(Please notice that the unit of the weight is kilogram, and 1 decimal digit is reserved. Please fill in 00.0 if you are not clear about your exact birthweight.)

A10. Have you ever been seriously ill before you went to elementary school?

1. Yes, I have.   2. No, I haven't.   3. Not Clear.

[1] Hukou refers to a record of household registration system in China that identifies a person as a resident of an area.

A11. Which one of the following best describes the financial conditions of your family before you went to elementary school?

1. Very poor   2. Somewhat poor   3. Moderate
4. Somewhat rich   5. Very rich   6. Not clear

A12. How much do you agree with each of the following statements about your experiences in GRADE 6?

	Strongly disagree	Somewhat disagree	Somewhat agree	Strongly agree
I would try my best to go to school even if I was not feeling very well or I had other reasons to stay at home.	1	2	3	4
I would try my best to finish even the homework I dislike.	1	2	3	4
I would try my best to finish my homework, even if it would take me quite a long time.	1	2	3	4
I was able to express myself clearly.	1	2	3	4
I was able to give quick responses.	1	2	3	4
I was a fast learner.	1	2	3	4
I was curious about new stuff.	1	2	3	4

A13. Your current height is: [__|__|__] centimeters.

A14. Your current weight is: [__|__|__] jin (Please notice that the unit of the weight is 'kilogram' rather than 'jin')

A15. Are you short-sighted?

1. Yes, I am.
   The strength of my left eyeglass is [__|__|__] degrees[2].
   The strength of my right eyeglass is [__|__|__] degrees.
2. Yes, I am. But I am not clear about the strength of my glasses/lens.
3. No, I am not.

A16. Have you been hospitalized over the last year?

1. Yes, I have.   2. No, I haven't.

A17. Which one of the following best describes your general health condition at present?

1. Very poor   2. Not very good   3. Moderate   4. Good   5. Very good

A18. Do you have the feelings below in the last seven days?

---

[2] Degree is the unit of measurement of optical power of lens or glasses used in China, 100 degrees of short-sightedness = -1dioptre.

	**Never**	**Seldom**	**Sometimes**	**Often**	**Always**
Feeling blue	1	2	3	4	5
Depressed	1	2	3	4	5
Unhappy	1	2	3	4	5
Not enjoying life	1	2	3	4	5
Sad	1	2	3	4	5

(Please continue to finish Part B.)

Part B: Family Background

B1. Are you the only child of your family?
1. Yes, I am. **(SKIP TO B4)**
2. No, I am not.

B2. How many FULL OR HALF siblings do you have? (Please fill in 0 if you don't have any sibling.)
[___] elder brother(s)
[___] younger brother(s)
[___] elder sister(s)
[___] younger sister(s)

B3. If the Hukou of your family is not in the local county/district, please answer: do you have any sibling(s) attending schools located in your hometown?
1. The Hukou of my family is in the local county/district.
2. No, I don't have any.
3. Yes, I do. And the eldest one is in grade [___] at present.

B4. Which of the following people live in the same household with you at present? (Please mark all that apply.)
1. Mother
2. Father
3. Full or half sibling(s)
4. Grandparent(s) on mother's/father's side
5. Other relative(s)
6. Other non-relative(s)

B5. Which of the following immediate relatives of yours do not live in the same household with you at present? (Please mark all that apply.)
1. Mother
2. Father
3. Full or half sibling(s)
4. All my immediate relatives (if there is any) live with me.

B6. What is the highest education level your mother has completed?
1. None
2. Finished elementary school
3. Junior high school degree
4. Technical secondary school or technical school degree
5. Vocational high school degree
6. Senior high school degree
7. Junior college degree
8. Bachelor degree
9. Master degree or higher

B7. What is the highest education level your father has completed?
1. None
2. Finished elementary school
3. Junior high school degree
4. Technical secondary school or technical school degree
5. Vocational high school degree
6. Senior high school degree
7. Junior college degree
8. Bachelor degree
9. Master degree or higher

B8. What are the occupations of your parents?

	Mother	Father
Government official, staff of public institutions, civil servant	1	1
Middle/Senior management personnel of enterprises/corporations	2	2
Teacher, engineer, doctor, lawyer	3	3
Technical worker (including driver)	4	4
Ordinary staff or worker in production or manufacturing industry	5	5
Ordinary staff or worker in business or service industry	6	6
Self-employed worker	7	7
Peasant	8	8
Unemployed or laid-off worker	9	9
Other	(Please specify:________)	(Please specify:________)

B9. Which one of the following best describes the financial conditions of your family at present?

1. Very poor 2. Somewhat poor 3. Moderate 4. Somewhat rich 5. Very rich

B10. Do you agree with the following statements?

	No, I don't agree	Yes, I agree
My father often gets drunk.	1	2
My parents quarrel a lot.	1	2
My parents get along very well.	1	2

B11. Do you have a writing desk of your own at home?

1. Yes, I do. 2. No, I don't.

B12. How many books do your family own? (not including textbooks or magazines)

1. Very few 2. Not many 3. Some
4. Quite a few 5. A great number

B13. Do your family own a computer and have an access to the Internet?

0. No, we don't.
1. We have a computer but no access to the Internet.
2. Yes, we have both.

B14. How much time did you spend on homework assigned by your teachers at school last week? (Please fill in 00 hours and 00 minutes if no time is spent on the homework.)

[__|__] hours [__|__] minutes (on average) from Monday to Friday
[__|__] hours [__|__] minutes (on average) at weekends

B15. How much time ON AVERAGE did you spend on the following extra-curricular activities from Monday to Friday last week? (Please fill in 00 hours 00 minutes if no time is spent on these activities.)

	**From Monday to Friday (on average per day)**
Doing homework assigned by parents or cram school	[__|__] hours [__|__] minutes
Taking cram school courses (related to schoolwork)	[__|__] hours [__|__] minutes
Playing sports	[__|__] hours [__|__] minutes
Reading (not including textbooks)	[__|__] hours [__|__] minutes
Watching TV	[__|__] hours [__|__] minutes
Surfing on the Internet or playing video games	[__|__] hours [__|__] minutes
Helping your parents with housework	[__|__] hours [__|__] minutes

B16. How much time ON AVERAGE did you spend on the following extra-curricular activities LAST WEEKEND? (Please fill in 00 hours 00 minutes if no time is spent on these activities)

	**At weekends (on average per day)**
Doing homework assigned by parents or cram school	[__|__] hours [__|__] minutes
Taking cram school courses (related to schoolwork)	[__|__] hours [__|__] minutes
Playing sports	[__|__] hours [__|__] minutes
Reading (not including textbooks)	[__|__] hours [__|__] minutes
Watching TV	[__|__] hours [__|__] minutes
Surfing on the Internet or playing video games	[__|__] hours [__|__] minutes
Helping your parents with housework	[__|__] hours [__|__] minutes

B17. How often do you do the following either alone or with your schoolmates?

	**Never**	**Once a year**	**Once every half year**	**Once a month**	**More than once a month**
Visiting museums, zoos, science museums, etc.	1	2	3	4	5
Going out to watch movies, shows, sports games, etc.	1	2	3	4	5

B18. How much time on average do you sleep every night?

[__|__] hours [__|__] minutes

B19. What kind of extra-curricular courses do you take? (Please mark all that apply.)

0. None
1. Mathematical Olympiad
2. Ordinary Mathematics (not including Mathematical Olympiad)
3. Chinese/Chinese Composition Writing
4. English
5. Painting or drawing
6. Calligraphy
7. Music/Musical instrument
8. Dancing
9. Chess
10. Sports
11. Other (Please specify: __________)

B20. What hobbies do you have? (Please mark all that apply.)

0. None
1. Playing musical instruments (Please specify: __________)
2. Vocal practices/Singing/Dancing/Acting
3. Calligraphy
4. Painting or drawing/Animation
5. Chess (Please specify: __________)
6. Sports (Please specify: __________)
7. Other (Please specify: __________)

B21. What do you usually do in winter and summer vacations? (Please mark all that apply.)

1. Taking cram school courses (related to schoolwork)
2. Attending summer/winter camps
3. Helping parents out
4. Going back to hometown
5. Staying at home
6. Other (Please specify: __________)

B22. How often did your parents do the following to check up on your study last week?

	Never	One or two days	Three or four days	Almost everyday
Checking up on your homework	1	2	3	4
Giving instruction on your homework	1	2	3	4

B23. Do your parents care and are they strict with you about the following?

	They don't care.	They do care about it, but are not strict.	They are very strict about it.
Your homework and examination	1	2	3

Your behavior at school	1	2	3
Attendances at school everyday	1	2	3
Time when you get home everyday	1	2	3
Whom you make friends with	1	2	3
Your dress style	1	2	3
Time you spend on the Internet	1	2	3
Time you spend on watching TV	1	2	3

B24. How often do your parents discuss the following with you?

	**Your mother**			**Your father**		
	**Never**	**Sometimes**	**Often**	**Never**	**Sometimes**	**Often**
Things happened at school	1	2	3	1	2	3
The relationship between you and your friends	1	2	3	1	2	3
The relationship between you and your teachers	1	2	3	1	2	3
Your feelings	1	2	3	1	2	3
Your worries and troubles	1	2	3	1	2	3

B25. How is the general relationship between you and your parents?

	**Not close**	**Not too close nor too far**	**Very close**
Relationship between you and your mother	1	2	3
Relationship between you and your father	1	2	3

B26.Who will be the first one for you to turn to in the following situations?

	**Schoolmates or good friends**	**Parents**	**A relative of yours**	**Teachers**	**No one**
When you want to chat with someone	1	2	3	4	5
When you are in trouble	1	2	3	4	5
When you need help	1	2	3	4	5

B27.What language do you usually speak with your parents?

1. Mandarin Chinese
2. Sometimes dialect of my hometown, sometimes mandarin Chinese
3. Dialect of my hometown
4. Other (Please specify: __________)

B28. How often do you do the following with your parents?

	Never	Once a year	Once every half year	Once a month	Once a week	More than once a week
Having dinner	1	2	3	4	5	6
Reading	1	2	3	4	5	6
Watching TV	1	2	3	4	5	6
Playing sports	1	2	3	4	5	6
Visiting museums, zoos, science museums, etc.	1	2	3	4	5	6
Going out to watch movies, shows, sports games, etc.	1	2	3	4	5	6

B29. How much do you think your parents have done for you?

1. Very little
2. Not very much
3. Not too much nor too little
4. A fair amount
5. A great deal

B30. What is your parents' requirement on your academic record?

1. Being one of the top five of your class
2. Above the average
3. About the average
4. No special requirement

B31. What is the highest level of education your parents expect you to receive?

1. Drop out now
2. Graduate from junior high school
3. Go to technical secondary school or technical school
4. Go to vocational high school
5. Go to senior high school
6. Graduate from junior college
7. Get a bachelor degree
8. Get a Master degree
9. Get a Doctor degree
10. They don’t care

B32. How do you feel about such expectation?

1. Not stressed at all
2. Not very stressed
3. Normal
4. Somewhat stressed
5. Very stressed

B33. What kind of job do your parents most expect you to do in the future?

1. Government official, staff of public institutions, civil servant
2. Manager or administrator of enterprises/corporations
3. Scientist/engineer
4. Teacher/doctor/lawyer
5. Designer
6. Artistic performer/actor/host
7. Professional athlete
8. Technical worker (including driver)
9. Other (Please specify: __________)
10. They don’t care
11. Not clear

B34. Where do you think your parents most expect you to live and work in the future?

1. In rural area
2. In small or medium cities
3. In big cities, such as Beijing/Shanghai/Guangzhou
4. Abroad
5. They don’t care
6. Not clear

B35. Are your parents confident about your future?

1. Not confident at all
2. Not so confident
3. Somewhat confident
4. Very confident

(Please continue to finish Part C)

Part C: School Experiences and Activities

C1. Have you ever gone to a kindergarten/pre-school since you were three years old?
1. Yes, since [__] years old. 2. No, I haven't.

C2. When did you start to go to elementary school?
At [__] years old

C3. From grade 1 to grade 6, how many times have you skipped a grade?
[__] times (Please fill in 0 if you have never skipped a grade.)

C4. From grade 1 to grade 6, how many times have you repeated a grade?
[__] times (Please fill in 0 if you have never repeated a grade.)

C5. From grade 1 to grade 6, how many times have you suspended your schooling? (Suspension of schooling refers to at least one month of absence from school)
[__] times (Please fill in 0 if you have never suspended your schooling.)

C6. From grade 1 to grade 6, how many times have you transferred to a different school?
[__] times (Please fill in 0 if you have never transferred to a different school.)

C7. Have you ever taken English courses in your elementary school?
1. Yes, since grade [___] 2. No, I haven't.

C8. How did your academic record rank in your class when you were in grade 6?
I ranked about [___|___] in my class.

C9. Did you take an entrance examination/placement test when you entered your current school?
1. Yes, I did. 2. No, there's no such test.

C10. Think back when you were in GRADE 6, were the following courses difficult for you?

	Very difficult	A bit difficult	Not very difficult	Not difficult at all	I did not take this course in Grade 6.
Mathematics	1	2	3	4	5
Chinese	1	2	3	4	5
English	1	2	3	4	5

C11. AT PRESENT, are the following courses difficult for you?

	Very difficult	A bit difficult	Not very difficult	Not difficult at all
Mathematics	1	2	3	4
Chinese	1	2	3	4
English	1	2	3	4

C12. How does your academic record rank in your class at present?

1. Near the bottom 2. Below the average 3. About the average
4. Above the average 5. Around the top

C13. How much do you agree with each of the following statements about the main subjects?

	Strongly disagree	Somewhat disagree	Somewhat agree	Strongly agree
Mathematics helps a lot with my future development.	1	2	3	4
Chinese helps a lot with my future development.	1	2	3	4
English helps a lot with my future development.	1	2	3	4
My mathematics teacher always asks me to answer questions in class.	1	2	3	4
My Chinese teacher always asks me to answer questions in class.	1	2	3	4
My English teacher always asks me to answer questions in class.	1	2	3	4
My mathematics teacher always praises me.	1	2	3	4
My Chinese teacher always praises me.	1	2	3	4
My English teacher always praises me.	1	2	3	4

C14. Do you agree that boys are better at mathematics than girls?

1. Yes, I do. 2. No, I don't.

C15. Do your parents agree that boys are better at mathematics than girls?

1. Yes, they do. 2. No, they don't.

C16. Do most people around you agree that boys are better at mathematics than girls?

1. Yes, they do. 2. No, they don't.

C17. How much do you agree with each of the following statements about your school life?

	Strongly disagree	Somewhat disagree	Somewhat agree	Strongly agree
I am always late for class.	1	2	3	4
I always skip classes.	1	2	3	4
My parents always receive criticism on me from my teacher.	1	2	3	4
My homeroom teacher always praises me.	1	2	3	4

My homeroom teacher always criticizes me.	1	2	3	4
Most of my classmates are nice to me.	1	2	3	4
I think I am easy to get along with.	1	2	3	4
My class is in good atmosphere.	1	2	3	4
I often take part in school/class activities.	1	2	3	4
I feel close to people in this school.	1	2	3	4
I feel bored in this school.	1	2	3	4
I hope that I could transfer to another school.	1	2	3	4

C18. Do you live in school at night from Monday to Thursday?
1. Yes, I do.   2. No, I don't.

C19. How many best friends do you have?
[__|__] friends

C20. Please write down NAMES of 5 of your best friends truthfully in the first column of the table below, and then fill in the blanks with number that best describes their conditions. (Please write down as many best friends as you have if there are less than 5.)

**Names of 5 of your best friends**	**Sex**<br>**1. Male**<br>**2. Female**	**Location of Hukou**<br>**1. In the local county/district**<br>**2. Not in the local county/district**	**Does he/she attend the same school with you?**<br>**1. Yes.**<br>**2. No.**	**Is he/she in the same class with you?**<br>**1. Yes.**<br>**2. No.**
1____________	[____]	[____]	[____]	[____]
2____________	[____]	[____]	[____]	[____]
3____________	[____]	[____]	[____]	[____]
4____________	[____]	[____]	[____]	[____]
5____________	[____]	[____]	[____]	[____]

C21. How many of your best friends mentioned above fit in the following descriptions?

	**None of them**	**One or two of them**	**Most of them**
Doing well in academic performance	1	2	3
Studying hard	1	2	3
Expecting to go to college	1	2	3

Skipping classes	1	2	3
Criticized or punished for violating school rules	1	2	3
Always fighting with others	1	2	3
Smoking or drinking alcohol	1	2	3
Always going to net bars or video arcade	1	2	3
Having had or is having a romance	1	2	3
Dropped out of school	1	2	3

C22. What is the highest level of education you expect yourself to receive?

1. Drop out now
2. Graduate from junior high school
3. Go to technical secondary school or technical school
4. Go to vocational high school
5. Go to senior high school
6. Graduate from junior college
7. Get a bachelor degree
8. Get a Master degree
9. Get a Doctor degree
10. I don't care

C23. What kind of job do you most expect to do in the future?

1. Government official, staff of public institutions, civil servant
2. Manager or administrator of enterprises/corporations
3. Scientist/engineer
4. Teacher/doctor/lawyer
5. Designer
6. Artistic performer/actor/host
7. Professional athlete
8. Technical worker (including driver)
9. Other (Please specify: __________)
10. I don't care

C24. Where do you most expect to live and work when you grow up?

1. In rural area
2. In medium or small cities
3. In big cities, such as Beijing/Shanghai/Guangzhou
4. Abroad
5. I don't care

C25. Are you confident in your future?

1. Not confident at all
2. Not so confident
3. Somewhat confident
4. Very confident

C26. Do you think your classmates from the local county/district will do the following to those who are not from the local county/district? (Please give your answers based on your assumption if you don't have any classmate from non-local counties/districts.)

	**Will they do this to students from other *urban* counties/districts?**<br>**1. Yes, they will.**<br>**2. No, they won't.**	**Will they do this to students from other *rural* counties/districts?**<br>**1. Yes, they will.**<br>**2. No, they won't.**
Making complaints or offensive remarks about them	[__]	[__]
Playing together with them	[__]	[__]
Discussing questions related to schoolwork	[__]	[__]
Making friends with them	[__]	[__]

C27. Have you ever transferred to a different school since grade 7?

1. Yes, I have. 2. No, I haven't. **(SKIP TO C29)**

C28. Please fill in the blanks the types of junior high schools you have attended previously (NOT INCLUDING YOUR CURRENT SCHOOL):

	**Types of school**<br>**1. Public school**<br>**2. Privately-run and state-subsidized school [See Note]**<br>**3. Privately-run school**<br>**4. School for migrant children**	**Is this school in the same county/district as your current school?**<br>**1. Yes, it is.**<br>**2. No, it is not.**
Fall semester of Grade 7	[__]	[__]
Spring semester of Grade 7	[__]	[__]
Fall semester of Grade 8	[__]	[__]
Spring semester of Grade 8	[__]	[__]

Note: Privately-run and state-subsidized schools are those run by civilians where students' tuitions are paid by the government.

C29. What kind of advice do you think your homeroom teacher will give to you about your education after junior high school?

1. Going to a key senior high school
2. Going to an ordinary senior high school
3. Going to a vocational high school
4. Going to a technical secondary school/technical school
5. Not going any further after graduating from junior high school
6. None

C30. In grade 9, what kind of preparation will be made in your class for the coming senior high school entrance examination? (Please mark all that apply.)

0. None
1. Finishing school courses ahead of schedule to have more time for a general review
2. Arranging a number of exams
3. Arranging after-class tutoring

Please continue to answer question C31-C33 in the box below if you are not from the local country/district. Please SKIP TO PART D if you are from the local county/district.

C31. According to the local policy, are you allowed to apply for a senior high school in this city (prefecture-level city)?

1. I am allowed to apply for a key senior high school.
2. I am only allowed to apply for an ordinary senior high school but not a key one.
3. I am not allowed to apply for either an ordinary senior high school or a key one.
4. Not clear.

C32. What do you expect to do after graduating from junior high school?

1. Going to a senior high school in this city (prefecture-level city)
2. Going to a senior high school back in my hometown
3. Going to a senior high school in other places
4. Going to a vocational high school/technical secondary school/technical school in this city
5. Going to a vocational high school/technical secondary school/technical school in other places
6. Getting a job
7. Other (Please specify: __________)

C33. Among the people around you, do you know anyone elder than you who had left his/her hometown since very young like you and have been successfully enrolled in a college?

1. Hardly any   2. Very few   3. Some
4. Quite a few   5. A great number

(Please continue to finish Part D)

Part D: Contact Information

We hope to continue the tracking of your growth and improvement in the next year by follow-up surveys. Please leave the contact information of yours and that of your parents, so that we could keep in touch with you:

D1. Your contact information:
Your QQ number: _______________
Your mobile phone number: _________________

D2. Your family's telephone number:_________________

D3. Your father's contact information:
Name: _______________ Mobile phone number: _________________

D4. Your mother's contact information:
Name: _______________ Mobile phone number: _________________

This is the end of the questionnaire. Thank you for your engagement and cooperation! We wish you a happy life and a successful future!

Class: __________ Student Name: _____________

Name of Homeroom Teacher: ___________ Name of School: ____________

Location of School: County (District):___________ City: ____________

Province (Autonomous Region/Municipality):____________

China Education Panel Survey

Academic Year 2013-2014

Parent Questionnaire for Grade 7

Parents answering this questionnaire do not need to fill in the blanks in the box below.

1. Code of Questionnaire: [____|____|____|____|____|____]
2. Code of County/District: [____|____|____|____|____|____]
3. Verifier: ______________________

Hi Dear Parents,

China Education Panel Survey is the first large-scale, nationwide, and longitudinal social survey of junior high students in China. The purpose of CEPS is to provide detailed and reliable data for the study of the current situation and future development of China's primary education.

CEPS is jointly conducted by the National Survey Research Center (NSRC) at Renmin University of China and academic institutions throughout the country. During the academic year 2013-2014, we are going to survey nearly 20,000 students as well as their parents, homeroom teachers, subject teachers and school administrators across the country. Through scientific sampling, your child and you have been chosen as subjects of this survey. Therefore, we are asking you here to complete this questionnaire and we are very grateful to your understanding, support and help!

There are neither right nor wrong answers to these questions and your answers will not be used to evaluate your child and you. Therefore, please answer all the questions truthfully based on your own ideas and experiences. We pledge here that under the *Statistics Law of the People's Republic of China*, we will hold all the information you are going to provide in strict confidence and it will never be given away to any particular individual or institution. Thank you for your cooperation.

When answering a multiple-choice question, please circle the number of your choice; when answering a blank-filling question, please write down words or numbers on the lines in the square brackets.

National Survey Research Center (NSRC), Renmin University of China

April, 2014

Part A: Family Education

A1. What is your relationship to this child?

1. Biological father
2. Biological mother
3. Stepfather
4. Stepmother
5. Grandfather on mother's/father's side
6. Grandmother on mother's/father's side
7. Other relative male
8. Other relative female
9. Other non-relative male
10. Other non-relative female

A2. Does this child take any cram school or extra-curricular course this semester?

1. Yes, he/she does.
2. No, he/she doesn’t.

A3. What is the total cost of his/her cram school or extra-curricular courses this semester?

[__|__|__|__|__] Yuan (Please fill in 00000 if there is no cost.)

A4. Did the family members help this child with his/her homework last week?

1. There is no need
2. Need but no one helped
3. Yes, one or two days
4. Yes, three or four days
5. Yes, almost every day

A5. To what extent could you meet the requirement this semester if the teachers require the parents to check their child’s homework, such as writing “checked”, signing names, etc.?

0. The teachers do not have such requirement
1. Completely
2. Mostly
3. Rarely
4. Not at all

A6. How much pocket money does this child receive on average EVERY WEEK?

[___|___|___] Yuan (Please fill in 000 if there is no cost.)

A7. How is this child’s New Year money (money given to children as a lunar New Year gift) usually dealt?

1. Kept by parents
2. Kept by this child, and disposed as parents suggested
3. Kept and disposed by the child
4. This child never received New Year money

A8. Do you care and are you strict with this child about the following?

	I don’t care.	I do care about it, but am not strict.	I’m very strict about it.
His/her homework and examination	1	2	3
His/her behavior at school	1	2	3
His/her Attendances at school everyday	1	2	3

Time when he/she get home everyday	1	2	3
Whom he/she makes friends with	1	2	3
His/her dress style	1	2	3
Time he/she spends on the Internet	1	2	3
Time he/she spends on watching TV	1	2	3

A9. What do you usually do when this child and you have different opinions?

1. Follow him/her mostly
2. Persuade him/her to agree with me
3. Force him/her to agree with me
4. Follow the one who is right after discussion
5. Leave it over without a conclusion
6. Other (Please specify: ____________)

A10. How many friends does this child have at school?

0. None **(SKIP TO A13)**
1. About 1-5 friends
2. About 6-10 friends
3. More than 10 friends
4. Not clear

A11. Do you know the friends who often play together with this child?

0. No, I don't. **(SKIP TO A13)**
1. Yes, I know some of them.
2. Yes, I know all of them.

A12. Do you know the parents of the friends who often play together with this child?

0. No, I don't.
1. Yes, I know some of them.
2. Yes, I know all of them.

A13. How much time do you spend directly on this child (including taking care of his/her living, helping with his/her coursework, and entertainment) EVERY DAY ON AVERAGE?

[___|___] hours every day on average

A14. How often do you discuss the following with this child?

	**Never**	**Sometimes**	**Often**
Things happened at school	1	2	3
The relationship between he/she and his/her friends	1	2	3
The relationship between he/she and his/her teachers	1	2	3
his/her feelings	1	2	3
his/her worries and troubles	1	2	3

A15. When you are at home with this child, what language does he/she usually speak?

1. Mandarin Chinese
2. Sometimes dialect of his/her hometown, sometimes mandarin Chinese
3. Dialect of his/her hometown
4. Other

A16. When you are at home with this child, what language do you usually speak?

1. Mandarin Chinese
2. Sometimes dialect of my hometown, sometimes mandarin Chinese
3. Dialect of my hometown
4. Other

A17. How often did the family members do the following together with this child over the last year?

	**Never**	**Once a Year**	**Once every half year**	**Once a Month**	**Once a Week**	**More Than Once a Week**
Having dinner	1	2	3	4	5	6
Reading	1	2	3	4	5	6
Watching TV	1	2	3	4	5	6
Playing sports	1	2	3	4	5	6
Visiting museums, zoos, science museums, etc.	1	2	3	4	5	6
Going out to watch movies, shows, sports games, etc.	1	2	3	4	5	6

A18. What is the highest level of education do you expect this child to receive?

1. Drop out now
2. Graduate from junior high school
3. Go to technical secondary school or technical school
4. Go to vocational high school
5. Go to senior high school
6. Graduate from junior college
7. Get a bachelor degree
8. Get a Master degree
9. Get a Doctor degree

A19. What kind of job do you most expect this child to do in the future?

1. Government official, staff of public institutions, civil servant
2. Manager or administrator of enterprises/corporations
3. Scientist/engineer
4. Teacher/doctor/lawyer
5. Designer
6. Artistic performer/actor/host
7. Professional athlete
8. Technical worker (including driver)
9. Other (Please specify______________)
10. I don't care

A20. Where do you most expect this child to live and work in the future?

1. In rural area
2. In small or medium cities
3. In big cities, such as Beijing/Shanghai/Guangzhou
4. Abroad
5. I don't care

A21. Are you confident in the future of this child?

1. Not confident at all
2. Not so confident
3. Somewhat confident
4. Very confident

(Please continue to finish Part B.)

Part B: The Relationship between Parents and School

B1. Do this child's parents attend the parent meeting this semester?

1. The school held one, and the parents did attend.
2. The school held one, but the parents didn't attend.
3. Not held yet, but the parents are going to attend once held
4. Not held yet, and the parents are not going to attend if held

B2. How many times have this child's parents contacted the teacher at school this semester?

1. Never 2. Once 3. Two to four times 4. Five times or more

B3. When contacting the child's teacher this semester, what did the parents usually talk about with the teacher? (Please mark all that apply.)

0. The parents have never contacted the child's teacher this semester.
1. Schoolwork 2. The child's morals 3. Mental health
4. Physical health 5. Friends of the child 6. Other

B4. How many times has this child's teacher contacted the parents this semester?

1. Never 2. Once 3. Two to four times 4. Five times or more

B5. When the teacher contacted you this semester, what did he/she usually talk about with you? (Please mark all that apply.)

0. The teacher has never contacted me this semester.
1. Schoolwork 2. The child's morals 3. Mental health
4. Physical health 5. Friends of the child 6. Other

B6. Are you afraid of communicating with the school teacher?

1. Quite afraid 2. A little bit afraid 3. Not afraid at all

B7. In your opinion, is the teacher responsible for this child?

1. Not responsible at all 2. Not so responsible 3. Moderate
4. Somewhat responsible 5. Very responsible

B8. In your opinion, is the teacher patient to your child?

1. Not patient at all 2. Not so patient 3. Moderate
4. Somewhat patient 5. Very patient

B9. In your opinion, which kind of the following students benefits most from the teaching method of the teacher?

1. Students with academic record below average
2. Students with average academic record
3. Students with academic record above average
4. All kinds of students
5. It's hard to say.

B10. Do you agree that it is totally the teachers' responsibility to educate children?

1. Strongly disagree
2. Somewhat disagree
3. It's hard to say
4. Somewhat agree
5. Strongly agree

B11. Does this child like his/her teachers and schoolmates at school?

	Strongly dislike them	Somewhat dislike them	Somewhat like them	Like them very much
His/her homeroom teacher	1	2	3	4
Other teachers at school	1	2	3	4
His/her schoolmates	1	2	3	4

B12. Which of the following factors do you think have effects on students' grade? (Please mark all that apply.)

1. Parents' discipline
2. Teachers' teaching
3. Class
4. Friends
5. Attendance of cram school
6. Extent of hardworking
7. Talent and capability

B13. Do you agree that boys are better at mathematics than girls?

1. Yes, I do.
2. No, I don't.

B14. Do most people around you agree that boys are better at mathematics than girls?

1. Yes, they do.
2. No, they don't.

If this child has schoolmates from non-local county/district, please answer question B15-B20 according to the real condition; if this child doesn't have schoolmates from non-local county/district, please answer these questions according to your assumption:

B15. What kind of effect do you think will the increase of students from non-local county/district have on the educational quality of the school?

1. Harmful
2. Beneficial
3. No effect

B16. What kind of effect do you think will the increase of students from non-local county/district have on the atmosphere of the school?

1. Harmful 2. Beneficial 3. No effect

B17. Which kind of students do you think will the school teachers like better?

1. Students from local county/district
2. Students from non-local county/district
3. There is no difference

B18. When students from non-local county/district have conflicts with students from local county/district, whom do you think will the school teachers be partial to?

1. Students from local county/district
2. Students from non-local county/district
3. The teacher won't be partial to anyone

B19. Do you think the school teachers are prejudiced against students from non-local county/district?

1. Not prejudiced at all 2. A little bit prejudiced
3. Somewhat prejudiced 4. Very prejudiced

B20. Do you think the school teachers are prejudiced against the parents of students from non-local county/district?

1. Not prejudiced at all 2. A little bit prejudiced
3. Somewhat prejudiced 4. Very prejudiced

(Please continue to finish Part C.)

Part C: School Education

C1. From whom/which of the following did your family get to know the application and enrollment procedures of this school? (Please mark all that apply.)

1. The primary school that this child graduated from
2. The Sub-district office/township government
3. The Internet
4. Information provided by this school
5. Parents of other students
6. Other (Please specify: _____________)

C2. Which of the following did your family do in order to enroll this child in this school? (Please mark all that apply.)

1. Asking friends for help
2. Giving presents to the related government/school leaders
3. Paying extra fees (Please specify: __________)
4. Buying a house/apartment in the "education district" where this school is located
5. Changing the location of the Hukou of your family
6. Transferring the Hukou of your family or this child under other relatives or friends
7. Letting this child take all kinds of achievement tests
8. Other (Please specify: _____________)
9. We did none of the above.

C3. When enrolling this child in this school, which of the following certificates did you prepare? (Please mark all that apply.)

1. The booklet of Hukou (the official book of registered permanent residence)
2. The property ownership certificate or lease contract of your house/apartment
3. (Temporary) residential permission
4. Social security for at least one year
5. The family planning certificate
6. Business License or employment certificate
7. "Scores" meeting some requirement
8. Other (Please specify :______________)

C4. Is it difficult for your family to obtain the above certificates?

0. No, it isn't.
1. Yes, it is. And among the above certificates:

The most difficult to obtain is [__].

The second difficult to obtain is [__].

The third difficult to obtain is [__].

C5. What is the total fee paid to this school EVERY semester?

	**Fees**
The total fee paid to this school:	[__|__|__|__|__] Yuan<br>(Please fill in 00000 if it is free.)
Including: Tuition fees	[__|__|__|__|__] Yuan<br>(Please fill in 00000 if it is free.)
Book fees	[__|__|__|__|__] Yuan<br>(Please fill in 00000 if it is free.)
Activity fees	[__|__|__|__|__] Yuan<br>(Please fill in 00000 if it is free.)
Meal expenses	[__|__|__|__|__] Yuan<br>(Please fill in 00000 if it is free.)
Dormitory fees	[__|__|__|__|__] Yuan<br>(Please fill in 00000 if it is free, or fill in 99999 if this child does not live in the dorm)
Other fees (Please specify: ______)	[__|__|__|__|__] Yuan<br>(Please fill in 00000 if it is free.)

C6. Does your child receive the governmental subsidies in education this semester? (Please mark all that apply.)

1. Yes, his/her book fees are exempted
2. Yes, the school provided free lunch
3. Yes, he/she received the grant aid for students ([__|__|__|__] Yuan/semester)
4. Other (Please specify: ___________________)
5. Not receiving any governmental subsidies

C7. How many students are there in this child's class?

[__|__|__] students

C8. Do you think the size of this child's class is too small or too big?

1. Too small 2. Somewhat small 3. Moderate
4. Somewhat big 5. Too big

C9. What do you think of the general attitude of this child towards schoolwork?

1. Not serious at all 2. Somewhat not serious 3. Moderate

4. Somewhat serious 5. Very Serious

C10. How does this child's academic record rank in his/her class at present?

1. Near the bottom 2. Below the average 3. About the average
4. Above the average 5. Around the top

C11. What is your requirement on this child's academic record?

1. Being one of the top five of his/her class 2. Above the average
3. About the average 4. No special requirement

If you are the parents from non-local county/district, please continue answering question C12-C14 in the following box. If you are the parents from local county/district, please skip to Part D.

C12. According to the local policy, is this child allowed to apply for a senior high school in this city (prefecture-level city)?

1. He/she is allowed to apply for a key senior high school.
2. He/she is allowed to apply for an ordinary senior high school, but not a key one.
3. He/she is not allowed to apply for either an ordinary senior high school or a key one. **(SKIP TO C14)**
4. Not clear **(SKIP TO C14)**

C13. Are there any special requirements that this child needs to meet in order to apply for an ordinary senior high school in this city (prefecture-level city)? (Please mark all that apply.)

0. There are no special requirements
1. Studying in the local (prefecture-level city) junior high school for three years
2. Local Hukou of this city (prefecture-level city) or enough “score” to obtain it
3. Local Hukou of this province
4. The property ownership certificate or lease contract of parents’ house/apartment
5. (Temporary) residential permission
6. Social security for at least one year
7. Family planning certificate
8. Business license or employment certificate
9. Other (Please specify :______________)

C14. What is your plan for this child after he/she graduates from junior high school?

1. To study in a senior high school in this city (prefecture-level city)
2. To study in a senior high school in his/her hometown
3. To study in a senior high school in another place
4. To study in a technical secondary school/ vocational high school in this city
5. To study in a technical secondary school/ vocational high school in this another place
6. To get a job
7. Other (Please specify: ________________)

(Please continue to finish Part D.)

Part D: Basic Information of This Child

D1. The birthplace of this child is:

1. In the local county/district
2. Not in the local county/district:

    County (District): [_________], City: [_________],

    Province (Autonomous Region/Municipality): [_________]

D2. The location of his/her Hukou[1] WHEN BORN is:

1. In the local county/district
2. Not in the local county/district:

    County (District): [_________], City: [_________],

    Province (Autonomous Region/Municipality): [_________]

D3. The location of his/her Hukou AT PRESENT is:

1. In the local county/district
2. Not in the local county/district:

    County (District): [_________], City: [_________],

    Province (Autonomous Region/Municipality): [_________]

D4. What is the type of his/her Hukou WHEN BORN? (Agricultural Hukou refers to rural Hukou, a record that identifies a person as a rural resident. Non-agricultural Hukou refers to urban Hukou. Residential Hukou is a general record of residence assigned to all residents of a certain region, regardless of rural or urban background)

1. Agricultural Hukou 2. Non-agricultural Hukou

3. Residential Hukou 4. Other

D5. What is the type of his/her Hukou AT PRESENT?

1. Agricultural Hukou **(SKIP TO D7)** 2. Non-agricultural Hukou

3. Residential Hukou 4. Other **(SKIP TO D7)**

D6. In which year did this child obtain the non-agricultural Hukou?

In the year of [__|__|__|__]

(Please fill in 9997 if this child obtained the non-agricultural Hukou since the beginning.)

D7. In which year did this child come to live in this county/district?

[1] Hukou refers to a record of household registration system in China that identifies a person as a resident of an area.

In the year of [__|__|__|__]

(Please fill in 9997 if this child has been living in this county/district since born.)

D8. Who was in charge of taking care of this child before he/she went to primary school?

1. His/her parents
2. His/her grandparents on mother's side
3. His/her grandparents on father's side
4. A Nanny
5. Other relatives of this child
6. Other non-relatives (not including the nanny)

D9. Which one of the following best describes the financial conditions of his/her family before this child went to primary school?

1. Very poor
2. Somewhat poor
3. Moderate
4. Somewhat rich
5. Very rich

D10. Has this child ever been seriously ill before he/she went to primary school?

1. Yes, he/she has.
2. No, he/she hasn’t.

D11. What is the birthweight of this child?

[__|__]. [__] jin

(Please notice that the unit of the weight is kilogram, and 1 decimal digit is reserved. Please fill in 00.0 if you are not clear about your exact birthweight.)

D12. What is the current height and weight of this child?

Height: [___|___|___] centimeters

Weight: [___|___|___] jin

(Please notice that the unit of the height and weight is centimeter and jin, respectively.)

D13. Which one of the following best describes the general health condition of this child at present?

1. Very poor
2. Not very good
3. Moderate
4. Good
5. Very good

D14. Is this child short-sighted at present?

1. Yes. he/she is

   The strength of his/her left eyeglass is [__|__|__] degrees[2].

   The strength of his/her right eyeglass is [__|__|__] degrees.

2. No. he/she isn’t

[2] Degree is the unit of measurement of optical power of lens or glasses used in China, 100 degrees of short-sightedness = -1dioptre.

D15. Does your child have the following disorders at present? (Please mark all that apply.)

0. None
1. Vision disorders (not including short-sighted)
2. Hearing disorders
3. Extremity Disabled
4. Language Disorders
5. Autism or other mental disorders
6. Attention deficit hyperactivity disorder (ADHD)
7. Other (Please specify: _____________)

D16. Which of the following medical insurance does this child have at present? (Please mark all that apply.)

1. Reimburse for child's medical expenses by parents' work units
(The proportion of the reimbursement of the total medical expenses is [__|__|__]%.)
2. The basic medical insurance for urban residents
3. The new rural cooperative medical insurance
4. The medical insurance for children
5. Commercial medical insurance
6. Other medical insurance (Please specify: _____________________)
7. This child doesn't have any medical insurance at present.

D17. Does this child fit in the following descriptions?

	Not fit at all	Somewhat not fit	Somewhat fit	Exactly fit
Able to express himself/ herself clearly	1	2	3	4
Able to give quick responses	1	2	3	4
A faster learner	1	2	3	4
Curious about new stuff	1	2	3	4

(Please continue to finish Part E.)

Part E: Parents, Family and Community

E1. In which year were you born?

In the year of [___|___|___|___]

E2. Your ethnic nationality is:

1. The Han nationality 2.The Mongol nationality
3.The Manchu nationality 4. The Hui nationality
5.The Tibetan nationality 6.The Zhuang nationality
7. The Uygur nationality 8.Other (Please specify: __________)

E3. Where do you live at present?

1. In the local county/district 2. Not in the local county/district

E4. What is the type of your Hukou at present?

1. Agricultural Hukou **(SKIP TO E6)** 2. Non-agricultural Hukou
3. Residential Hukou 4. Other **(SKIP TO E6)**

E5. In which year did you obtain the non-agricultural Hukou?

In the year of [__|__|__|__]

(Please fill in 9997 if this child obtained the non-agricultural Hukou since the beginning.)

E6. What is your political affiliation?

1. Member of the Communist Party
2. Member of a Democratic Party
3. The masses

E7. What is the highest education level you have completed?

1. None 2. Finished elementary school
3. Junior high school degree 4. Technical secondary school or technical school degree
5. Vocational high school degree 6. Senior high school degree
7. Junior college degree 8. Bachelor degree
9. Master degree or higher

E8. What is your current occupation?

1. Government official, staff of public institutions, civil servant
2. Middle/Senior management personnel of enterprises/corporations

3. Teacher, engineer, doctor, lawyer
4. Technical worker (including driver)
5. Ordinary staff or worker in production or manufacturing industry
6. Ordinary staff or worker in business or service industry
7. Self-employed worker
8. Farmer
9. Unemployed or laid-off worker
10. Other (Please specify: __________)

E9. Which one of the following best describes your general health condition at present, compared with others at your age?

1. Very poor   2. Not very good   3. Moderate
4. Good   5. Very good

E10. How much time do you usually spend on the following every day in your spare time?

	Never	Less than one hour	one to two hours	More than two hours
Reading (including newspapers and magazines)	1	2	3	4
Watching TV	1	2	3	4
Surfing on the Internet	1	2	3	4

E11. How often do you do the following?

	Never	Several Times a Year	Several Times a Month	Several times a Week	Every Day
Drinking alcohol	1	2	3	4	5
Smoking	1	2	3	4	5

E12. Which of the following people live in the same household with this child at present? (Please mark all that apply.)

1. Father of this child
2. Mother of this child
3. Full or half sibling(s) of this child
4. Spouse of this child’s sibling(s)
5. Grandparent(s) of this child on mother's/father's side
6. Other relative(s) of this child
7. Other non-relative(s)

E13. Which of the following immediate relatives of this child do not live in the same household with him/her at present? (Please mark all that apply.)

1. Father of this child
2. Mother of this child
3. Full or half sibling(s) of this child
4. All the immediate relatives (if there is any) of this child live with him/her.

E14. What is the location of your Hukou at present?

1. In the local county/district **(SKIP TO E19)**
2. Not in the local county/district:
   County (District): [_________], City: [_________],
   Province (Autonomous Region/Municipality): [_________]

E15. Does this child have any FULL OR HALF siblings living in the hometown?

1. No, he/she doesn't. **(SKIP TO E19)**
2. Yes, he/she has [___] sibling(s) living in the hometown.

E16. Whom do his/her sibling(s) in the hometown live with?

1. Father/Mother
2. Grandparent(s) on mother's/father's side
3. Other Relative(s)
4. Other

E17. How old is the eldest one among these sibling(s) living in the hometown at present?

[__|__] years old

E18. Is there any one of these sibling(s) attending schools at present?

0. No, there isn't.
1. Yes, the highest grade that they are in is Grade [__|__]

E19. Which one of following best describes the financial conditions of your family at present?

1. Very poor
2. Somewhat poor
3. Moderate
4. Somewhat rich
5. Very rich

E20. Do your family receive subsistence allowance at present?

1. Yes, we do.
2. No, we don't.

E21. Which one of following best describes the income level of your family compared with that of others in your community?

1. Very Low
2. Somewhat low
3. Average
4. Somewhat high
5. Very high

E22. Is there anyone in your family that needs long-term care because of illness or mobility problems?

1. Yes, there is. 2. No, there isn't.

E23. What type of house/apartment do you live in at present?

1. Temporary work shed
2. Basement
3. Rural bungalow
4. Rural building with more than one story
5. Urban bungalow
6. Ordinary urban apartment
7. Urban housing estate
8. Other (Please specify: ____________)

E24. Is the building/house/apartment you are living in also used for production/business?

1. Yes, it is. 2. No, it isn't.

E25. How many rooms are there in the building/house/apartment you are living in? (Including living rooms and bedrooms, but not including kitchens or bathrooms)

[__|__] room(s)

E26. Is there tap water (running water) in your building/house/apartment?

1. Yes, there is. 2. No, there isn't.

E27. Is there a private toilet in your building/house/apartment?

1. Yes, there is. 2. No, there isn't.

E28. Which kind of the following toilets are used by your family at present?

1. Flush Toilet 2. Squat Toilet 3. Cement Cesspit 4. Soil Cesspit

E29. Which kind of area are you living in?

1. Central area of the city/county
2. Outskirts of the city/county
3. The "rural-urban continuum" area of the city/county
4. Towns outside the city/county
5. Rural area
6. Other (Please specify: ____________)

E30. Which kind of community are you living in?

1. Old-style city community without reconstruction
2. Single or compound community affiliated to a work unit (Danwei)
3. Ensuring housing supplied by social secure/subsidy system

4. Ordinary commercial residential building
5. Elite housing
6. Villages inside the city ("Cheng Zhong Cun")
7. Rural Area
8. Other (Please specify: ____________)

E31. Which kind of neighborhood are you living with? (Please mark all that apply.)

1. Local peasants
2. Local labor workers
3. Some are migrant workers
4. Most are migrant workers
5. Teachers/doctors/engineers/civil servants
6. Senior management personnel of enterprises/corporations
7. Other
8. Not clear

E32. Are there any of the following facilities provided in the community you live in? (Please mark all that apply.)

1. Public transportation 2. Kindergarten 3. Hospital 4. Park

E33. Do you agree with the following statements about the community you live in?

	Strongly disagree	Somewhat disagree	Somewhat disagree	Strongly agree
Safe	1	2	3	4
Clean	1	2	3	4
Hardly any pollution	1	2	3	4

Part F: Contact Information

We hope to continue the tracking of the growth and improvement of this child in the next year by follow-up surveys. Please leave the contact information of the parents, so that we could keep in touch with this child and his/her parents:

F1. Telephone number of this child's family: ________________________

F2. The contact information of this child's father

Name: ________________ Mobile phone number: ____________________

F3. The contact information of this child's mother

Name: ________________ Mobile phone number: ____________________

This is the end of the questionnaire. Thank you very much for your support!

We wish you a healthy and happy life!

Class: __________ Student Name: _____________

Name of Homeroom Teacher: ___________ Name of School: ____________

Location of School: County (District):___________ City: ____________

Province (Autonomous Region/Municipality):____________

China Education Panel Survey

Academic Year 2013-2014

Parent Questionnaire for Grade 9

Parents answering this questionnaire do not need to fill in the blanks in the box below.

1. Code of Questionnaire: [____|____|____|____|____|____]
2. Code of County/District: [____|____|____|____|____|____]
3. Verifier: ______________________

Hi Dear Parents,

China Education Panel Survey is the first large-scale, nationwide, and longitudinal social survey of junior high students in China. The purpose of CEPS is to provide detailed and reliable data for the study of the current situation and future development of China's primary education.

CEPS is jointly conducted by the National Survey Research Center (NSRC) at Renmin University of China and academic institutions throughout the country. During the academic year 2013-2014, we are going to survey nearly 20,000 students as well as their parents, homeroom teachers, subject teachers and school administrators across the country. Through scientific sampling, your child and you have been chosen as subjects of this survey. Therefore, we are asking you here to complete this questionnaire and we are very grateful to your understanding, support and help!

There are neither right nor wrong answers to these questions and your answers will not be used to evaluate your child and you. Therefore, please answer all the questions truthfully based on your own ideas and experiences. We pledge here that under the *Statistics Law of the People's Republic of China*, we will hold all the information you are going to provide in strict confidence and it will never be given away to any particular individual or institution. Thank you for your cooperation.

When answering a multiple-choice question, please circle the number of your choice; when answering a blank-filling question, please write down words or numbers on the lines in the square brackets.

National Survey Research Center (NSRC), Renmin University of China

April, 2014

Part A: Family Education

A1. What is your relationship to this child?

1. Biological father
2. Biological mother
3. Stepfather
4. Stepmother
5. Grandfather on mother's/father's side
6. Grandmother on mother's/father's side
7. Other relative male
8. Other relative female
9. Other non-relative male
10. Other non-relative female

A2. Does this child take any cram school or extra-curricular course this semester?

1. Yes, he/she does.
2. No, he/she doesn't.

A3. What is the total cost of his/her cram school or extra-curricular courses this semester?

[__|__|__|__|__] Yuan (Please fill in 00000 if there is no cost.)

A4. Did the family members help this child with his/her homework last week?

1. There is no need
2. Need but no one helped
3. Yes, one or two days
4. Yes, three or four days
5. Yes, almost every day

A5. To what extent could you meet the requirement this semester if the teachers require the parents to check their child's homework, such as writing "checked", signing names, etc.?

0. The teachers do not have such requirement
1. Completely
2. Mostly
3. Rarely
4. Not at all

A6. How much pocket money does this child receive on average EVERY WEEK?

[___|___|___] Yuan (Please fill in 000 if there is no cost.)

A7. How is this child's New Year money (money given to children as a lunar New Year gift) usually dealt?

1. Kept by parents
2. Kept by this child, and disposed as parents suggested
3. Kept and disposed by the child
4. This child never received New Year money

A8. Do you care and are you strict with this child about the following?

	I don't care.	I do care about it, but am not strict.	I'm very strict about it.
His/her homework and examination	1	2	3
His/her behavior at school	1	2	3
His/her Attendances at school everyday	1	2	3

Time when he/she get home everyday	1	2	3
Whom he/she makes friends with	1	2	3
His/her dress style	1	2	3
Time he/she spends on the Internet	1	2	3
Time he/she spends on watching TV	1	2	3

A9. What do you usually do when this child and you have different opinions?

1. Follow him/her mostly
2. Persuade him/her to agree with me
3. Force him/her to agree with me
4. Follow the one who is right after discussion
5. Leave it over without a conclusion
6. Other (Please specify: ____________)

A10. How many friends does this child have at school?

0. None **(SKIP TO A13)**
1. About 1-5 friends
2. About 6-10 friends
3. More than 10 friends
4. Not clear

A11. Do you know the friends who often play together with this child?

0. No, I don't. **(SKIP TO A13)**
1. Yes, I know some of them.
2. Yes, I know all of them.

A12. Do you know the parents of the friends who often play together with this child?

0. No, I don't.
1. Yes, I know some of them.
2. Yes, I know all of them.

A13. How much time do you spend directly on this child (including taking care of his/her living, helping with his/her coursework, and entertainment) EVERY DAY ON AVERAGE?

[___|___] hours every day on average

A14. How often do you discuss the following with this child?

	Never	Sometimes	Often
Things happened at school	1	2	3
The relationship between he/she and his/her friends	1	2	3
The relationship between he/she and his/her teachers	1	2	3
his/her feelings	1	2	3
his/her worries and troubles	1	2	3

A15. When you are at home with this child, what language does he/she usually speak?

1. Mandarin Chinese
2. Sometimes dialect of his/her hometown, sometimes mandarin Chinese
3. Dialect of his/her hometown
4. Other

A16. When you are at home with this child, what language do you usually speak?

1. Mandarin Chinese
2. Sometimes dialect of my hometown, sometimes mandarin Chinese
3. Dialect of my hometown
4. Other

A17. How often did the family members do the following together with this child over the last year?

	Never	Once a Year	Once every half year	Once a Month	Once a Week	More Than Once a Week
Having dinner	1	2	3	4	5	6
Reading	1	2	3	4	5	6
Watching TV	1	2	3	4	5	6
Playing sports	1	2	3	4	5	6
Visiting museums, zoos, science museums, etc.	1	2	3	4	5	6
Going out to watch movies, shows, sports games, etc.	1	2	3	4	5	6

A18. What is the highest level of education do you expect this child to receive?

1. Drop out now
2. Graduate from junior high school
3. Go to technical secondary school or technical school
4. Go to vocational high school
5. Go to senior high school
6. Graduate from junior college
7. Get a bachelor degree
8. Get a Master degree
9. Get a Doctor degree

A19. What kind of job do you most expect this child to do in the future?

1. Government official, staff of public institutions, civil servant
2. Manager or administrator of enterprises/corporations
3. Scientist/engineer
4. Teacher/doctor/lawyer
5. Designer
6. Artistic performer/actor/host
7. Professional athlete
8. Technical worker (including driver)
9. Other (Please specify______________)
10. I don’t care

A20. Where do you most expect this child to live and work in the future?

1. In rural area
2. In small or medium cities
3. In big cities, such as Beijing/Shanghai/Guangzhou
4. Abroad
5. I don’t care

A21. Are you confident in the future of this child?

1. Not confident at all
2. Not so confident
3. Somewhat confident
4. Very confident

(Please continue to finish Part B.)

Part B: The Relationship between Parents and School

B1. Do this child's parents attend the parent meeting this semester?

1. The school held one, and the parents did attend.
2. The school held one, but the parents didn't attend.
3. Not held yet, but the parents are going to attend once held
4. Not held yet, and the parents are not going to attend if held

B2. How many times have this child's parents contacted the teacher at school this semester?

1. Never 2. Once 3. Two to four times 4. Five times or more

B3. When contacting the child's teacher this semester, what did the parents usually talk about with the teacher? (Please mark all that apply.)

0. The parents have never contacted the child's teacher this semester.

1. Schoolwork 2. The child's morals 3. Mental health
4. Physical health 5. Friends of the child 6. Other

B4. How many times has this child's teacher contacted the parents this semester?

1. Never 2. Once 3. Two to four times 4. Five times or more

B5. When the teacher contacted you this semester, what did he/she usually talk about with you? (Please mark all that apply.)

0. The teacher has never contacted me this semester.

1. Schoolwork 2. The child's morals 3. Mental health
4. Physical health 5. Friends of the child 6. Other

B6. Are you afraid of communicating with the school teacher?

1. Quite afraid 2. A little bit afraid 3. Not afraid at all

B7. In your opinion, is the teacher responsible for this child?

1. Not responsible at all 2. Not so responsible 3. Moderate
4. Somewhat responsible 5. Very responsible

B8. In your opinion, is the teacher patient to your child?

1. Not patient at all 2. Not so patient 3. Moderate
4. Somewhat patient 5. Very patient

B9. In your opinion, which kind of the following students benefits most from the teaching method of the teacher?

1. Students with academic record below average
2. Students with average academic record
3. Students with academic record above average
4. All kinds of students
5. It's hard to say.

B10. Do you agree that it is totally the teachers' responsibility to educate children?

1. Strongly disagree
2. Somewhat disagree
3. It's hard to say
4. Somewhat agree
5. Strongly agree

B11. Does this child like his/her teachers and schoolmates at school?

	Strongly dislike them	Somewhat dislike them	Somewhat like them	Like them very much
His/her homeroom teacher	1	2	3	4
Other teachers at school	1	2	3	4
His/her schoolmates	1	2	3	4

B12. Which of the following factors do you think have effects on students' grade? (Please mark all that apply.)

1. Parents' discipline
2. Teachers' teaching
3. Class
4. Friends
5. Attendance of cram school
6. Extent of hardworking
7. Talent and capability

B13. Do you agree that boys are better at mathematics than girls?

1. Yes, I do.
2. No, I don't.

B14. Do most people around you agree that boys are better at mathematics than girls?

1. Yes, they do.
2. No, they don't.

If this child has schoolmates from non-local county/district, please answer question B15-B20 according to the real condition; if this child doesn't have schoolmates from non-local county/district, please answer these questions according to your assumption:

B15. What kind of effect do you think will the increase of students from non-local county/district have on the educational quality of the school?

1. Harmful
2. Beneficial
3. No effect

B16. What kind of effect do you think will the increase of students from non-local county/district have on the atmosphere of the school?

1. Harmful   2. Beneficial   3. No effect

B17. Which kind of students do you think will the school teachers like better?

1. Students from local county/district
2. Students from non-local county/district
3. There is no difference

B18. When students from non-local county/district have conflicts with students from local county/district, whom do you think will the school teachers be partial to?

1. Students from local county/district
2. Students from non-local county/district
3. The teacher won't be partial to anyone

B19. Do you think the school teachers are prejudiced against students from non-local county/district?

1. Not prejudiced at all   2. A little bit prejudiced
3. Somewhat prejudiced   4. Very prejudiced

B20. Do you think the school teachers are prejudiced against the parents of students from non-local county/district?

1. Not prejudiced at all   2. A little bit prejudiced
3. Somewhat prejudiced   4. Very prejudiced

(Please continue to finish Part C.)

Part C: School Education

C1. From whom/which of the following did your family get to know the application and enrollment procedures of this school? (Please mark all that apply.)

1. The primary school that this child graduated from
2. The Sub-district office/township government
3. The Internet
4. Information provided by this school
5. Parents of other students
6. Other (Please specify: _____________)

C2. Which of the following did your family do in order to enroll this child in this school? (Please mark all that apply.)

1. Asking friends for help
2. Giving presents to the related government/school leaders
3. Paying extra fees (Please specify: __________)
4. Buying a house/apartment in the “education district” where this school is located
5. Changing the location of the Hukou of your family
6. Transferring the Hukou of your family or this child under other relatives or friends
7. Letting this child take all kinds of achievement tests
8. Other (Please specify: _____________)
9. We did none of the above.

C3. When enrolling this child in this school, which of the following certificates did you prepare? (Please mark all that apply.)

1. The booklet of Hukou (the official book of registered permanent residence)
2. The property ownership certificate or lease contract of your house/apartment
3. (Temporary) residential permission
4. Social security for at least one year
5. The family planning certificate
6. Business License or employment certificate
7. “Scores” meeting some requirement
8. Other (Please specify :______________)

C4. Is it difficult for your family to obtain the above certificates?

0. No, it isn’t.
1. Yes, it is. And among the above certificates:

   The most difficult to obtain is [__].

The second difficult to obtain is [__].

The third difficult to obtain is [__].

C5. What is the total fee paid to this school EVERY semester?

	**Fees**
The total fee paid to this school:	[__|__|__|__|__] Yuan (Please fill in 00000 if it is free.)
Including: Tuition fees	[__|__|__|__|__] Yuan (Please fill in 00000 if it is free.)
Book fees	[__|__|__|__|__] Yuan (Please fill in 00000 if it is free.)
Activity fees	[__|__|__|__|__] Yuan (Please fill in 00000 if it is free.)
Meal expenses	[__|__|__|__|__] Yuan (Please fill in 00000 if it is free.)
Dormitory fees	[__|__|__|__|__] Yuan (Please fill in 00000 if it is free, or fill in 99999 if this child does not live in the dorm)
Other fees (Please specify: ______)	[__|__|__|__|__] Yuan (Please fill in 00000 if it is free.)

C6. Does your child receive the governmental subsidies in education this semester? (Please mark all that apply.)

1. Yes, his/her book fees are exempted
2. Yes, the school provided free lunch
3. Yes, he/she received the grant aid for students ([__|__|__|__] Yuan/semester)
4. Other (Please specify: ___________________)
5. Not receiving any governmental subsidies

C7. How many students are there in this child's class?

[__|__|__] students

C8. Do you think the size of this child's class is too small or too big?

1. Too small 2. Somewhat small 3. Moderate
4. Somewhat big 5. Too big

C9. What do you think of the general attitude of this child towards schoolwork?

1. Not serious at all 2. Somewhat not serious 3. Moderate

4. Somewhat serious 5. Very Serious

C10. How does this child's academic record rank in his/her class at present?

1. Near the bottom 2. Below the average 3. About the average
4. Above the average 5. Around the top

C11. What is your requirement on this child's academic record?

1. Being one of the top five of his/her class 2. Above the average
3. About the average 4. No special requirement

If you are the parents from non-local county/district, please continue answering question C12-C14 in the following box. If you are the parents from local county/district, please skip to Part D.

C12. According to the local policy, is this child allowed to apply for a senior high school in this city (prefecture-level city)?

1. He/she is allowed to apply for a key senior high school.
2. He/she is allowed to apply for an ordinary senior high school, but not a key one.
3. He/she is not allowed to apply for either an ordinary senior high school or a key one. **(SKIP TO C14)**
4. Not clear **(SKIP TO C14)**

C13. Are there any special requirements that this child needs to meet in order to apply for an ordinary senior high school in this city (prefecture-level city)? (Please mark all that apply.)

0. There are no special requirements
1. Studying in the local (prefecture-level city) junior high school for three years
2. Local Hukou of this city (prefecture-level city) or enough “score” to obtain it
3. Local Hukou of this province
4. The property ownership certificate or lease contract of parents’ house/apartment
5. (Temporary) residential permission
6. Social security for at least one year
7. Family planning certificate
8. Business license or employment certificate
9. Other (Please specify :______________)

C14. What is your plan for this child after he/she graduates from junior high school?

1. To study in a senior high school in this city (prefecture-level city)
2. To study in a senior high school in his/her hometown
3. To study in a senior high school in another place
4. To study in a technical secondary school/ vocational high school in this city
5. To study in a technical secondary school/ vocational high school in this another place
6. To get a job
7. Other (Please specify: ________________)

(Please continue to finish Part D.)

Part D: Basic Information of This Child

D1. The birthplace of this child is:

1. In the local county/district
2. Not in the local county/district:

County (District): [_________], City: [_________],

Province (Autonomous Region/Municipality): [_________]

D2. The location of his/her Hukou[1] WHEN BORN is:

1. In the local county/district
2. Not in the local county/district:

County (District): [_________], City: [_________],

Province (Autonomous Region/Municipality): [_________]

D3. The location of his/her Hukou AT PRESENT is:

1. In the local county/district
2. Not in the local county/district:

County (District): [_________], City: [_________],

Province (Autonomous Region/Municipality): [_________]

D4. What is the type of his/her Hukou WHEN BORN? (Agricultural Hukou refers to rural Hukou, a record that identifies a person as a rural resident. Non-agricultural Hukou refers to urban Hukou. Residential Hukou is a general record of residence assigned to all residents of a certain region, regardless of rural or urban background)

1. Agricultural Hukou
2. Non-agricultural Hukou
3. Residential Hukou
4. Other

D5. What is the type of his/her Hukou AT PRESENT?

1. Agricultural Hukou **(SKIP TO D7)**
2. Non-agricultural Hukou
3. Residential Hukou
4. Other **(SKIP TO D7)**

D6. In which year did this child obtain the non-agricultural Hukou?

In the year of [__|__|__|__]

(Please fill in 9997 if this child obtained the non-agricultural Hukou since the beginning.)

D7. In which year did this child come to live in this county/district?

[1] Hukou refers to a record of household registration system in China that identifies a person as a resident of an area.

In the year of [__|__|__|__]

(Please fill in 9997 if this child has been living in this county/district since born.)

D8. Who was in charge of taking care of this child before he/she went to primary school?

1. His/her parents
2. His/her grandparents on mother's side
3. His/her grandparents on father's side
4. A Nanny
5. Other relatives of this child
6. Other non-relatives (not including the nanny)

D9. Which one of the following best describes the financial conditions of his/her family before this child went to primary school?

1. Very poor
2. Somewhat poor
3. Moderate
4. Somewhat rich
5. Very rich

D10. Has this child ever been seriously ill before he/she went to primary school?

1. Yes, he/she has.
2. No, he/she hasn’t.

D11. What is the birthweight of this child?

[__|__]. [__] jin

(Please notice that the unit of the weight is kilogram, and 1 decimal digit is reserved. Please fill in 00.0 if you are not clear about your exact birthweight.)

D12. What is the current height and weight of this child?

Height: [___|___|___] centimeters

Weight: [___|___|___] jin

(Please notice that the unit of the height and weight is centimeter and jin, respectively.)

D13. Which one of the following best describes the general health condition of this child at present?

1. Very poor
2. Not very good
3. Moderate
4. Good
5. Very good

D14. Is this child short-sighted at present?

1. Yes. he/she is

   The strength of his/her left eyeglass is [__|__|__] degrees[2].

   The strength of his/her right eyeglass is [__|__|__] degrees.

2. No. he/she isn’t

[2] Degree is the unit of measurement of optical power of lens or glasses used in China, 100 degrees of short-sightedness = -1dioptre.

D15. Does your child have the following disorders at present? (Please mark all that apply.)

0. None
1. Vision disorders (not including short-sighted)
2. Hearing disorders
3. Extremity Disabled
4. Language Disorders
5. Autism or other mental disorders
6. Attention deficit hyperactivity disorder (ADHD)
7. Other (Please specify: _____________)

D16. Which of the following medical insurance does this child have at present? (Please mark all that apply.)

1. Reimburse for child’s medical expenses by parents’ work units
(The proportion of the reimbursement of the total medical expenses is [__|__|__]%.)
2. The basic medical insurance for urban residents
3. The new rural cooperative medical insurance
4. The medical insurance for children
5. Commercial medical insurance
6. Other medical insurance (Please specify: _____________________)
7. This child doesn’t have any medical insurance at present.

D17. Does this child fit in the following descriptions?

	**Not fit at all**	**Somewhat not fit**	**Somewhat fit**	**Exactly fit**
Able to express himself/ herself clearly	1	2	3	4
Able to give quick responses	1	2	3	4
A faster learner	1	2	3	4
Curious about new stuff	1	2	3	4

(Please continue to finish Part E.)

Part E: Parents, Family and Community

E1. In which year were you born?

In the year of [___|___|___|___]

E2. Your ethnic nationality is:

1. The Han nationality
2.The Mongol nationality
3.The Manchu nationality
4. The Hui nationality
5.The Tibetan nationality
6.The Zhuang nationality
7. The Uygur nationality
8.Other (Please specify: __________)

E3. Where do you live at present?

1. In the local county/district
2. Not in the local county/district

E4. What is the type of your Hukou at present?

1. Agricultural Hukou **(SKIP TO E6)**
2. Non-agricultural Hukou
3. Residential Hukou
4. Other **(SKIP TO E6)**

E5. In which year did you obtain the non-agricultural Hukou?

In the year of [__|__|__|__]

(Please fill in 9997 if this child obtained the non-agricultural Hukou since the beginning.)

E6. What is your political affiliation?

1. Member of the Communist Party
2. Member of a Democratic Party
3. The masses

E7. What is the highest education level you have completed?

1. None
2. Finished elementary school
3. Junior high school degree
4. Technical secondary school or technical school degree
5. Vocational high school degree
6. Senior high school degree
7. Junior college degree
8. Bachelor degree
9. Master degree or higher

E8. What is your current occupation?

1. Government official, staff of public institutions, civil servant
2. Middle/Senior management personnel of enterprises/corporations

3. Teacher, engineer, doctor, lawyer
4. Technical worker (including driver)
5. Ordinary staff or worker in production or manufacturing industry
6. Ordinary staff or worker in business or service industry
7. Self-employed worker
8. Farmer
9. Unemployed or laid-off worker
10. Other (Please specify: __________)

E9. Which one of the following best describes your general health condition at present, compared with others at your age?

1. Very poor 2. Not very good 3. Moderate
4. Good 5. Very good

E10. How much time do you usually spend on the following every day in your spare time?

	Never	Less than one hour	one to two hours	More than two hours
Reading (including newspapers and magazines)	1	2	3	4
Watching TV	1	2	3	4
Surfing on the Internet	1	2	3	4

E11. How often do you do the following?

	Never	Several Times a Year	Several Times a Month	Several times a Week	Every Day
Drinking alcohol	1	2	3	4	5
Smoking	1	2	3	4	5

E12. Which of the following people live in the same household with this child at present? (Please mark all that apply.)

1. Father of this child
2. Mother of this child
3. Full or half sibling(s) of this child
4. Spouse of this child’s sibling(s)
5. Grandparent(s) of this child on mother's/father's side
6. Other relative(s) of this child
7. Other non-relative(s)

E13. Which of the following immediate relatives of this child do not live in the same household with him/her at present? (Please mark all that apply.)

1. Father of this child
2. Mother of this child
3. Full or half sibling(s) of this child
4. All the immediate relatives (if there is any) of this child live with him/her.

E14. What is the location of your Hukou at present?

1. In the local county/district **(SKIP TO E19)**
2. Not in the local county/district:
   County (District): [_________], City: [_________],
   Province (Autonomous Region/Municipality): [_________]

E15. Does this child have any FULL OR HALF siblings living in the hometown?

1. No, he/she doesn't. **(SKIP TO E19)**
2. Yes, he/she has [___] sibling(s) living in the hometown.

E16. Whom do his/her sibling(s) in the hometown live with?

1. Father/Mother
2. Grandparent(s) on mother's/father's side
3. Other Relative(s)
4. Other

E17. How old is the eldest one among these sibling(s) living in the hometown at present?

[__|__] years old

E18. Is there any one of these sibling(s) attending schools at present?

0. No, there isn't.
1. Yes, the highest grade that they are in is Grade [__|__]

E19. Which one of following best describes the financial conditions of your family at present?

1. Very poor
2. Somewhat poor
3. Moderate
4. Somewhat rich
5. Very rich

E20. Do your family receive subsistence allowance at present?

1. Yes, we do.
2. No, we don't.

E21. Which one of following best describes the income level of your family compared with that of others in your community?

1. Very Low
2. Somewhat low
3. Average
4. Somewhat high
5. Very high

E22. Is there anyone in your family that needs long-term care because of illness or mobility problems?

1. Yes, there is. 2. No, there isn't.

E23. What type of house/apartment do you live in at present?

1. Temporary work shed
2. Basement
3. Rural bungalow
4. Rural building with more than one story
5. Urban bungalow
6. Ordinary urban apartment
7. Urban housing estate
8. Other (Please specify: ____________)

E24. Is the building/house/apartment you are living in also used for production/business?

1. Yes, it is. 2. No, it isn't.

E25. How many rooms are there in the building/house/apartment you are living in? (Including living rooms and bedrooms, but not including kitchens or bathrooms)

[__|__] room(s)

E26. Is there tap water (running water) in your building/house/apartment?

1. Yes, there is. 2. No, there isn't.

E27. Is there a private toilet in your building/house/apartment?

1. Yes, there is. 2. No, there isn't.

E28. Which kind of the following toilets are used by your family at present?

1. Flush Toilet 2. Squat Toilet 3. Cement Cesspit 4. Soil Cesspit

E29. Which kind of area are you living in?

1. Central area of the city/county
2. Outskirts of the city/county
3. The "rural-urban continuum" area of the city/county
4. Towns outside the city/county
5. Rural area
6. Other (Please specify: ____________)

E30. Which kind of community are you living in?

1. Old-style city community without reconstruction
2. Single or compound community affiliated to a work unit (Danwei)
3. Ensuring housing supplied by social secure/subsidy system

4. Ordinary commercial residential building
5. Elite housing
6. Villages inside the city (“Cheng Zhong Cun”)
7. Rural Area
8. Other (Please specify: ____________)

E31. Which kind of neighborhood are you living with? (Please mark all that apply.)

1. Local peasants
2. Local labor workers
3. Some are migrant workers
4. Most are migrant workers
5. Teachers/doctors/engineers/civil servants
6. Senior management personnel of enterprises/corporations
7. Other
8. Not clear

E32. Are there any of the following facilities provided in the community you live in? (Please mark all that apply.)

1. Public transportation   2. Kindergarten   3. Hospital   4. Park

E33. Do you agree with the following statements about the community you live in?

	Strongly disagree	Somewhat disagree	Somewhat disagree	Strongly agree
Safe	1	2	3	4
Clean	1	2	3	4
Hardly any pollution	1	2	3	4

Part F: Contact Information

We hope to continue the tracking of the growth and improvement of this child in the next year by follow-up surveys. Please leave the contact information of the parents, so that we could keep in touch with this child and his/her parents:

F1. Telephone number of this child’s family: ________________________

F2. The contact information of this child's father

Name: ________________ Mobile phone number: ____________________

F3. The contact information of this child's mother

Name: ________________ Mobile phone number: ____________________

This is the end of the questionnaire. Thank you very much for your support!

We wish you a healthy and happy life!

Class: ________

Name of Subject Teacher: ____________

Name of School: ____________________

Location of School: County/District:___________ City: ___________

Province (Autonomous Region/Municipality):____________

China Education Panel Survey

Academic Year 2013-2014

Subject Teacher Questionnaire

Teachers answering this questionnaire do not need to fill in the blanks in the box below.

1. Code of Questionnaire: [____|____|____|____|____|____]

2. Code of County/District: [____|____|____|____|____|____]

3. Verifier: ______________________

Distinguished teachers,

Hello!

China Education Panel Survey (CEPS) is the first large-scale, nationwide, and longitudinal social survey of junior high students in China. The purpose of the CEPS is to provide detailed and reliable data for the research of the current situation and future development of China's primary education.

CEPS is jointly conducted by the National Survey Research Center (NSRC) at Renmin University of China and academic institutions throughout the country. During the academic year of 2013-2014, we are going to survey nearly 20,000 students as well as their parents, homeroom teachers, subject teachers and school administrators. Through scientific sampling, your class has been chosen as the sample class. We would appreciate it very much if you could help us finish this questionnaire. Thank you for your understanding and support.

There are neither right nor wrong answers to these questions and your answers will not be evaluated as part of your work performance or that of your school. Therefore, please write down your honest ideas and the reality of the sample class. We pledge here that under *the Statistics Law of the People's Republic of China*, we will hold all the information you are going to provide in strict confidence and it will never be given away to any individual or institution. Thank you for your cooperation.

When answering a multiple-choice question, please circle the number of your choice; when answering a blank-filling question, please write down words or numbers on the lines in the square brackets.

National Survey Research Center (NSRC), Renmin University of China

March, 2014

Part A: Teaching

A1. What subject do you teach for this class?

1. Math   2. Chinese   3. English

A2. When did you begin to teach this class?

In the year of [__|__|__|__]

A3. How was the academic performance of your class compared with other classes of the same grade *WHEN YOU BEGAN TEACHING THIS CLASS*?

1. Near the bottom
2. Below average
3. Average
4. Above average
5. Among the best

A4. At present, how is the academic performance of your class compared with other classes of the same grade?

1. Near the bottom
2. Below average
3. Average
4. Above average
5. Among the best

A5. At present is the grade to which the sample class belongs divided according to the score of the subject you teach?

1. Yes   2. No

A6. How many other classes do you teach besides the sample class?

[__|__] class(s)

A7. How many class hours did you teach last week?

[__|__] hour(s)

A8. How many hours did you spend on preparing lessons last week?

[__|__] hour(s) (Please fill in 00 if you don’t need to prepare lessons.)

A9. How many hours did you spend on correcting homework and/or test papers last week?

[__|__] hour(s) (Please fill in 00 if you don’t need to correct homework or test papers.)

A10. How long was your total working hours last week?

[___|___|___] hour(s) (including teaching, preparing lessons, correcting homework and/or test papers, and home visiting.)

A11. Do you often discuss the subject you teach with your colleagues since the beginning of this semester?

1. Yes, I do
2. No, I don't

A12. What's the most discussed teaching-related topic between you and your colleagues?

1. Content of textbooks
2. Teaching methods
3. Exam paper design
4. Students' tutoring
5. Other (Please specify: __________)

A13. How often do you apply each of the following teaching methods to the sample class?

	Never	Seldom	Sometimes	Often	Always
Lectures	1	2	3	4	5
Group discussion	1	2	3	4	5
Interaction between the teacher and students	1	2	3	4	5

A14. How often do you adopt each of the following facilities in your teaching?

	Never	Seldom	Sometimes	Often	Always
Multimedia projector	1	2	3	4	5
Internet	1	2	3	4	5
Wall maps, models, posters	1	2	3	4	5
Personal teaching website, blog or microblog	1	2	3	4	5

A15. What's the proportion of each of the following teaching materials used in your teaching?

	Percentage
Domestic textbooks	[__|__|__]%
Foreign textbooks	[__|__|__]%
Reference materials	[__|__|__]%
Teaching materials designed by yourself	[__|__|__]%
Total	100%

A16. Take the main subject you teach as an example, how great is the influence of each of the following elements on students' academic record?

	Almost no influence	Some influence	Very great influence
Students’ talent	1	2	3
Students’ attitude to learning	1	2	3
Students’ method of learning	1	2	3
Students’ family background	1	2	3
Students’ circle of friends	1	2	3
Teachers’ teaching method	1	2	3
The amount of attention paid to students by the teacher	1	2	3
Teachers’ salary	1	2	3
The school’s management	1	2	3
The school’s teaching facilities	1	2	3

A17. How much pressure do you have concerning the following aspects?

	None	Low	Moderate	High	Very high
Students’ poor academic records	1	2	3	4	5
Students’ uneven academic records	1	2	3	4	5
School’s evaluation of teachers	1	2	3	4	5
School’s administrative measures	1	2	3	4	5
Requirements of students’ parents	1	2	3	4	5
Students’ enrollment rate	1	2	3	4	5
Public opinions about teachers	1	2	3	4	5
Publication of academic papers	1	2	3	4	5

A18. Generally speaking, how many students in this class pay respect to you?

1. Very few
2. Less than half
3. Half of the class
4. More than half
5. Almost everyone

A19. According to your understanding or that of other teachers, how is the academic foundation of students from non-local counties/districts compared with that of students from local county/district?

	Far worse	About the same	Better
Math	1	2	3
Chinese	1	2	3
English	1	2	3
Physics	1	2	3
Chemistry	1	2	3
Biology	1	2	3

A20. What kind of teaching method do you think best suits the following types of classes?

	Lectures	Group discussion	Interaction between the teacher and students
Classes composed of only local students	1	2	3
Classes in which the majority are local students and only a small number are from non-local counties/districts	1	2	3
Classes in which more than one third are from non-local counties/districts	1	2	3

A21. What's your opinion of the following aspects of each type of classes?

	**Effect of teaching:**<br><br>1. Very bad<br>2. Bad<br>3. Moderate<br>4. Good<br>5. Very good	**Class discipline:**<br><br>1. Very bad<br>2. Bad<br>3. Moderate<br>4. Good<br>5. Very good	**Relations among students:**<br><br>1. Very bad<br>2. Bad<br>3. Moderate<br>4. Good<br>5. Very good
Classes composed of only local students	[___]	[___]	[___]
Classes in which the majority are local students and only a small number are from non-local counties/districts	[___]	[___]	[___]
Classes in which more than one third are from non-local counties/districts	[___]	[___]	[___]

A22. What influence do you think non-local students will have on the whole class concerning class management?

1. The class would become more difficult to handle
2. Nothing much would change
3. The class would become easier to handle

A23. Which type of class would you like to be in charge of if you can make a choice?

1. Classes composed of only local students
2. Classes in which the majority are local students and only a small number are from non-local counties/districts
3. Classes in which more than one third are from non-local counties/districts
4. I don't care

A24. This question is for homeroom teachers of grade 9: what measures have you taken to prepare the students for the high school entrance exam? (Please mark all that apply. Please skip to Question A25 if you are a homeroom teacher of grade 7.)

0. None
1. Finishing teaching all courses ahead of schedule, and carrying out a general review
2. Arranging a series of tests
3. Arranging after-class tutoring
4. Other (Please specify: __________)

A25. Please write down NAMES of five students in the class in ascending order of their daily behavior, with Student 1 being the worst and Student 5 being the best. After that, please answer: how much do you agree with each of the following aspects about each of these students?

1. Strongly disagree 2. Somewhat disagree 3. Somewhat agree 4. Strongly agree

	**Student 1**	**Student 2**	**Student 3**	**Student 4**	**Student 5**
**Names**	**[_______]**	**[_______]**	**[_______]**	**[_______]**	**[_______]**
His/her performance does not match his/her true capability	[__]	[__]	[__]	[__]	[__]
He/she always volunteers to answer questions in class.	[__]	[__]	[__]	[__]	[__]
He/she gets along well with other students.	[__]	[__]	[__]	[__]	[__]
He/she actively participates in class activities.	[__]	[__]	[__]	[__]	[__]
I often communicate with this student.	[__]	[__]	[__]	[__]	[__]
I often ask him/her to answer my questions in class.	[__]	[__]	[__]	[__]	[__]
I hope this student can continue his/her study after graduating from junior high.	[__]	[__]	[__]	[__]	[__]

(Please continue to finish Part B.)

Part B: Background and Opinions

B1. Your sex is:
1. Male 2.Female

B2. Your year of birth is: [__|__|__|__] ([YYYY])

B3. What is your current marital status?
1. Not married 2. Married 3. Widowed
4. Divorced/separated 5. Other

B4. Up till now, what is the highest educational diploma you have attained?
1. Junior high school degree or below
2. Vocational high school/technical secondary school/technical school degree
3. Senior high school degree
4. Junior college degree
5. Bachelor degree (attained through adult higher education)
6. Bachelor degree (attained through regular higher education)
7. Master degree or higher

B5. Are you a graduate from pedagogical universities or pedagogical majors?
1. Yes. 2. No.

B6. Do you have a teacher certification?
1. Yes. 2. No.

B7. How many years of experience do you have in teaching?
[__|__] year(s)

B8. Have you ever taught in other schools before you came to this school?
1. No **(SKIP TO C10)** 2. Yes

B9. Please write down in chronological order the beginning and ending year of schools you have worked in, the location and type of these schools, and whether they enroll students from non-local counties/districts?

	**Location of school:**<br><br>1. In a village<br>2. In a town<br>3. In a county<br>4. In a prefecture-level city<br>5. In a capital city<br>6. In a municipality	**Type of school:**<br><br>1. Public school<br>2. Privately-run and state-subsidized school<br>3. Ordinary private school<br>4. Private school for migrant children	**Did it enroll students from non-local counties/districts?**<br><br>1. Yes<br>2. No
From [__|__|__|__]<br>to [__|__|__|__] (YYYY)	[___]	[___]	[___]
From [__|__|__|__]<br>to [__|__|__|__] (YYYY)	[___]	[___]	[___]

From [__|__|__|__]<br>to [__|__|__|__] (YYYY)	[___]	[___]	[___]
From [__|__|__|__]<br>to [__|__|__|__] (YYYY)	[___]	[___]	[___]

B10. When did you begin to teach in this school?

[__|__|__|__][__|__] ([YYYY][MM])

B11. Are you an institutionally registered teacher admitted by the government?

1. Yes, I became a registered teacher in the year [__|__|__|__](YYYY).
2. No, I'm not.

B12. Do you have a professional title in teaching? If yes, what is it?

0. No, I don't.
1. Level-C Teacher 2. Level-B Teacher 3. Level-A Teacher
4. Senior-Level Teacher 5. Principal Senior-Level Teacher

B13. Are you holding other concurrent administrative posts? If yes, what is it?

0. No, I'm not
1. Head of the grade
2. Head of the teaching & research group of a subject
3. Teaching director or deputy teaching director
4. Deputy principal
5. Principal

B14. Do you receive government subsidies?

1. Yes 2. No

B15. Which of the following grades have you ever taught? (Please mark all that apply.)

1. The seventh grade 2. The eighth grade 3. The ninth grade
4. Elementary school grades 5. Senior high school grades

B16.Which of the following awards have you ever won because of your teaching performance? (Please mark all that apply.)

1. National award 2. Province-level award 3. City-level award
4. County-level award 5. School-level award 6. None

B17. Have you ever won the title of Excellent Teacher over the past three years?

1. Yes, more than once 2. Yes, I got the title once
3. No 4. There is no such title in this school's evaluation system

B18. In general, are you satisfied with your current job as a teacher?

1. Strongly unsatisfied 2. Somewhat unsatisfied 3. Moderate
4. Somewhat satisfied 5. Strongly satisfied

B19. How many close friends do you have among your colleagues in this school?

1. None 2. Just a few 3. Some 4. Many

B20. Have you ever feel tired of being a teacher?

1. Never 2. Seldom 3. Sometimes 4. Often

B21. Are you still willing to choose teacher as your job if you have a second chance to make a choice?

1. No 2. Not sure 3. Definitely yes

B22. Are you still willing to become a junior-high teacher if you have a second chance to make a choice?

1. No 2. Not sure 3. Definitely yes

B23. Are you still willing to teach in this school if you have a second chance to choose an institution to work for?

1. No 2. Not sure 3. Definitely yes

B24. Are you satisfied with each of the following aspects of this school?

	Strongly unsatisfied	Somewhat unsatisfied	Moderate	Somewhat satisfied	Strongly satisfied
Salary	1	2	3	4	5
School management	1	2	3	4	5
Facility	1	2	3	4	5
Students' quality	1	2	3	4	5

B25. What do you think of the school's academic atmosphere?

1. Very bad 2. Bad 3. Moderate 4. Good 5. Very good

B26. What do you think of the school's management of students?

1. Very loose 2.Loose 3. Moderate 4.Strict 5. Very strict

B27. In your opinion, what are the capabilities or qualities of students that the school needs to improve its educating further? (Please mark all that apply.)

1. The capability of analyzing and solving problems
2. Creativity and imagination
3. Self-discipline and perseverance
4. Positive values and morality
5. Interpersonal relationship, communication and cooperation
6. Mental health
7. Concerns for and engagement in public affairs

B28. In what aspects do you think the school should bear more responsibility than students' family? (Please mark all that apply.)

1. The capability of analyzing and solving problems
2. Creativity and imagination
3. Self-discipline and perseverance
4. Positive values and morality

5. Interpersonal relationship, communication and cooperation
6. Mental health
7. Concerns for and engagement in public affairs

B29. What do you think is the most important goal of school education? (Please mark all that apply.)

1. To foster virtue
2. To pass on basic knowledge
3. To foster comprehensive capabilities
4. To increase senior high school/college/university acceptance rate
5. To make sure students can grow up happily and safely

Part C: Contact Information

We hope to continue the tracking of the students' future progress through this panel survey. Please leave your contact information so that we could keep in touch with you and your students:

Your office phone number: ______________

Your mobile phone number: _____________

Your Email address: ___________________

Your QQ number: _____________________

This is the end of the questionnaire. Thank you for your engagement and cooperation! We wish you a good health and successful career!

Class: ________

Name of Homeroom Teacher: ____________

Name of School: ___________________

Location of School: County/District:___________ City: ___________

Province (Autonomous Region/Municipality):____________

China Education Panel Survey

Academic Year 2013-2014

Homeroom Teacher Questionnaire

Teachers answering this questionnaire do not need to fill in the blanks in the box below.

1. Code of Questionnaire: [____|____|____|____|____|____]

2. Code of County/District: [____|____|____|____|____|____]

3. Verifier: ______________________

Distinguished teachers,

Hello!

China Education Panel Survey (CEPS) is the first large-scale, nationwide, and longitudinal social survey of junior high students in China. The purpose of the CEPS is to provide detailed and reliable data for the research of the current situation and future development of China's primary education.

CEPS is jointly conducted by the National Survey Research Center (NSRC) at Renmin University of China and academic institutions throughout the country. During the academic year of 2013-2014, we are going to survey nearly 20,000 students as well as their parents, homeroom teachers, subject teachers and school administrators. Through scientific sampling, your class has been chosen as the sample class. We would appreciate it very much if you could help us finish this questionnaire. Thank you for your understanding and support.

There are neither right nor wrong answers to these questions and your answers will not be evaluated as part of your work performance or that of your school. Therefore, please write down your honest ideas and the reality of the sample class. We pledge here that under *the Statistics Law of the People's Republic of China*, we will hold all the information you are going to provide in strict confidence and it will never be given away to any individual or institution. Thank you for your cooperation.

When answering a multiple-choice question, please circle the number of your choice; when answering a blank-filling question, please write down words or numbers on the lines in the square brackets.

National Survey Research Center (NSRC), Renmin University of China

March, 2014

Part A: Teaching

A1. What subject(s) do you teach as the homeroom teacher of this class? (Please mark all that apply.)

1. Math 2. Chinese 3. English 4. Physics
5. Chemistry 6. Biology 7. History 8. Geography
9. Politics 10. Other (Please specify: __________)

A2. When did you take the post of homeroom teacher of this class?

In the year of [__|__|__|__]

A3. How was the academic performance of your class compared with other classes of the same grade when you took the post of homeroom teacher of this class?

1. Near the bottom
2. Below average
3. Average
4. Above average
5. Among the best

A4. At present, how is the academic performance of your class compared with other classes of the same grade?

1. Near the bottom
2. Below average
3. Average
4. Above average
5. Among the best

A5. At present, is the grade to which the sample class belongs divided according to the total score or the score of a single subject?

1. Yes 2. No

A6. How many other classes do you teach besides the sample class?

[__|__] class(s)

A7. How many class hours did you teach last week?

[__|__] hour(s)

A8. How many hours did you spend on preparing lessons last week?

[__|__] hour(s) (Please fill in 00 if you don't need to prepare lessons.)

A9. How many hours did you spend on correcting homework and/or test papers last week?

[__|__] hour(s) (Please fill in 00 if you don't need to correct homework or test papers.)

A10. How long was your total working hours last week?

[___|___|___] hour(s) (including teaching, preparing lessons, correcting homework and/or test papers, and home visiting.)

A11. Do you often discuss the subject you teach with your colleagues since the beginning of this semester?

1. Yes, I do
2. No, I don't

A12. What's the most discussed teaching-related topic between you and your colleagues?

1. Content of textbooks
2. Teaching methods
3. Exam paper design
4. Students' tutoring
5. Other (Please specify: __________)

A13. How often do you apply each of the following teaching methods to the sample class?

	Never	Seldom	Sometimes	Often	Always
Lectures	1	2	3	4	5
Group discussion	1	2	3	4	5
Interaction between the teacher and students	1	2	3	4	5

A14. How often do you adopt each of the following facilities in your teaching?

	Never	Seldom	Sometimes	Often	Always
Multimedia projector	1	2	3	4	5
Internet	1	2	3	4	5
Wall maps, models, posters	1	2	3	4	5
Personal teaching website, blog or microblog	1	2	3	4	5

A15. What's the proportion of each of the following teaching materials used in your teaching?

	Percentage
Domestic textbooks	[__|__|__]%
Foreign textbooks	[__|__|__]%
Reference materials	[__|__|__]%
Teaching materials designed by yourself	[__|__|__]%
Total	100%

A16. Take the main subject you teach as an example, how great is the influence of each of the following elements on students' academic record?

	Almost no influence	Some influence	Very great influence
Students' talent	1	2	3
Students' attitude to learning	1	2	3
Students' method of learning	1	2	3
Students' family background	1	2	3
Students' circle of friends	1	2	3
Teachers' teaching method	1	2	3
The amount of attention paid to students by the teacher	1	2	3
Teachers' salary	1	2	3
The school's management	1	2	3
The school's teaching facilities	1	2	3

A17. How much pressure do you have concerning the following aspects?

	None	Low	Moderate	High	Very high
Students' poor academic records	1	2	3	4	5
Students' uneven academic records	1	2	3	4	5
School's evaluation of teachers	1	2	3	4	5
School's administrative measures	1	2	3	4	5
Requirements of students' parents	1	2	3	4	5
Students' enrollment rate	1	2	3	4	5
Public opinions about teachers	1	2	3	4	5
Publication of academic papers	1	2	3	4	5

A18. Generally speaking, how many students in this class pay respect to you?

1. Very few
2. Less than half
3. Half of the class
4. More than half
5. Almost everyone

A19. According to your understanding or that of other teachers, how is the academic foundation of students from non-local counties/districts compared with that of students from local county/district?

	Far worse	About the same	Better
Math	1	2	3
Chinese	1	2	3
English	1	2	3
Physics	1	2	3
Chemistry	1	2	3
Biology	1	2	3

A20. What kind of teaching method do you think best suits the following types of classes?

	Lectures	Group discussion	Interaction between the teacher and students
Classes composed of only local students	1	2	3
Classes in which the majority are local students and only a small number are from non-local counties/districts	1	2	3
Classes in which more than one third are from non-local counties/districts	1	2	3

A21. What's your opinion of the following aspects of each type of classes?

	**Effect of teaching:**<br><br>1. Very bad<br>2. Bad<br>3. Moderate<br>4. Good<br>5. Very good	**Class discipline:**<br><br>1. Very bad<br>2. Bad<br>3. Moderate<br>4. Good<br>5. Very good	**Relations among students:**<br><br>1. Very bad<br>2. Bad<br>3. Moderate<br>4. Good<br>5. Very good
Classes composed of only local students	[___]	[___]	[___]
Classes in which the majority are local students and only a small number are from non-local counties/districts	[___]	[___]	[___]
Classes in which more than one third are from non-local counties/districts	[___]	[___]	[___]

A22. What influence do you think non-local students will have on the whole class concerning class management?

1. The class would become more difficult to handle
2. Nothing much would change
3. The class would become easier to handle

A23. Which type of class would you like to be in charge of if you can make a choice?

1. Classes composed of only local students
2. Classes in which the majority are local students and only a small number are from non-local counties/districts
3. Classes in which more than one third are from non-local counties/districts
4. I don't care

A24. This question is for homeroom teachers of grade 9: what measures have you taken to prepare the students for the high school entrance exam? (Please mark all that apply. Please skip to Question A25 if you are a homeroom teacher of grade 7.)

0. None
1. Finishing teaching all courses ahead of schedule, and carrying out a general review
2. Arranging a series of tests
3. Arranging after-class tutoring
4. Other (Please specify: __________)

A25. Please write down NAMES of five students in the class in ascending order of their daily behavior, with Student 1 being the worst and Student 5 being the best. After that, please answer: how much do you agree with each of the following aspects about each of these students?

1. Strongly disagree 2. Somewhat disagree 3. Somewhat agree 4. Strongly agree

	**Student 1**	**Student 2**	**Student 3**	**Student 4**	**Student 5**
**Names**	**[_______]**	**[_______]**	**[_______]**	**[_______]**	**[_______]**
His/her performance does not match his/her true capability	[__]	[__]	[__]	[__]	[__]
He/she always volunteers to answer questions in class.	[__]	[__]	[__]	[__]	[__]
He/she gets along well with other students.	[__]	[__]	[__]	[__]	[__]
He/she actively participates in class activities.	[__]	[__]	[__]	[__]	[__]
I often communicate with this student.	[__]	[__]	[__]	[__]	[__]
I often ask him/her to answer my questions in class.	[__]	[__]	[__]	[__]	[__]
I hope this student can continue his/her study after graduating from junior high.	[__]	[__]	[__]	[__]	[__]

(Please continue to finish Part B.)

Part B: Class Management

B1. How much do you agree with each of the following aspects about the PARENTS of the five students you have chosen in Question A25?

1. Strongly disagree 2. Somewhat disagree 3. Somewhat agree 4. Strongly agree

	**Parents 1**	**Parents 2**	**Parents 3**	**Parents 4**	**Parents 5**
They care for their child's academic performance.	[__]	[__]	[__]	[__]	[__]
They check up with their child's homework carefully.	[__]	[__]	[__]	[__]	[__]
They care for their child's morality and mental health.	[__]	[__]	[__]	[__]	[__]
They care for their child's behaviors and habits.	[__]	[__]	[__]	[__]	[__]
They care for whom their child makes friends with.	[__]	[__]	[__]	[__]	[__]
They care for issues concerning their child's further education.	[__]	[__]	[__]	[__]	[__]
They have a high expectation on their child.	[__]	[__]	[__]	[__]	[__]
They often communicate with the homeroom teacher.	[__]	[__]	[__]	[__]	[__]
They are willing to cooperate with the homeroom teacher.	[__]	[__]	[__]	[__]	[__]
They think children's education depends only on school teachers.	[__]	[__]	[__]	[__]	[__]
They can spare time to accompany their child.	[__]	[__]	[__]	[__]	[__]
Generally speaking, they concern for their child's development.	[__]	[__]	[__]	[__]	[__]

B2. How many parents of your students do you know about? (Here "know about" means that you have met and talked with them, and know their basic situation.)

1. None 2. Just a few 3. About a half 4. More than half 5. All of them

B3. How do you usually contact the parents of your students? Please write down the number of 3 most frequently used means of contact.

1. Home visits 2. Phone calls 3.Text messages 4. E-mails
5. QQ 6. Other 7. There's no need to contact parents

The most frequently used means is [__], accounting for [__|__|__]%.
The second most frequently used means is [__].
The third most frequently used means is [__].

B4. How often have you had conflicts with the parents of your students since the beginning of this semester? (Such as quarrelling with the parents due to issues concerning students' academic record, class management, etc. or being reported to the school administrators by the parents.)

	**The number of parents you have had conflicts with:**<br><br>1. None<br>2. One to four<br>3. Five or above	**The major means you have adopted to settle these conflicts:**<br><br>1. Your negotiation with the parents<br>2. The school's negotiation with the parents<br>3. Higher educational departments' negotiation with the parents<br>4. Other
Students' academic performance	[__]	[__]
Students' discipline	[__]	[__]
Class management	[__]	[__]
Tuition or other charges	[__]	[__]
Other (Please specify: __________)	[__]	[__]

B5. Generally speaking, how many parents of your students pay respect to you?

1. None 2. Just a few 3. About a half 4. More than half 5. All of them

B6. How many students in your class have a habit of smoking or drinking?

1. None 2. A few 3. Many 4. A great number

B7. How many students in your class frequently go to Internet cafes or video arcades?

1. None 2. A few 3. Many 4. A great number

B8. How much time do you spend on communicating with your students after class everyday as a homeroom teacher?

[__|__] hours and [__|__] minutes

B9. In your class, what are the numbers of students with each of the following types of Hukou[1]? (Please fill in 000 if none.)

	**The number of students**
Hukou of the local county/district	[__|__|__]
Hukou of non-local counties/districts of this province	[__|__|__]
Hukou of other provinces	[__|__|__]

[1]Hukou refers to a record of household registration system in China that identifies a person as a resident of an area.

B10. Among the students in your class that are from the LOCAL county/district, what's the percentage of those from each of the following family background? (Please circle "9" if no student in your class has a local Hukou.)

Family background	Percentage	Nobody is from the local county/district
Peasants	[__|__|__]%	9
Workers	[__|__|__]%	9
Small business owners	[__|__|__]%	9
Intellectuals	[__|__|__]%	9
Managers or administrators of enterprises/corporations	[__|__|__]%	9
Government officials or civil servants	[__|__|__]%	9
Others	[__|__|__]%	9
Total	100%	9

B11. Among the students in your class that are from NON-LOCAL counties/districts, what's the percentage of those from each of the following family background? (Please circle "9" if no student in the class has a local Hukou)

Family background	Percentage	Nobody is from non-local counties/districts
Peasants	[__|__|__]%	9
Workers	[__|__|__]%	9
Small business owners	[__|__|__]%	9
Intellectuals	[__|__|__]%	9
Managers or administrators of enterprises/corporations	[__|__|__]%	9
Government officials or civil servants	[__|__|__]%	9
Others	[__|__|__]%	9
Total	100%	9

B12. Do you think the parents of local students would support or oppose that there are non-local students in class?

1. Strongly oppose 2. Somewhat oppose 3. Neither oppose nor support
4. Somewhat support 5. Strongly support

B13. Do you think the parents of local students would be willing to communicate with the parents of non-local students?

1. Very unwilling to 2. Somewhat unwilling to 3. Moderate
4. Somewhat willing to 5. Very willing to

(Please continue to finish Part C.)

Part C: Background and Opinions

C1. Your sex is:
1. Male 2.Female

C2. Your year of birth is: [__|__|__|__] ([YYYY])

C3. What is your current marital status?
1. Not married 2. Married 3. Widowed
4. Divorced/separated 5. Other

C4. Up till now, what is the highest educational diploma you have attained?
1. Junior high school degree or below
2. Vocational high school/technical secondary school/technical school degree
3. Senior high school degree
4. Junior college degree
5. Bachelor degree (attained through adult higher education)
6. Bachelor degree (attained through regular higher education)
7. Master degree or higher

C5. Are you a graduate from pedagogical universities or pedagogical majors?
1. Yes. 2. No.

C6. Do you have a teacher certification?
1. Yes. 2. No.

C7. How many years of experience do you have in teaching?
[__|__] year(s)

C8. Have you ever taught in other schools before you came to this school?
1. No **(SKIP TO C10)** 2. Yes

C9. Please write down in chronological order the beginning and ending year of schools you have worked in, the location and type of these schools, and whether they enroll students from non-local counties/districts?

	**Location of school:**<br><br>1. In a village<br>2. In a town<br>3. In a county<br>4. In a prefecture-level city<br>5. In a capital city<br>6. In a municipality	**Type of school:**<br><br>1. Public school<br>2. Privately-run and state-subsidized school<br>3. Ordinary private school<br>4. Private school for migrant children	**Did it enroll students from non-local counties/districts?**<br><br>1. Yes<br>2. No
From [__|__|__|__]<br>to [__|__|__|__] (YYYY)	[___]	[___]	[___]
From [__|__|__|__]<br>to [__|__|__|__] (YYYY)	[___]	[___]	[___]

From [__|__|__|__]<br>to [__|__|__|__] (YYYY)	[___]	[___]	[___]
From [__|__|__|__]<br>to [__|__|__|__] (YYYY)	[___]	[___]	[___]

C10. When did you begin to teach in this school?
[__|__|__|__][__|__] ([YYYY][MM])

C11. Are you an institutionally registered teacher admitted by the government?
1. Yes, I became a registered teacher in the year [__|__|__|__](YYYY).
2. No, I'm not.

C12. Do you have a professional title in teaching? If yes, what is it?
0. No, I don't.
1. Level-C Teacher 2. Level-B Teacher 3. Level-A Teacher
4. Senior-Level Teacher 5. Principal Senior-Level Teacher

C13. Are you holding other concurrent administrative posts? If yes, what is it?
0. No, I'm not
1. Head of the grade
2. Head of the teaching & research group of a subject
3. Teaching director or deputy teaching director
4. Deputy principal
5. Principal

C14. Do you receive government subsidies?
1. Yes 2. No

C15. When did you first take the post of a homeroom teacher?
[__|__|__|__][__|__] ([YYYY] [MM])

C16. Which of the following grades have you ever taught? (Please mark all that apply.)
1. The seventh grade 2. The eighth grade 3. The ninth grade
4. Elementary school grades 5. Senior high school grades

C17.Which of the following awards have you ever won because of your teaching performance? (Please mark all that apply.)
1. National award 2. Province-level award 3. City-level award
4. County-level award 5. School-level award 6. None

C18. Have you ever won the title of Excellent Homeroom Teacher over the past three years?
1. Yes, more than once 2. Yes, I got the title once
3. No 4. There is no such title in this school's evaluation system

C19. In general, are you satisfied with your current job as a homeroom teacher?
1. Strongly unsatisfied 2. Somewhat unsatisfied 3. Moderate
4. Somewhat satisfied 5. Strongly satisfied

C20. To what extent are you willing to take the post again as a homeroom teacher after you finished teaching this class?

1. Strongly unwilling to 2. Somewhat unwilling to 3. I don't care
4. Somewhat willing to 5. Strongly willing to

C21. How many close friends do you have among your colleagues in this school?

1. None 2. Just a few 3. Some 4. Many

C22. Have you ever feel tired of being a teacher?

1. Never 2. Seldom 3. Sometimes 4. Often

C23. Are you still willing to choose teacher as your job if you have a second chance to make a choice?

1. No 2. Not sure 3. Definitely yes

C24. Are you still willing to become a junior-high teacher if you have a second chance to make a choice?

1. No 2. Not sure 3. Definitely yes

C25. Are you still willing to teach in this school if you have a second chance to choose an institution to work for?

1. No 2. Not sure 3. Definitely yes

C26. Are you satisfied with each of the following aspects of this school?

	Strongly unsatisfied	Somewhat unsatisfied	Moderate	Somewhat satisfied	Strongly satisfied
Salary	1	2	3	4	5
School management	1	2	3	4	5
Facility	1	2	3	4	5
Students' quality	1	2	3	4	5

C27. What do you think of the school's academic atmosphere?

1. Very bad 2. Bad 3. Moderate 4. Good 5. Very good

C28. What do you think of the school's management of students?

1. Very loose 2.Loose 3. Moderate 4.Strict 5. Very strict

C29. In your opinion, what are the capabilities or qualities of students that the school needs to improve its educating further? (Please mark all that apply.)

1. The capability of analyzing and solving problems
2. Creativity and imagination
3. Self-discipline and perseverance
4. Positive values and morality
5. Interpersonal relationship, communication and cooperation
6. Mental health

7. Concerns for and engagement in public affairs

C30. In what aspects do you think the school should bear more responsibility than students' family? (Please mark all that apply.)

1. The capability of analyzing and solving problems
2. Creativity and imagination
3. Self-discipline and perseverance
4. Positive values and morality
5. Interpersonal relationship, communication and cooperation
6. Mental health
7. Concerns for and engagement in public affairs

C31. What do you think is the most important goal of school education? (Please mark all that apply.)

1. To foster virtue
2. To pass on basic knowledge
3. To foster comprehensive capabilities
4. To increase senior high school/college/university acceptance rate
5. To make sure students can grow up happily and safely

Part D: Contact Information

We hope to continue the tracking of the students' future progress through this panel survey. Please leave your contact information so that we could keep in touch with you and your students:

Your office phone number: ______________

Your mobile phone number: _____________

Your Email address: ___________________

Your QQ number: _____________________

This is the end of the questionnaire. Thank you for your engagement and cooperation! We wish you a good health and successful career!

Name of School Administrator:______________

Name of School:___________________

Location of School: County/District: ______________City: ____________

Province (Autonomous Region/Municipality):____________

China Education Panel Survey

Academic Year 2013-2014

School Administrator Questionnaire

School administrators answering this questionnaire do not need to fill in the blanks in the box below.

1. Code of Questionnaire: [____|____|____|____|____|____]

2. Code of County/District: [____|____|____|____|____|____]

3. Verifier: ____________________

Distinguished school administrators,

China Education Panel Survey (CEPS) is the first large-scale, nationwide, and longitudinal social survey of junior high students in China. The purpose of the CEPS is to provide detailed and reliable data for the research of the current situation and future development of China's primary education.

CEPS is jointly conducted by the National Survey Research Center (NSRC) at Renmin University of China and academic institutions throughout the country. During the academic year of 2013-2014, we are going to survey nearly 20,000 students as well as their parents, homeroom teachers, subject teachers and school administrators. Through scientific sampling, your school has been chosen as the sample school. We would appreciate it very much if you could help us finish this questionnaire. Thank you for your understanding and support.

There are neither right nor wrong answers to these questions and your answers will not be evaluated as part of your work performance or that of your school. Therefore, please answer all the questions in this questionnaire truthfully based on your own ideas and the reality of the school. We pledge here that under *the Statistics Law of the People's Republic of China*, we will hold all the information you are going to provide in strict confidence and it will never be given away to any individual or institution. Thank you for your cooperation.

When answering a multiple-choice question, please circle the number of your choice; when answering a blank-filling question, please write down words or numbers on the lines in the square brackets.

National Survey Research Center (NSRC), Renmin University of China

March, 2014

Part A: Basic Information of the School

A1. Which category best describes your school:

1. Public school
2. Private school subsidized by the government
3. Ordinary private school
4. Private school for children of migrant workers →
5. Other (Please specify: __________)

> **Have you acquired the license from the government to run the school?**
> 1. Yes, we got it in the year [__|__|__|__]
> 2. Not yet, but we have got governmental support.
> 3. Not yet, and we are not supported by the government

A2. The higher competent administrative authority of your school is:

1. Bureau of Education at Prefecture level
2. Bureau of Education at County/District level
3. Other (Please specify: __________)

A3. Does the junior high department of your school enroll students from non-local counties/ districts?

1. Yes, starting from the year [__|__|__|__] 2. No

A4. Judging from the conditions of running schools, what is the current ranking of the junior high department of your school in the local county/district?

1. Near the bottom
2. Below average
3. Average
4. Above average
5. Among the best

A5. Compared to five years ago, how is the current ranking of the junior high department of your school in the local county/district? (If your school history or your tenure is currently less than five years, please answer according to the oldest information you can get.)

1. Lower 2. Not changed much 3. Higher

A6. When was your school founded?

In the year [__|__|__|__]

A7. In the recent five years, has your school been moved, expanded or reconstructed?

1. Yes 2. No

A8. In the recent five years, has your school been merged with other schools?

1. Yes 2. No

A9. Does your school have a playground with circular lanes?

1. Yes, we have lanes of [__|__|__|__] meters 2. No

A10. Does your school offer students' dormitories?

1. Yes, all the students live in school
2. Yes, some students live in school
3. No

A11. How many dormitory rooms are there in your school? (Please fill in 0000 if the school does not provide dormitories.)

[__|__|__|__] rooms; [__|__] persons/room on average

A12. Does your school have the following facilities:

	No	Yes, but need to be improved	Yes, and well equipped
Laboratory	1	2	3
Computer room	1	2	3
Library	1	2	3
Music room	1	2	3
Student activity room	1	2	3
Psychological counseling room	1	2	3
Student cafeteria	1	2	3
Playground	1	2	3
Gymnasium	1	2	3
Swimming pool	1	2	3

A13. How many classrooms are there in your school at present?

[____|____|____] classrooms in total

A14. How many seats on average are there in each classroom?

[____|____|____] seats

A15. Has your school realized *the Class Access to ICTs*? (*the Class Access to ICTs* means that electronic teaching facilities, such as computers, Internet and projectors, have been installed in the classrooms.)

1. None of the classrooms has 2.Some classrooms have

3. Most classrooms have 4.All the classrooms have

A16. How many computers does your school provide for students to use?

[___|___|___|___] (Please fill in 0000 if no computer is provided.)

A17. How many books are in your school's library/reading room?

[___|___|___|___|___|___] volumes

【000000】The school has a library/reading room, but there is no book in it.

【999999】The school has no library/reading room.

A18. How much fiscal appropriation per student has your school received this year? (including funding from both central and local government)

[____|____|____|____] yuan/student

(Please fill in 0000 if the school does not receive any funding from the government.)

A19. Are students from non-local counties/districts covered by the fiscal allocation as well?

1. Yes, and the quota is the same as that of the local students
2. Yes, but the quota is less than that of the local students
3. No
4. We do not enroll students from non-local counties/districts

A20. How is the standard of fiscal appropriation for junior high students this year compared with five years ago? (If your school history or your tenure is currently less than five years, please answer according to the oldest information you can get.)

1. Lower　　2. Not much changed　　3. Higher

A21. What is the percentage of each of the following items in your school's total funding sources?

	**Percentage**
Central or provincial-level fiscal appropriations	[____|____|____]%
Prefecture-level fiscal appropriations	[____|____|____]%
County/district-level fiscal appropriations	[____|____|____]%
Tuition and other charges from students	[____|____|____]%
Operational income like renting and contracting	[____|____|____]%
Public donations	[____|____|____]%
Other	[____|____|____]%
Total	100%

A22. At present, is your school a member of an educational group?

1. Yes, the name of the group: [_____]; the year of joining the group: [__|__|__|__] (YYYY)
2. No

A23. Which of the following best describes the community where the school is located:

1. Center of the city/town
2. Outskirts of the city/town
3. Rural-urban fringe zone of the city/town
4. Towns outside of the city/town
5. Rural areas
6. Other (Please specify: __________)

A24. What is the major type of communities surrounding your school?

1. Old urban areas without reconstruction (neighboring community)
2. Single or mixed unitary community
3. Affordable housing community
4. Ordinary commodity housing community
5. Exclusive residential district
6. "Urban village" (a village transformed into or merged with a community)
7. Rural areas
8. Other (Please specify: __________)

A25. Are there any of the following sites close to your school? (Please mark all that apply.)

1. Residential area
2. Cybercafé
3. Library
4. Canteen
5. Snack stands on the street
6. Public transport stations
7. Video arcades

A26. Does juvenile delinquency happen in the community where your school is located?

1. No 2. Seldom 3. Often 4.Always

A27. Compared to five years ago, how does the juvenile delinquency rate in your school's surrounding community change?

1. Lower 2. Not much changed 3. Higher

(Please continue to finish Part B.)

Part B: Basic Information of JUNIOR HIGH Students at School:

B1. The number of classes and students of each grade in your school is:

	Number of classes	Total number of students
Grade 7	[__|__]	[__|__|__|__]
Grade 8	[__|__]	[__|__|__|__]
Grade 9	[__|__]	[__|__|__|__]

B2. Compared to five years ago, how has the number of students in Grade 7 changed?

1. Decreased
2. Not changed much
3. Increased

B3. For students in Grade 7, what is the percentage of each of the following and how has it changed? (Please fill in 000 if you don't have that kind of student.)

	Percentage	Compared to five years ago:<br>1.Decreased<br>2.Not changed much<br>3.Increased
Students from the local county/district	[__|__|__]%	[__]
Students from non-local counties/districts in the province	[__|__|__]%	[__]
Students from other provinces	[__|__|__]%	[__]
Total	100%	

B4. What do you think of the quality of students enrolled over the past five years?

1. Increasingly worse
2. Almost the same
3. Increasingly better

B5. Compared to students from the local county/district, how do students from non-local counties/districts perform in the following subjects when entering school?

	Much poorer	Somewhat poorer	About the same	Somewhat better	Much better	No students in this school are from non-local counties/districts
Math	1	2	3	4	5	9
Chinese	1	2	3	4	5	9
English	1	2	3	4	5	9

B6. Compared to students from the local county/district, what do you think of the behaviors and habits of students from non-local counties/districts when entering your school?

1. Worse than students from the local county/district
2. About the same
3. Better than students from the local county/district
4. No students in this school are from non-local counties/districts

B7. Compared to students from the local county/district, what do you think of the learning attitude of students from non-local counties/districts when entering your school?

1. Not as hardworking as students from the local county/district
2. Almost the same
3. More hardworking than students from the local county/district
4. No students in this school are from non-local counties/districts

B8. On average, what is the educational level of the students' parents in your school compared to other schools in the local county/district?

1. Near the bottom
2. Below average
3. Average
4. Above average
5. Among the highest

B9. On average, what is the level of income of the students' parents in your school in the local county/district?

1. Near the bottom
2. Below average
3. Average
4. Above average
5. Among the highest

B10. What are the top 3 professions of the students' parents in your school? Please fill in the following blanks with numbers above:

1. Peasants
2. Workers
3. Small business owners
4. Intellectuals
5. Managers/administrators of enterprises/corporations
6. Governmental officials/civil servants

Top 1st: [__]
Top 2nd: [__]
Top 3rd: [__]

B11. What are the top 3 professions of the NON-LOCAL students' parents in your school? Please fill in the following blanks with numbers in Question B10. If no students in this school are from non-local counties/districts, please fill in "9" in each of the following blank.

Top 1st: [__]
Top 2nd: [__]
Top 3rd: [__]

B12. What are the differences between parents from the local county/district and non-local counties/districts in the following situations?

	Those from the local county/district do better than those from non-local counties/districts	About the same	Those from the local county/district do worse than those from non-local counties/districts	No students in this school are from non-local counties/districts
Concern about child's academic record	1	2	3	9
Concern about child's daily life	1	2	3	9
Expectation on child's education	1	2	3	9
Management of child's behaviors and habits	1	2	3	9
Communication with school teachers	1	2	3	9
Cooperation with the school	1	2	3	9

B13. In the last academic year (2012-2013), how many students in your school have dropped out, transferred out of or into the school? (Please fill in 000 if no such student.)

	Drop out	Transfer out	Transfer in
Grade 7	[__|__|__]	[__|__|__]	[__|__|__]
Grade 8	[__|__|__]	[__|__|__]	[__|__|__]
Grade 9	[__|__|__]	[__|__|__]	[__|__|__]

B14. Does your school worry about the following situations that will possibly happen in five years?

	Very much worried	Somewhat worried	Not very worried	Not worried at all
Increasing number of students	1	2	3	4
Difficulty to enroll students	1	2	3	4
Student loss	1	2	3	4
Teacher loss	1	2	3	4
Dismantlement and merging of the school	1	2	3	4

(Please continue to finish Part C.)

Part C: Basic Information of Teachers in the JUNIOR HIGH Department

C1. What is the number of teachers in each of the following categories? (Please fill in 000 if there is no teacher in your school fit a particular category.)

	Number of teachers
Total number of teachers	[____|____|____]
Among them: the number of male teachers	[____|____|____]
The number of teachers who have a Teacher Certification	[____|____|____]
The number of substitute teachers	[____|____|____]
The number of teachers resigned or retired in the academic year 2012-2013	[____|____|____]
The number of newly hired teachers in the academic year 2012-2013	[____|____|____]
The students to teachers ratio five years ago	[____]:[____]
The students to teachers ratio at present	[____]:[____]

C2. Is it easy or difficult for your school to hire new teachers in each of the following subjects?

	Somewhat easy	Somewhat difficult	Very difficult
Math	1	2	3
Chinese	1	2	3
English	1	2	3

C3. What is the number of teachers in your school with different number of years' teaching experience? (Please fill in 000 if no teacher in your school fit a particular category.)

	Number of teachers
1~4 years of teaching experience	[____|____|____]
5~9 years of teaching experience	[____|____|____]
Over 10 years of teaching experience	[____|____|____]

C4. What is the number of teachers in your school having the following educational background?
(Please fill in 000 if no teacher in your school fit a particular category.)

	Total number of teachers	Number of teachers graduated from normal colleges or pedagogical majors:
Junior high school degree or below	[____|____|____]	(none)
Senior high school/technical secondary school/vocational high school degree	[____|____|____]	(none)
Junior college degree	[____|____|____]	[____|____|____]
Bachelor degree	[____|____|____]	[____|____|____]
Master degree	[____|____|____]	[____|____|____]

C5. What is the number of teachers in your school having the following professional titles?
(Please fill in 000 if no teacher in your school fit a particular category.)

	Number of teachers
Not rated	[____|____|____]
Level-C teacher	[____|____|____]
Level-B teacher	[____|____|____]
Level-A teacher	[____|____|____]
Senior-level teacher	[____|____|____]
Principal senior-level teacher	[____|____|____]

C6. What is the annual average income of the following types of teachers in your school?

	Annual average income
Senior-level teachers	[____|____|____|____|____|____|____] *yuan*
Teachers with a teaching experience of over 10 years	[____|____|____|____|____|____|____] *yuan*

C7. At present, does your school hire teachers from all over the country?

1. Yes, from the year [__|__|__|__] (YYYY) 2. No

C8. What is the minimum standard of educational background when hiring new teachers?

1. Junior high school degree or below
2. Senior high school/technical secondary school/vocational high school degree
3. Junior college degree
4. Bachelor degree
5. Master degree

C9. Is a teacher certification required when applying for the teaching posts in your school?

1. Yes 2. No

C10. Does your school offer official teachers' establishment when hiring new teachers?

1. Yes 2. No

C11. In which of the following subjects has your school ever lowered the employment standards because of the shortage of teachers? (Please mark all that apply.)

1. Chinese
2. Math
3. English
4. Other (Please specify: __________)
5. No, we have never done that

C12. Does your school provide training programs for teachers? (including all kinds of training programs organized by your school, the local education commission or the local education bureau)

1. Yes, we had [__|__] training courses in total last year
2. No

C13. Is the salary of teachers in your school subsidized by the government?

1. Yes 2. No

(Please continue to finish Part D.)

Part D: Enrollment of Students in the JUNIOR HIGH Department

D1. Is the student enrollment procedure of your school independent or assigned by the governmental quotas?

1. Independent and open to the public (or within the school district)
2. Assigned by the government and not open to the public
3. Both of the above
4. Other (Please specify: __________)

D2. What are the channels for parents to know about the application procedure of your school? (Please mark all that apply.)

1. Primary schools where their children have graduated from
2. Street offices/township governments
3. The Internet
4. Information offered by the school
5. Other parents
6. Other (Please specify: __________)

D3. Which of the following qualifications are required for a student to be enrolled by your school? (Please mark all that apply.)

1. Hukou1 booklet
2. Property ownership certificate or lease contract
3. Temporary residential permit or residence permit
4. Contributing to social security for a year
5. Family planning certificate
6. Business license or employment certificate
7. "Credits" that meet the conditions
8. Other (Please specify: __________)

D4. Do you think it is difficult to obtain the required qualification(s) above?

0. Not difficult
1. Yes, and the most difficult to obtain is [__] (Please fill in with the number in Question D3.)

D5. Have students from non-local counties/districts met the required qualifications for enrollment?

1. All of them have
2. Some of them have
3. No qualification is required
4. No students in this school are from non-local counties/districts

D6. At present, are students required to pass an entrance examination or an interview to be enrolled by your school?

1. Yes 2. No

[1] Hukou refers to a record of household registration system in China that identifies a person as a resident of an area.

D7. How much does a junior high student have to pay for each of the following items IN EACH SEMESTER? (Please fill in 0000 if there is no charge for a particular item.)

	Students from the local county/district	Students from non-local counties/districts	No students in this school are from non-local counties/districts
Tuition fees	[__|__|__|__] *yuan*	[__|__|__|__] *yuan*	9
Book fees	[__|__|__|__] *yuan*	[__|__|__|__] *yuan*	9
Activity fees	[__|__|__|__] *yuan*	[__|__|__|__] *yuan*	9
Meals Charge	[__|__|__|__] *yuan*	[__|__|__|__] *yuan*	9
Boarding fees	[__|__|__|__] *yuan*	[__|__|__|__] *yuan*	9
Other (Please specify: _____)	[__|__|__|__] *yuan*	[__|__|__|__] *yuan*	9

D8. In recent years, are students from the LOCAL county/district offered the following governmental subsidies in education? (Please mark all that apply.)

1. Exemption of book fees, starting from the year [___|___|___|___]
2. Free lunch offered by the school, starting from the year [___|___|___|___]
3. Grants for poor students, starting from the year [___|___|___|___]
4. Other (Please specify: __________), starting from the year [___|___|___|___]
5. No subsidies
6. No students in this school are from the local county/district

D9. In recent years, do students from the NON-LOCAL counties/districts offered the following governmental subsidy in education? (Please mark all that apply.)

1. Exemption of book fees, starting from the year [___|___|___|___]
2. Free lunch offered by the school, starting from the year [___|___|___|___]
3. Grants for poor students, starting from the year [___|___|___|___]
4. Other (Please specify: __________), starting from the year [___|___|___|___]
5. No subsidies
6. No students in this school are from the non-local counties/districts

(Please continue to finish Part E.)

Part E: Management of the JUNIOR HIGH Department

E1. Generally speaking, how many days are students required to attend school every week in your school? (1 decimal digit is reserved.)

Grade 7: [__].[__]days

Grade 9: [__].[__]days

E2. At what time are students required to arrive and leave school every day? (24-hour time system)

	Time required to arrive at school	Time required to leave school
Grade 7	[__|__]: [__|__]	[__|__]: [__|__]
Grade 9	[__|__]: [__|__]	[__|__]: [__|__]

E3. How many classes are students required to take every day and how long does each class last?

	Number of classes everyday	Lasting period of each class
Grade 7	[__|__]	[__|__] minutes
Grade 9	[__|__]	[__|__] minutes

E4. Does your school offer classrooms for students to self-study at night?

1. Yes 2. No **(SKIP TO E8)**

E5. Does your school require students in Grade 7 and Grade 9 to self-study at night in school?

1. Yes, we require both of the two grades to self-study at night in school
2. Yes, we require only students in Grade 9 to self-study at night in school
3. No **(SKIP TO E8)**

E6. The time arrangement of self-studying at night: (24-hour time system)

	Starting time	Ending time
Grade 7	[__|__]: [__|__]	[__|__]: [__|__]
Grade 9	[__|__]: [__|__]	[__|__]: [__|__]

E7. Will there be teachers in classroom to monitor students' self-study at night in school?

1. Yes 2. No

E8. Does your school organize the following activities in the summer or winter vacation? (Please mark all that apply.)

0. No
1. Cram classes
2. Advanced classes
3. Summer/winter camps
4. Other (Please specify: __________)

E9. Does your school fit in each of the following descriptions?

	Not fit at all	Somewhat not fit	Somewhat fit	Exactly fit
Parents do not cooperate with the school	1	2	3	4
Parents demand their children to be in specific classes or be taught by specific teachers	1	2	3	4
Students often skip school.	1	2	3	4
It is hard to manage the school discipline.	1	2	3	4
The school is crowded.	1	2	3	4
The flow of teachers is frequent.	1	2	3	4

E10. How many students can you name in your school? (Please fill in the percentage)
[__|__|__]%

E11. How often did each of the following happen last week?

	Never	1-4 times	5-10 times	Over 10 times
Students' fight with each other	1	2	3	4
Students' vandalism	1	2	3	4
Students' smoking	1	2	3	4
Students' drinking	1	2	3	4
Gang activities within or outside the campus	1	2	3	4
Chaotic classroom atmosphere	1	2	3	4
Teachers scolding at students	1	2	3	4
Corporal punishment of students by teachers	1	2	3	4

E12. If students from non-local counties/districts FROM OTHER PROVINCES are transferred to another school or drop out, how will their school rolls/student files be handled?

1. Transfer them to the new school
2. Transfer them to its province-level educational departments
3. Offer them a school transfer certificate
4. Other (Please specify: __________)

E13. Does your school accept transferred-in students (from other schools)?

1. No
2. Only accepted for Grade 7
3. Only accepted for Grade 7 and Grade 8
4. Yes, accepted for all grades

E14. How does your school manage the school rolls/student files of transferred-in students FROM OTHER PROVINCES?

1. To continue the records and files transferred from the previous school
2. To create new records and files
3. Other (Please specify: __________)

E15. What are the standards for arranging classes for new students? (Please mark all that apply.)

1. Grades they have got in the entrance exam
2. Location of their hukou
3. Randomly or evenly assign them into different classes
4. Other

E16. Will your school rearrange the students in Grade 8/9 into different classes at the beginning of the semester?

1. Yes
2. No

E17. This rearrangement of classes is based on:

1. Chinese scores
2. Math scores
3. English scores
4. Total scores
5. Not based on academic records

E18. What are the major teaching methods used by teachers in your school? (Please mark all that apply.)

1. Lectures
2. Group discussion
3. Bilingual teaching
4. Students choosing classes in different levels according to their own capabilities
5. Other (Please specify: __________)

E19. What measure will be taken by the school if students fail to pass exams?

1. Requiring them to repeat a year
2. After-class tutoring
3. Other (Please specify: __________)
4. No particular measure

E20. For students performing outstandingly well in a certain subject, does your school offer opportunities for further improvement?

1. Yes
2. No

E21. On average, how many classes does a major subject (math, Chinese or English) teacher (not a homeroom one) teach?

[___|___] class(s) in total

E22. Is it difficult for your school to arrange teachers in the following subjects?

	Not difficult	Somewhat difficult	Very difficult
Math	1	2	3
Chinese	1	2	3
English	1	2	3

E23. At present, what are the challenges that your school is facing with in management? (Please mark all that apply.)

1. Poor quality of students enrolled
2. Too many students from non-local counties/districts
3. Poor quality of teachers
4. Lack of funding
5. Other (Please specify: __________)
6. None of the above

E24. During the last semester, how many times did your school do each of the following?

	Never	1 time	2-4 times	Over 5 times
Parent meeting	1	2	3	4
Writing reports to parents about students' performance at school	1	2	3	4
Inviting parents to attend classes	1	2	3	4
Inviting parents to have an informal discussion with teachers	1	2	3	4
Inviting parents to watch students' shows or to take part in extracurricular activities	1	2	3	4
Holding lectures for students on daily-life guidance like emotional management and parent-child relationship	1	2	3	4

E25. In the 2012-2013 academic year, did your school hold the following activities targeted at the parents of students in Grade 9? (Please mark all that apply.)

1. Seminars on further education
2. Career lectures and educational counseling seminars
3. None

E26. In the 2012-2013 academic year, how many students have graduated from the junior high department in your school? (Please circle "9" if no students in this school are from non-local counties/districts.)

	Students from the local county/district	Students from non-local counties/districts	No students in this school are from non-local counties/districts
Total number of graduates in the junior high department	[__|__|__|__]	[__|__|__|__]	9
Number of students admitted to key senior high schools	[__|__|__|__]	[__|__|__|__]	9
Number of students admitted to regular senior high schools	[__|__|__|__]	[__|__|__|__]	9
Number of students admitted to vocational high schools/technical secondary schools /technical schools	[__|__|__|__]	[__|__|__|__]	9
Number of students entering labor market	[__|__|__|__]	[__|__|__|__]	9

E27. Are students from non-local counties/districts allowed to take the high school entrance examination in the local county/district?

1. Yes, starting from the year [__|__|__|__]
2. No
3. No students in this school are from non-local counties/districts

E28. As far as you know, are students from non-local counties/districts allowed to apply for each of the following types of senior high schools in this prefecture-level city?

	No	Yes, but there are special requirements	Yes, and no special requirements	Not clear	No students in this school are from non-local counties/districts
Key senior high schools	1	2	3	4	9
Regular senior high schools	1	2	3	4	9
Vocational high schools/technical secondary schools /technical schools	1	2	3	4	9

(Please continue to finish Part F.)

Part F: Personal Information

F1. What is your administrative post in this school?
1. Principal
2. Vice-principal
3. Dean
4. Other (Please specify: __________)

F2. When did you begin to hold the post in this school?
In the year [__|__|__|__] ([YYYY])

F3. Your sex is:
1. Male 2. Female

F4. Your year of birth is: [__|__|__|__] ([YYYY])

F5. What is your political affiliation at present?
1. Member of the Communist Party
2. Member of a Democratic Party
3. The masses

F6. What is the highest education level you have completed or are working on at present?
1. Junior high school degree or below
2. Vocational high school/technical secondary school/technical school degree
3. Senior high school degree
4. Junior college degree
5. Bachelor degree (attained through adult higher education)
6. Bachelor degree (attained through regular higher education)
7. Master degree or higher

F7. Are you a graduate from normal universities or pedagogical majors?
1. Yes
2. No

F8. Do you have a teacher certification?
1. Yes, I obtained it in the year [__|__|__|__] 2. No

F9. Before taking the administrative post in this school, have you ever worked in an administrative department of education like the Education Bureau or the Education Commission?
1. Yes, I have worked in such department for [___|___] years
2. No

F10. Before taking the administrative post in this school, have you ever been a teacher at school?
1. Yes, I have been a teacher for [___|___] years
2. No

F11. How many years have you engaged yourself in education?

[____|____] years

F12. How many years have you worked in this school?

[____|____] years

F13. How many years have you ever served as head of schools? (including head of other schools)

[____|____] years

F14. In your opinion, what are the capabilities or qualities of students that the school needs to improve its educating further? (Please mark all that apply.)

1. The capability of analyzing and solving problems
2. Creativity and imagination
3. Self-discipline and perseverance
4. Positive values and morality
5. Interpersonal relationship, communication and cooperation
6. Mental health
7. Concerns for and engagement in public affairs

F15. In what aspects do you think the school should bear more responsibility than students' family? (Please mark all that apply.)

1. The capability of analyzing and solving problems
2. Creativity and imagination
3. Self-discipline and perseverance
4. Positive values and morality
5. Interpersonal relationship, communication and cooperation
6. Mental health
7. Concerns for and engagement in public affairs

F16. What do you think is the most important goal of school education? (Please mark all that apply.)

1. To foster virtue
2. To pass on basic knowledge
3. To foster comprehensive capabilities
4. To increase senior high school/college/university acceptance rate
5. To make sure students can grow up happily and safely

(Please continue to finish Part G.)

Part G: Contact Information

We hope to continue the tracking of the students' future progress and the development of your school through this panel survey. Please leave the contact information of the school principal and that of another person in charge so that we could keep in touch with your school and students:

G1. Contact information of the school principal

Name: ______________________________

Office phone number: ________________ Mobile phone number: _______________

E-mail: _____________________________ QQ: _______________________________

G2. Contact information of another person in charge

Name: ______________________________

Office phone number: ________________ Mobile phone number: _______________

E-mail: _____________________________ QQ: _______________________________

This is the end of the questionnaire. Thank you for your engagement and cooperation! We wish you a good health and a successful career!

China Education Panel Survey
Academic Year 2014-2015
Student Questionnaire for Grade 8

Class: __________

Student Name: __________

Name of Homeroom Teacher: __________

Province (Autonomous Region/Municipality): _______________

County (District): ___________ City: _______________

Name of School: ____

- -

Students answering this questionnaire do not need to fill in the blanks in the box below.

Code of Questionnaire: [____|____|____|____|____|____]

Code of County/District: [____|____|____|____|____|____]

Hi Dear Student,

China Education Panel Survey is the first large-scale, nationwide, and longitudinal social survey of junior high students in China. It is jointly conducted by the National Survey Research Center (NSRC) at Renmin University of China and academic institutions throughout the country. Last year, we surveyed nearly 20,000 students as well as their parents, homeroom teachers, subject teachers and school administrators; you are one of them and we appreciate your participation. This year, we hope all of you can fill in another questionnaire to help us understand your growth and progress you made during this period.

There are neither right nor wrong answers to these questions and your answers will not be evaluated as part of your academic record. We pledge here that under *the Statistics Law of the People's Republic of China*, we will hold all the information you are going to provide in strict confidence and it will never be given away to any individual or institution. Thank you for your cooperation.

When answering a multiple-choice question, please circle the number of your choice; when answering a blank-filling question, please write down words or numbers on the lines in the square brackets.

National Survey Research Center (NSRC), Renmin University of China

March, 2015

Part A: Personal Background

A1.The location of your Hukou[1] *AT PRESENT* is:

1. In the local county/district
2. Not in the local county/district: County/district: [_________], City: [_________], Province (Autonomous Region/Municipality): [_________]

A2.What is the type of your Hukou *AT PRESENT*? (Agricultural Hukou refers to rural Hukou, a record that identifies a person as a rural resident. Non-agricultural Hukou refers to urban Hukou. Residential Hukou is a general record of residence assigned to all residents of a certain region, regardless of rural or urban background)

1. Agricultural Hukou 2. Non-agricultural Hukou
3. Residential Hukou 4. I have no Hukou

A3.Where is your home *AT PRESENT*? (Home refers to the place you go back to after school every day, or at weekends if you live in a boarding school)

1. In the local county/district 2. Not in the local county/district

A4. What is the level of your command of local dialect?

[1] Hukou refers to a record of household registration system in China that identifies a person as a resident of an area.

1. Don't understand at all
2. Can understand when listening, but cannot speak it
3. Can speak a little bit
4. Can speak mediocre local dialect
5. Can speak fluently

A5. Are you the only child of your family?

1. Yes, I am. **(SKIP TO A7)**
2. No, I am not.

A6.How many *FULL OR HALF* siblings do you have *AT PRESENT*? (Please fill in 0 if you don't have any sibling.)

[___] elder brother(s)
[___] younger brother(s)
[___] elder sister(s)
[___] younger sister(s)

A7.Which of the following people live in the same household with you *AT PRESENT*? (Please mark all that apply.)

1. Biological father
2. Biological mother
3. Stepfather
4. Stepmother
5. Full or half sibling(s)
6. Grandparent(s) on mother's/father's side
7. Other relative(s)
8. Other non-relative(s)

A8.What's the *CURRENT* marital status of your biological parents? (Please mark all that apply.)

1. Married → **Do they live together?**
   1. Yes, they do. 2. No, they don't.

2. Divorced → **a. When did they divorce?**
   In the year of [___|___|___|___]

   b. To whom did the court grant your custody?
   1. Father 2. Mother
   3. Other people (Please specify____________)

   c. Have they remarried another person after getting divorced? (Please mark all that apply)
   1. Father has remarried.
   2. Mother has remarried.
   3. Neither of them has remarried.

3. My father has passed away. → **Has your mother remarried ever since?**
   1. Yes, she has. 2. No, she hasn't.

4. My mother has passed away. → **Has your father remarried ever since?**
   1. Yes, he has. 2. No, he hasn't.

A9.Which one of the following do you think best describes the financial conditions of your family *AT PRESENT*?

1. Very poor 2. Somewhat poor 3. Moderate 4. Somewhat rich 5. Very rich

A10.Do you have a writing desk of your own at home?

1. Yes, I do. 2. No, I don't.

A11.How many books do your family own? (not including textbooks or magazines)

1. Very few 2. Not many 3. Some
4. Quite a few 5. A great number

A12.Do your family own a computer and have an access to the Internet?

0. No, we don't.
1. We have a computer but no access to the Internet.
2. Yes, we have both.

A13.What's the *CURRENT* occupation of your father?

1. Government official/cadre
2. Cadre/Official/Administrator of public institutions, enterprises/corporations
3. Scientist, engineer, university professor or other professionals
4. Doctor, lawyer, high school/primary school teacher
5. Accountant, nurse, computer programmer or other technical staff
6. Ordinary staff or worker (for example: secretary, bank clerk, or librarian)
7. Ordinary staff or worker in business or service field (for example: salesperson, agent, cook, barber, or cosmetologist)
8. Technical worker (for example: driver, electrician, plumber, or mechanist)
9. Ordinary worker (for example: porter, or production line worker)
10. Farmer, herdsman, fisherman
11. Elementary worker (for example: cleaner, guard, housekeeper, or sanitation worker)
12. Self-employed worker
13. Unemployed or laid-off worker
14. Other (Please specify_________)

A14. What's the *CURRENT* occupation of your mother?

1. Government official/cadre
2. Cadre/Official/Administrator of public institutions, enterprises/corporations
3. Scientist, engineer, university professor or other professionals
4. Doctor, lawyer, high school/primary school teacher
5. Accountant, nurse, computer programmer or other technical staff
6. Ordinary staff or worker (for example: secretary, bank clerk, or librarian)
7. Ordinary staff or worker in business or service field (for example: salesperson, agent, cook, barber, or cosmetologist)
8. Technical worker (for example: driver, electrician, plumber, or mechanist)
9. Ordinary worker (for example: porter, or production line worker)
10. Farmer, herdsman, fisherman
11. Elementary worker (for example: cleaner, guard, housekeeper, or sanitation worker)
12. Self-employed worker
13. Unemployed or laid-off worker
14. Other (Please specify_________)

A15.Does your father often get drunk?

1. Yes, he does. 2. No, he doesn't.

A16. Do your parents quarrel a lot?

1. Yes, they do. 2. No, they don't.

A17.Do your parents get along very well?

1. Yes, they do. 2. No, they don’t.

A18.How often did your parents check up on your homework *LAST WEEK*?

1. Never 2. One or two days 3. Three or four days 4. Almost everyday

A19.How often did your parents give instruction on your homework *LAST WEEK*?

1. Never 2. One or two days 3. Three or four days 4. Almost everyday

A20.Do your parents care and are they strict with you about the following?

	They don't care.	They do care about it, but are not strict.	They are very strict about it.
Your homework and examination	1	2	3
Your behavior at school	1	2	3
Whom you make friends with	1	2	3
Your dress style	1	2	3
Time you spend on the Internet	1	2	3
Time you spend on watching TV	1	2	3

A21.How often do your parents discuss the following with you?

	Your father			Your mother		
	Never	Sometimes	Often	Never	Sometimes	Often
Things happened at school	1	2	3	1	2	3
The relationship between you and your friends	1	2	3	1	2	3
The relationship between you and your teachers	1	2	3	1	2	3
Your worries and troubles	1	2	3	1	2	3

A22.How is the general relationship between you and your father?

1. Not close 2.Not too close nor too far 3.Very close

A23.How is the general relationship between you and your mother?

1. Not close 2.Not too close nor too far 3.Very close

A24.How often do you have dinner with your parents?

1. Never 2.Once a year 3.Once every half year
4. Once a month 5.Once a week 6.More than once a week

A25. How often do you visit museums, zoos, science museums, etc. with your parents?

1. Never 2.Once a year 3.Once every half year

4. Once a month | 5.Once a week | 6.More than once a week

A26.How often do you go out to watch movies, shows, sports games, etc. with your parents?

1. Never | 2.Once a year | 3.Once every half year
4. Once a month | 5.Once a week | 6.More than once a week

A27.What is your parents' requirement on your academic record?

1. Being one of the top five of your class
2. Above the average
3. About the average
4. No special requirement

A28.What is the highest level of education your parents expect you to receive?

1. Drop out now
2. Graduate from junior high school
3. Go to technical secondary school or technical school
4. Go to vocational high school
5. Go to senior high school
6. Graduate from junior college
7. Get a bachelor degree
8. Get a Master degree
9. Get a Doctor degree
10. They don’t care

A29.How do you feel about such expectation?

1. Not stressed at all | 2. Not very stressed | 3. Normal
4. Somewhat stressed | 5. Very stressed

A30. What kind of job do your parents *MOST* expect you to do in the future?

1. Government official, staff of public institutions, civil servant
2. Manager or administrator of enterprises/corporations
3. Scientist/engineer/doctor/programmer/pilot/spaceman
4. Teacher/lawyer/accountant/translator
5. Professional designer (such as costume, gardening, or advertisement designer)
6. Artistic performer (including writer/drawer/host/director/screenwriter)
7. Professional athlete
8. Technical worker (including driver/cook/maintenance staff)
9. Soldier/policeman
10. Medium service staff (including stewardess/nurse/barber/cosmetologist), or ordinary office staff
11. Self-employed (such as opening a store)
12. Other (Please specify: __________)
13. They don’t care
14. Not clear

A31.Where do you think your parents *MOST* expect you to live and work in the future?

1. In rural area
2. In towns/counties
3. In small or medium cities
4. In capital cities

5. In Beijing/Shanghai/Guangzhou
6. Abroad
7. They don’t care
8. Not clear

A32.Are your parents confident about your future?

1. Not confident at all
2. Not so confident
3. Somewhat confident
4. Very confident

(Please continue to finish Part B.)

Part B: Academic Development

B1.How many students were there in your class when you were in *GRADE 6*?

[___] students

B2.*AT PRESENT*, is mathematics difficult for you?

1. Very difficult 2. A bit difficult 3. Not very difficult 4. Not difficult at all

B3.*AT PRESENT*, is Chinese difficult for you?

1. Very difficult 2. A bit difficult 3. Not very difficult 4. Not difficult at all

B4.*AT PRESENT*, is English difficult for you?

1. Very difficult 2. A bit difficult 3. Not very difficult 4. Not difficult at all

B5. How much do you agree with each of the following statements about the main subjects?

	**Strongly disagree**	**Somewhat disagree**	**Somewhat agree**	**Strongly agree**
My mathematics teacher always pays attention to me.	1	2	3	4
My Chinese teacher always pays attention to me.	1	2	3	4
My English teacher always pays attention to me.	1	2	3	4
My mathematics teacher always asks me to answer questions in class.	1	2	3	4
My Chinese teacher always asks me to answer questions in class.	1	2	3	4
My English teacher always asks me to answer questions in class.	1	2	3	4
My mathematics teacher always praises me.	1	2	3	4
My Chinese teacher always praises me.	1	2	3	4
My English teacher always praises me.	1	2	3	4

B6. How much do you agree with each of the following statements about your school life?

	**Strongly disagree**	**Somewhat disagree**	**Somewhat agree**	**Strongly agree**
My parents always receive praises on me from my teacher.	1	2	3	4
My parents always receive criticism on me from my teacher.	1	2	3	4
My homeroom teacher always praises me.	1	2	3	4
My homeroom teacher always criticizes me.	1	2	3	4
Most of my classmates are nice to me.	1	2	3	4
My class is in good atmosphere.	1	2	3	4

	Strongly disagree	Somewhat disagree	Somewhat agree	Strongly agree
I often take part in school/class activities.	1	2	3	4
I feel close to people in this school.	1	2	3	4
I feel bored in this school.	1	2	3	4
I hope that I could transfer to another school.	1	2	3	4

B7.How much time *ON AVERAGE EVERYDAY* did you spend on the following extra-curricular activities *FROM MONDAY TO FRIDAY*?

**Doing homework assigned by teacher:**		
1. 0 hour	2. Less than 1 hour	3. About 1-2 hours
4. About 2-3 hours	5. About 3-4 hours	6. More than 4 hours
**Doing homework assigned by parents or cram school:**		
1. 0 hour	2. Less than 1 hour	3. About 1-2 hours
4. About 2-3 hours	5. About 3-4 hours	6. More than 4 hours
**Taking cram school courses (related to schoolwork):**		
1. 0 hour	2. Less than 1 hour	3. About 1-2 hours
4. About 2-3 hours	5. About 3-4 hours	6. More than 4 hours
**Taking interest related courses (not related to schoolwork):**		
1. 0 hour	2. Less than 1 hour	3. About 1-2 hours
4. About 2-3 hours	5. About 3-4 hours	6. More than 4 hours
**Watching TV:**		
1. 0 hour	2. Less than 1 hour	3. About 1-2 hours
4. About 2-3 hours	5. About 3-4 hours	6. More than 4 hours
**Surfing on the Internet or playing video games:**		
1. 0 hour	2. Less than 1 hour	3. About 1-2 hours
4. About 2-3 hours	5. About 3-4 hours	6. More than 4 hours

B8.How much time *ON AVERAGE EVERYDAY* did you spend on the following extra-curricular activities *ON WEEKENDS*?

**Doing homework assigned by teacher:**		
1. 0 hour	2. Less than 2 hour	3. About 2-4 hours
4. About 4-6 hours	5. About 6-8 hours	6. More than 8 hours

**Doing homework assigned by parents or cram school:**		
1. 0 hour	2. Less than 2 hour	3. About 2-4 hours
4. About 4-6 hours	5. About 6-8 hours	6. More than 8 hours
**Taking cram school courses (related to schoolwork):**		
1. 0 hour	2. Less than 2 hour	3. About 2-4 hours
4. About 4-6 hours	5. About 6-8 hours	6. More than 8 hours
**Taking interest related courses (not related to schoolwork):**		
1. 0 hour	2. Less than 2 hour	3. About 2-4 hours
4. About 4-6 hours	5. About 6-8 hours	6. More than 8 hours
**Watching TV:**		
1. 0 hour	2. Less than 2 hour	3. About 2-4 hours
4. About 4-6 hours	5. About 6-8 hours	6. More than 8 hours
**Surfing on the Internet or playing video games:**		
1. 0 hour	2. Less than 2 hour	3. About 2-4 hours
4. About 4-6 hours	5. About 6-8 hours	6. More than 8 hours

B9. How often did you visit museums, zoos, science museums, etc. either alone or with your schoolmates *IN THE PAST YEAR*?

1. Never 2. Once a year 3. Once every half year
4. Once a month 5. More than once a month 6. More than once a week

B10. How often did you go out to watch movies, shows, sports games, etc. either alone or with your schoolmates *IN THE PAST YEAR*?

1. Never 2. Once a year 3. Once every half year
4. Once a month 5. More than once a month 6. More than once a week

B11. What kind of extra-curricular courses do you take *IN THE PAST YEAR*? (Please mark all that apply.)

0. None
1. Mathematical Olympiad
2. Ordinary Mathematics (not including Mathematical Olympiad)
3. Chinese/Chinese Composition Writing
4. English
5. Painting or drawing
6. Calligraphy
7. Music/Musical instrument
8. Dancing
9. Chess
10. Sports

11. Other (Please specify: __________)

B12. What hobbies do you have? (Please mark all that apply.)

0. None
1. Playing musical instruments (Please specify: __________)
2. Vocal practices/Singing/Dancing/Acting
3. Calligraphy
4. Painting or drawing/Animation
5. Chess (Please specify: __________)
6. Sports (Please specify: __________)
7. Other (Please specify: __________)

B13.Besides the hobbies mentioned above (in question B12), what else do you often like to do? (Please mark all that apply.)

0. None
1. Reading books
2. Doing handcrafts
3. Spending time on mobile phones
4. Playing video games
5. Other (Please specify: ________)

B14.What did you usually do in winter and summer vacations *IN THE PAST YEAR*? (Please mark all that apply.)

1. Taking cram school courses (related to schoolwork)
2. Attending summer/winter camps
3. Helping parents out
4. Going back to hometown
5. Staying at home
6. Other (Please specify: __________)

B15. Do you live in school at night *FROM MONDAY TO THURSDAY*?

1. Yes, I do. 2. No, I don't.

B16. How do you usually go to school from home?

1. On foot
2. By bicycle
3. By electric mobile/electric power cart
4. By motorcycle
5. By city bus
6. By intercity bus
7. By private car
8. By train
9. By ship
10. By underground train
11. Other (Please specify: ________)

B17. Using the kind of transportation you chose in question B16, how long does it take from your home to school?

[___|___|___] minutes

B18. What is the highest level of education you expect yourself to receive?

1. Drop out now
2. Graduate from junior high school
3. Go to technical secondary school or technical school
4. Go to vocational high school
5. Go to senior high school
6. Graduate from junior college
7. Get a bachelor degree
8. Get a Master degree
9. Get a Doctor degree
10. I don’t care

B19. What kind of job do you *MOST* expect to do in the future?

1. Government official, staff of public institutions, civil servant
2. Manager or administrator of enterprises/corporations
3. Scientist/engineer/doctor/programmer/pilot/spaceman
4. Teacher/lawyer/accountant/translator
5. Professional designer (such as costume, gardening, or advertisement designer)
6. Artistic performer (including writer/drawer/host/director/screenwriter)
7. Professional athlete
8. Technical worker (including driver/cook/maintenance staff)
9. Soldier/policeman
10. Medium service staff (including stewardess/nurse/barber/cosmetologist), or ordinary office staff
11. Self-employed (such as opening a store)
12. Other (Please specify: __________)
13. I don’t care

B20.Where do you most expect to live and work when you grow up?

1. In rural area
2. In towns/counties
3. In small or medium cities
4. In capital cities
5. In Beijing/Shanghai/Guangzhou
6. Abroad
7. I don’t care

B21.Are you confident in your future?

1. Not confident at all
2. Not so confident
3. Somewhat confident
4. Very confident

(Please continue to finish Part C)

Part C: Physical and Mental Health

C1. Your *CURRENT* height is: [__|__|__] centimeters.

C2. Your *CURRENT* weight is: [__|__] kilograms (Please notice that the unit of the weight is 'kilogram' rather than 'jin')

C3. What do you think of your body shape?
1. Very thin 2. A bit thin 3. Not fat nor thin 4. A bit heavy 5. Very heavy

C4. Which one of the following best describes your general health condition *AT PRESENT*?
1. Very poor 2. Not very good 3. Moderate 4. Good 5. Very good

C5. How often did you get sick *IN THE PAST YEAR*? (such as getting a cold / fever / cough / diarrheal)
1. Never 2. Seldom 3. Often

C6. How many days have you been absent due to sickness *IN THE PAST YEAR*?

[__|__|__] days

C7. *UP TILL NOW,* have you ever had one of the following serious illnesses?

Kidney disease:	1. I don't know<br>2. No, I haven't<br>3. Yes, I had it when I was [__|__] years old and recovered when I was [__|__] years old (Fill in 99 if you haven't recovered yet)
Lung disease:	1. I don't know<br>2. No, I haven't<br>3. Yes, I had it when I was [__|__] years old and recovered when I was [__|__] years old (Fill in 99 if you haven't recovered yet)
Heart disease:	1. I don't know<br>2. No, I haven't<br>3. Yes, I had it when I was [__|__] years old and recovered when I was [__|__] years old (Fill in 99 if you haven't recovered yet)
Brain disease:	1. I don't know<br>2. No, I haven't<br>3. Yes, I had it when I was [__|__] years old and recovered when I was [__|__] years old (Fill in 99 if you haven't recovered yet)
Upper limb fracture:	1. I don't know<br>2. No, I haven't<br>3. Yes, I had it when I was [__|__] years old and recovered when I was [__|__] years old (Fill in 99 if you haven't recovered yet)
Lower limb fracture:	1. I don't know<br>2. No, I haven't<br>3. Yes, I had it when I was [__|__] years old and recovered when I was [__|__] years old (Fill in 99 if you haven't recovered yet)

C8. What was your eyesight according to your *LATEST* health examination at school?

1. I haven't received any health examination at school.
2. According to the result, my left eye is [__]. [__], and right eye is [__]. [__].

C9. Are you short-sighted?

1. Yes, I am.
   The strength of my left eyeglass is [__|__|__] degrees.
   The strength of my right eyeglass is [__|__|__] degrees.
2. Yes, I am. But I am not clear about the strength of my glasses/lens.
3. No, I am not. **(Skip to C11)**

C10. Are you born with myopia?

1. Yes, I am. 2. No, I'm not. 3. Not clear.

C11. Do you have dental caries / tooth decay?

1. Yes, I have [___] decayed tooth/teeth.
2. No, I have not. **(Skip to C13)**

C12. Have you ever had your tooth decay treated (such as filling a tooth)?

1. Yes, I have. 2. No, I haven't.

C13. How often do you do physical exercise?

Usually [___] days a week, [___|___|___] minutes a day.

C14. Your physical ability level:

For boys to answer: Pull-ups: [___|___] times
For girls to answer: Sit-ups: [___|___] times/minute.

C15. How often do you eat food such as fried food, barbecue, puffed food, or Western fast food?

1. Never 2. Seldom 3. Sometimes 4. Often 5. Always

C16. How often do you have sugary drinks (such as milk tea) or sodas (such as Coke)?

1. Never 2. Seldom 3. Sometimes 4. Often 5. Always

C17. Does any of your family members *WHO LIVE WITH YOU* smoke?

1. Yes. [____] of my family members do/does. 2. No, none of them does.

C18. How much time on average do you sleep *EVERY NIGHT*?

[__|__] hours [__|__] minutes

C19. Generally speaking, do you have any of the following sleeping problems? (Please mark all that apply)

0. I have no sleeping problems
1. Insomnia, difficult to fall asleep
2. Sleep fragmentation
3. Drowsiness
4. Remain fatigue after waking up
5. Snoring
6. Grind teeth in sleep
7. Sleepwalking
8. Dreaminess
9. Talk in sleep

C20. Have you ever taken any health education classes when you were in *ELEMENTARY SCHOOL?*

1. Yes, I have. 2. No, I haven't.

C21. Have you ever taken any health education classes when you were in *MIDDLE SCHOOL*?

1. Yes, I have. 2. No, I haven't.

C22. Your pubertal development:

For boys to answer: My first nocturnal emission occurred when I was [__|__] years old. (Fill in 99 if you haven't experienced one yet).

For girls to answer: My first menstrual period occurred when I was [__|__] years old. (Fill in 99 if you haven't experienced one yet).

C23. How do you view your appearance?

1. Very ugly 2. Somewhat ugly 3. Average-looking
4. Good-looking 5. Very good-looking

C24. How much do you agree with each of the following statements about your experiences in *GRADE 7*?

	Strongly disagree	Somewhat disagree	Somewhat agree	Strongly agree
I would try my best to go to school even if I was not feeling very well or I had other reasons to stay at home.	1	2	3	4
I would try my best to finish even the homework I dislike.	1	2	3	4
I would try my best to finish my homework, even if it would take me quite a long time.	1	2	3	4
I would persist in my interests and hobbies.	1	2	3	4

C25. Do you have the feelings below *IN THE LAST SEVEN DAYS*?

	Never	Seldom	Sometimes	Often	Always
Feeling blue	1	2	3	4	5
Too depressed to focus on anything	1	2	3	4	5
Unhappy	1	2	3	4	5
Not enjoying life	1	2	3	4	5
Having no passion to do anything	1	2	3	4	5
Sad, sorrowful	1	2	3	4	5
Nervous	1	2	3	4	5
Excessive worry	1	2	3	4	5

	Never	Seldom	Sometimes	Often	Always
Feeling something bad will happen	1	2	3	4	5
Too energetic to concentrate in class	1	2	3	4	5

C26. How much do you agree with the following statements?

	Strongly disagree	Somewhat disagree	Somewhat agree	Strongly agree
Usually I can recover from the feelings mentioned above very soon by myself.	1	2	3	4
Usually I can recover from the feelings mentioned above very soon with the help of other people.	1	2	3	4
If I have the feelings mentioned above, my teacher would ignore and isolate me.	1	2	3	4
If I have the feelings mentioned above, my teacher would criticize me in public.	1	2	3	4
If I have the feelings mentioned above, my teacher would criticize me privately.	1	2	3	4
If I have the feelings mentioned above, my teacher would try to help me.	1	2	3	4
If I have the feelings mentioned above, my teacher would ask my parents to help me together.	1	2	3	4

(Please continue to finish Part D)

Part D: Social Behaviors and Development

D1. How often did you do the following things *IN THE PAST YEAR?*

	Never	Seldom	Sometimes	Often	Always
Helping elders	1	2	3	4	5
Following orders and lining up	1	2	3	4	5
Being nice and honest	1	2	3	4	5

D2. How often did you do the following things *IN THE PAST YEAR*?

	Never	Seldom	Sometimes	Often	Always
Cursing or saying swearwords	1	2	3	4	5
Quarreling with others	1	2	3	4	5
Having a fight with others	1	2	3	4	5
Bullying the weak	1	2	3	4	5
Having a violent temper	1	2	3	4	5
Unable to concentrate on one thing	1	2	3	4	5
Skipping classes, being absent, or truanting	1	2	3	4	5
Copying homework from others, or cheating in exams	1	2	3	4	5
Smoking, or drinking alcohol	1	2	3	4	5
Going to net bars or video arcade	1	2	3	4	5

D3. How much do you agree with the following statements?

	Strongly disagree	Somewhat disagree	Somewhat agree	Strongly agree
I'm very shy.	1	2	3	4
Usually I would rather sit alone than join others.	1	2	3	4
I seldom talk and mostly listen to them when I'm with my schoolmates or friends.	1	2	3	4
There are some adults I respect and admire.	1	2	3	4
I can chat with adults easily.	1	2	3	4
I would apologize if I hurt others unintentionally.	1	2	3	4
I would try to find other ways to solve problems if my approach to dealing with things is inappropriate.	1	2	3	4
I can stay calm even in bad situations.	1	2	3	4
Usually, I have confidence in my ability to fulfill my task.	1	2	3	4

D4. Adolescents would have some romantic feelings for someone of opposite sex when they are growing up. Have you ever had such feelings for any of your schoolmates or friends of the opposite sex?
1. Yes, I have. 2. No, I haven't.

D5. Have you ever been in a relationship?
1. Yes, I have or I'm in a relationship now. 2. No, I have never been in a relationship.

D6. Have you ever done the following things with anyone of the opposite sex? (Please mark all that apply)
1. Holding hands 2. Kissing 3. Other more intimate physical contacts 4. None of the above

D7. What's the attitude of most of your peers toward having relationships among junior high students?
1. Antipathy 2.Indifference 3. Admire
4. Jealousy 5. Others (Please specify__________)

D8. What's the attitude of your teachers and elder members of your family toward having a romance among junior high students?
1. Opposed 2. Not opposed

D9. How many best friends do you have?
[__|__] friends

D10.Please write down names of 5 of your best friends truthfully in the first column of the table below, and then fill in the blanks with number that best describes their conditions. (Please write down as many best friends as you have if there are less than 5.)

Names of 5 of your best friends	Gender 1. Male 2. Female	Location of Hukou 1. In the local county/district 2. Not in the local county/district	Does he/she attend the same school with you? 1. Yes. 2. No.	Is he/she in the same class with you? 1. Yes. 2. No.
1____________	[____]	[____]	[____]	[____]
2____________	[____]	[____]	[____]	[____]
3____________	[____]	[____]	[____]	[____]
4____________	[____]	[____]	[____]	[____]
5____________	[____]	[____]	[____]	[____]

D11. How many of your best friends mentioned above fit in the following descriptions?

	None of them	One or two of them	Most of them
Doing well in academic performance	1	2	3
Studying hard	1	2	3
Expecting to go to college	1	2	3
Skipping classes	1	2	3
Criticized or punished for violating school rules	1	2	3
Always fighting with others	1	2	3
Smoking or drinking alcohol	1	2	3
Always going to net bars or video arcade	1	2	3
Having had or is having a romance	1	2	3
Dropped out of school	1	2	3

D12. Who will be the first one for you to turn to when you want to chat with someone?

1. Schoolmates or good friends 2. Parents 3. A relative of yours
4. Teachers 5. No one

D13. Who will be the first one for you to turn to when you are in trouble?

1. Schoolmates or good friends 2. Parents 3. A relative of yours
4. Teachers 5. No one

D14. Who will be the first one for you to turn to when you need help?

1. Schoolmates or good friends 2. Parents 3. A relative of yours
4. Teachers 5. No one

D15. Do you think your classmates from the local county/district will make friends with those classmates who come from other *URBAN* counties/districts? (Please give your answers based on your assumption if you don't have any classmate from non-local counties/districts.)

1. Yes, they will 2. No, they won’t

D16. Do you think your classmates from the local county/district will make friends with those classmates who come from other *RURAL* counties/districts? (Please give your answers based on your assumption if you don't have any classmate from non-local counties/districts.)

1. Yes, they will 2. No, they won’t

(Please continue to finish Part E)

Part E: Contact Information

We hope to continue the tracking of your growth and improvement in the next year by follow-up surveys. Please leave the contact information of yours and that of your parents, so that we could keep in touch with you (*WE PROMISE THAT WE WILL HOLD ALL THE INFORMATION YOU PROVIDE IN STRICT CONFIDENTCE*):

E1. Your contact information:

Your QQ number: [___|___|___|___|___|___|___|___|___|___]

Your mobile phone number: [___|___|___|___|___|___|___|___|___|___|___]

Your wechat number: ____________________

E2. Your family's telephone number:

Area code: [___|___|___|___] Telephone number: [___|___|___|___|___|___|___|___]

E3. Your father's contact information:

Name: ___________ Mobile phone number: [___|___|___|___|___|___|___|___|___|___|___]

E4. Your mother's contact information:

Name: ___________ Mobile phone number: [___|___|___|___|___|___|___|___|___|___|___]

E5. The contact information of your 3 best friends:

Name	Mobile phone number:	QQ number
1___________	[__|__|__|__|__|__|__|__|__|__|__]	[__|__|__|__|__|__|__|__|__|__]
2___________	[__|__|__|__|__|__|__|__|__|__|__]	[__|__|__|__|__|__|__|__|__|__]
3___________	[__|__|__|__|__|__|__|__|__|__|__]	[__|__|__|__|__|__|__|__|__|__]

This is the end of the questionnaire. Thank you for your engagement and cooperation! We wish you a happy life and a successful future!

China Education Panel Survey
Academic Year 2014-2015
Parent Questionnaire for Grade 8

Class： __________

Student Name : __________

Name of Homeroom Teacher： __________

Province (Autonomous Region/Municipality): _______________

County (District): ___________ City: _______________

Name of School: _______________________________________

\- -

Parents answering this questionnaire do not need to fill in the blanks in the box below.

Code of Questionnaire: [____|____|____|____|____|____]

Code of County/District: [____|____|____|____|____|____]

Hi Dear Parents,

China Education Panel Survey (CEPS) is the first large-scale, nationwide, and longitudinal social survey of junior high students in China. It is jointly conducted by the National Survey Research Center (NSRC) at Renmin University of China and academic institutions throughout the country. From 2013 to 2014, we surveyed nearly 20,000 students as well as their parents, homeroom teachers, subject teachers and school administrators. Through scientific sampling, your child and you have been chosen as subjects of this survey. This year, we hope we can keep following the growth and progress of your child through this follow-up survey. Therefore, we are asking you here to complete this questionnaire and we are very grateful to your understanding, support and help!

There are neither right nor wrong answers to these questions and your answers will not be used to evaluate your child and you. Therefore, please answer all the questions truthfully based on your own ideas and experiences. We pledge here that under the *Statistics Law of the People's Republic of China*, we will hold all the information you are going to provide in strict confidence and it will never be given away to any particular individual or institution. Thank you for your cooperation!

National Survey Research Center (NSRC), Renmin University of China

March, 2015

[Answering Instruction]

When answering a multiple-choice question, please circle the number of your choice; when answering a blank-filling question, please write down words or numbers on the lines in the square brackets.

Part A: Family Education

A1. What is your relationship to this child?

1. Biological father
2. Biological mother
3. Stepfather
4. Stepmother
5. Grandfather on mother's/father's side
6. Grandmother on mother's/father's side
7. Other relative male
8. Other relative female
9. Other non-relative male
10. Other non-relative female

A2. Which of the following people live in the same household with this child *AT PRESENT*? (Please mark all that apply.)

1. Biological father of this child
2. Biological mother of this child
3. Stepfather
4. Stepmother
5. Full or half sibling(s) of this child
6. Grandparent(s) of this child on mother's/father's side
7. Other relative(s) of this child
8. Other non-relative(s)

A3. What language does this child usually speak *AT HOME*?

1. Mandarin Chinese
2. Sometimes dialect of his/her hometown, sometimes mandarin Chinese
3. Dialect of his/her hometown
4. Other

A4. How much pocket money does this child receive *ON AVERAGE EVERY WEEK*?

[___|___|___] Yuan (Please fill in 000 if there is no cost.)

A5. How is this child's New Year money (money given to children as a lunar New Year gift) usually dealt?

1. Kept by parents
2. Kept by this child, and disposed as parents suggested
3. Kept and disposed by the child
4. This child never received New Year money

A6. How often do you usually read books, newspapers, or magazines in your spare time?

1. Never
2. Sometimes
3. Often

A7. How often do you usually surf on the Internet? (Including computer and mobile phone)

1. Never 2. Sometimes 3. Often

A8. How much time do you usually spend on watching TV *EVERYDAY* in your spare time?

1. 0 hour 2. Less than 1 hour 3. 1-2 hours 4. More than 2 hours

A9. Do any family members *WHO LIVE WITH YOUR CHILD* almost drink alcohol every week?

1. Yes, [__] family member(s) do/does. 2. No, they don't.

A10. Do any family members *WHO LIVE WITH YOUR CHILD* smoke?

1. Yes, [__] of my family members do/does. 2. No, none of them does

A11. How often did parents have dinner with this child *IN THE PAST YEAR*?

1. Never 2. Once a year 3. Once every half year
4. Once a month 5. Once a week 6. More than once a week

A12. How often did parents visit museums, zoos, science museums, etc. with this child *IN THE PAST YEAR*?

1. Never 2. Once a year 3. Once every half year
4. Once a month 5. Once a week 6. More than once a week

A13. How often did parents go out to watch movies, shows, sports games, etc. with this child *IN THE PAST YEAR*?

1. Never 2. Once a year 3. Once every half year
4. Once a month 5. Once a week 6. More than once a week

A14. Which family member is taking care of this child's *DAILY LIFE*? (Please mark all that apply.)

0. No one.
1. Father, who spends [___|___] hours on average in this aspect every day in total.
2. Mother, who spends [___|___] hours on average in this aspect every day in total.
3. Sibling(s), who spend(s) [___|___] hours on average in this aspect every day in total.
4. Grandparent(s) from mother's/father's side, who spend(s) [___|___] hours on average in this aspect every day in total.
5. Other (Please specify______), who spend(s) [___|___] hours on average in this aspect every day in total.

A15. Which family member is taking charge of this child's *ASSIGNMENTS AND STUDY*? (Please mark all that apply.)

0. No one.
1. Father, who spend(s) [___|___] hours on average in this aspect every day in total.

2. Mother, who spends [___|___] hours on average in this aspect every day in total.

3. Sibling(s), who spend(s) [___|___] hours on average in this aspect every day in total.

4. Grandparent(s) from mother's/father's side, who spend(s) [___|___] hours on average in this aspect every day in total.

5. Other (Please specify______), who spend(s) [___|___] hours on average in this aspect every day in total.

A16. Which family member is accompanying this child to *PLAY*? (Please mark all that apply.)

0. No one.

1. Father, who spends [___|___] hours on average in this aspect every day in total.

2. Mother, who spends [___|___] hours on average in this aspect every day in total.

3. Sibling(s), who spend(s) [___|___] hours on average in this aspect every day in total.

4. Grandparent(s) from mother's/father's side, who spend(s) [___|___] hours on average in this aspect every day in total.

5. Other (Please specify______), who spend(s) [___|___] hours on average in this aspect every day in total.

A17. Do you care and are you strict with this child about the following?

	I don't care.	I do care about it, but am not strict.	I'm very strict about it.
His/her homework and examination	1	2	3
His/her behavior at school	1	2	3
Whom he/she makes friends with	1	2	3
His/her dress style	1	2	3
Time he/she spends on the Internet	1	2	3
Time he/she spends on watching TV	1	2	3

A18. What do you usually do when this child and you have different opinions?

1. Follow him/her mostly
2. Persuade him/her to agree with me
3. Force him/her to agree with me
4. Follow the one who is right after discussion
5. Leave it over without a conclusion
6. Other (Please specify: ____________)

A19. Did the family members help this child with his/her homework *LAST WEEK*?

1. There is no need
2. Need but no one helped
3. Yes, one or two days
4. Yes, three or four days
5. Yes, almost every day

A20. To what extent could you meet the requirement *THIS SEMESTER* if the teachers require the parents to check their child's homework, such as writing "checked", signing names, etc.?

0. The teachers do not have such requirement

1. Completely 2. Mostly 3. Rarely 4. Not at all

A21. Does this child take any cram school or extra-curricular course *THIS SEMESTER*?

1. Yes, he/she does. 2. No, he/she doesn't.

A22. What is the total cost of his/her cram school or extra-curricular courses *THIS SEMESTER*?

[__|__|__|__|__|__] Yuan (Please fill in 00000 if there is no cost.)

A23. How many friends does this child have at school?

0. None **(SKIP TO A26)**

1. About 1-5 friends 2. About 6-10 friends

3. More than 10 friends 4. Not clear

A24. Do you know the friends who often play together with this child?

0. No, I don't. **(SKIP TO A26)**

1. Yes, I know some of them. 2. Yes, I know all of them.

A25. Do you know the parents of the friends who often play together with this child?

0. No, I don't.

1. Yes, I know some of them. 2. Yes, I know all of them.

A26. How often do you discuss the following with this child?

	**Never**	**Sometimes**	**Often**
Things happened at school	1	2	3
The relationship between he/she and his/her friends	1	2	3
The relationship between he/she and his/her teachers	1	2	3
his/her worries and troubles	1	2	3

A27. How often did this child do the following things IN *THE PAST YEAR*?

	**Never**	**Seldom**	**Sometimes**	**Often**	**Always**
Helping elders	1	2	3	4	5
Following orders and lining up	1	2	3	4	5
Being nice and honest	1	2	3	4	5

A28. How often did this child do the following *IN THE PAST YEAR*?

	**Never**	**Seldom**	**Sometimes**	**Often**	**Always**

Cursing or saying swearwords	1	2	3	4	5
Quarreling with others	1	2	3	4	5
Having a fight with others	1	2	3	4	5
Bullying the weak	1	2	3	4	5
Having a violent temper	1	2	3	4	5
Unable to concentrate on one thing	1	2	3	4	5
Skipping classes, being absent, or truanting	1	2	3	4	5
Copying homework from others, cheating in exams	1	2	3	4	5
Smoking, or drinking alcohol	1	2	3	4	5
Going to net bars or video arcade	1	2	3	4	5

A29. What is the highest level of education do you expect this child to receive?

1. Drop out now
2. Graduate from junior high school
3. Go to technical secondary school or technical school
4. Go to vocational high school
5. Go to senior high school
6. Graduate from junior college
7. Get a bachelor degree
8. Get a Master degree
9. Get a Doctor degree

A30. What kind of job do parents MOST expect this child to do in the future?

1. Government official, staff of public institutions, civil servant
2. Manager or administrator of enterprises/corporations
3. Scientist/engineer/programmer/pilot/spaceman
4. Teacher/doctor/lawyer/accountant/translator
5. Professional designer (such as costume, gardening, or advertisement designer)
6. Artistic performer (including writer/drawer/host/director/screenwriter)
7. Professional athlete
8. Technical worker (including driver/cook/maintenance staff)
9. Soldier/policeman
10. Medium service staff (including stewardess/nurse/barber/cosmetologist), or ordinary office staff
11. Self-employed (such as opening a store)
12. Other (Please specify: __________)
13. I don’t care

A31. Where do you most expect this child to live and work in the future?

1. In rural area
2. In towns/counties
3. In small or medium cities
4. In capital cities
5. In Beijing/Shanghai/Guangzhou
6. Abroad
7. I don’t care

A32. Are you confident in the future of this child?

1. Not confident at all
2. Not so confident
3. Somewhat confident
4. Very confident

(Please continue to finish Part B.)

Part B: The Relationship between Parents and School

B1. Do this child's parents attend the parent meeting *THIS SEMESTER*?

1. The school held one, and the parents did attend.
2. The school held one, but the parents didn't attend.
3. Not held yet, but the parents are going to attend once held
4. Not held yet, and the parents are not going to attend if held

B2. How many times have this child's parents contacted the teacher at school *THIS SEMESTER*?

1. Never   2. Once   3. Two to four times   4. Five times or more

B3. When contacting the child's teacher *THIS SEMESTER*, what did the parents usually talk about with the teacher? (Please mark all that apply.)

0. The parents have never contacted the child's teacher this semester.

1. Schoolwork   2. The child's morals   3. Mental health
4. Physical health   5. Friends of the child   6. Other

B4. How many times has this child's teacher contacted the parents *THIS SEMESTER*?

1. Never   2. Once   3. Two to four times   4. Five times or more

B5. When the teacher contacted you *THIS SEMESTER*, what did he/she usually talk about with you? (Please mark all that apply.)

0. The teacher has never contacted me this semester.

1. Schoolwork   2. The child's morals   3. Mental health
4. Physical health   5. Friends of the child   6. Other

B6. Are you afraid of communicating with the school teacher?

1. Quite afraid   2. A little bit afraid   3. Not afraid at all

B7. In your opinion, is the teacher responsible for this child?

1. Not responsible at all   2. Not so responsible   3. Moderate
4. Somewhat responsible   5. Very responsible

B8. In your opinion, is the teacher patient to your child?

1. Not patient at all   2. Not so patient   3. Moderate
4. Somewhat patient   5. Very patient

B9. In your opinion, which kind of the following students benefits most from the teaching method of the teacher?

1. Students with academic record below average
2. Students with average academic record
3. Students with academic record above average
4. All kinds of students
5. It's hard to say.

B10. Does this child like his/her homeroom teacher?

1. Strongly dislike him/her
2. Somewhat dislike him/her
3. Somewhat like him/her
4. Like him/her very much

B11. Does this child like other teachers at school?

1. Strongly dislike them
2. Somewhat dislike them
3. Somewhat like them
4. Like him/her very much

B12. Does this child like his/her schoolmates?

1. Strongly dislike them
2. Somewhat dislike them
3. Somewhat like them
4. Like him/her very much

B13. Do you agree that it is totally the teachers' responsibility to educate children?

1. Strongly disagree
2. Somewhat disagree
3. It's hard to say
4. Somewhat agree
5. Strongly agree

B14. Which of the following factors do you think have effects on students' grade? (Please mark all that apply.)

1. Parents' discipline
2. Teachers' teaching
3. Class
4. Friends
5. Attendance of cram school
6. Extent of hardworking
7. Talent and capability

If this child has schoolmates from non-local county/district, please answer question B15-B20 according to the real condition; if this child doesn't have schoolmates from non-local county/district, please answer these questions according to your assumption:

B15. What kind of effect do you think will the increase of students from non-local county/district have on the educational quality of the school?

1. Harmful
2. Beneficial
3. No effect

B16. What kind of effect do you think will the increase of students from non-local county/district have on the atmosphere of the school?

1. Harmful
2. Beneficial
3. No effect

B17. Which kind of students do you think will the school teachers like better?

1. Students from local county/district
2. Students from non-local county/district
3. There is no difference

B18. When students from non-local county/district have conflicts with students from local county/district, whom do you think will the school teachers be partial to?

1. Students from local county/district
2. Students from non-local county/district
3. The teacher won't be partial to anyone

B19. Do you think the school teachers are prejudiced against students from non-local county/district?

1. Not prejudiced at all
2. A little bit prejudiced
3. Somewhat prejudiced
4. Very prejudiced

B20. Do you think the school teachers are prejudiced against the *PARTENTS* of students from non-local county/district?

1. Not prejudiced at all
2. A little bit prejudiced
3. Somewhat prejudiced
4. Very prejudiced

(Please continue to finish Part C.)

Part C: School Education

C1. What is the total fee paid to this school *LAST SEMESTER*?

	**Fees**
The total fee paid to this school:	[__|__|__|__|__] Yuan<br>(Please fill in 00000 if it is free.)
Including: Tuition fees	[__|__|__|__|__] Yuan<br>(Please fill in 00000 if it is free.)
Book fees	[__|__|__|__|__] Yuan<br>(Please fill in 00000 if it is free.)
Supplementary education material fees	[__|__|__|__|__] Yuan<br>(Please fill in 00000 if it is free.)
Uniform fees	[__|__|__|__|__] Yuan<br>(Please fill in 00000 if it is free.)
Activity fees	[__|__|__|__|__] Yuan<br>(Please fill in 00000 if it is free)
Insurance fees	[__|__|__|__|__] Yuan<br>(Please fill in 00000 if it is free.)
Meal expenses	[__|__|__|__|__] Yuan<br>(Please fill in 00000 if it is free.)
Dormitory fees	[__|__|__|__|__] Yuan<br>(Please fill in 00000 if it is free, or fill in 99999 if this child does not live in the dorm.)
Other fees (Please specify: ______)	[__|__|__|__|__] Yuan<br>(Please fill in 00000 if it is free.)

C2. Did your child receive the governmental subsidies in education *LAST SEMESTER*? (Please mark all that apply.)

1. Yes, his/her book fees were exempted
2. Yes, the school provided free breakfast/lunch
3. Yes, he/she received the grant aid for students ([__|__|__|__] Yuan/semester)
4. Other (Please specify: ___________________)
5. Not receiving any governmental subsidies

C3. Do you think the size of this child's class is too small or too big?

1. Too small	2. Somewhat small	3. Moderate
4. Somewhat big	5. Too big

C4. What do you think of the general attitude of this child towards schoolwork?

1. Not serious at all 2. Somewhat not serious 3. Moderate
4. Somewhat serious 5. Very Serious

C5. How does this child's academic record rank in his/her class at present?

1. Near the bottom 2. Below the average 3. About the average
4. Above the average 5. Around the top

C6. What is your requirement on this child's academic record?

1. Being one of the top five of his/her class 2. Above the average
3. About the average 4. No special requirement

If you are the parents from non-local county/district, please continue answering question C7-C9 in the following box. If you are the parents from local county/district, please skip to Part D.

C7. According to the local policy, is this child allowed to apply for a senior high school in this city (prefecture-level city)?

1. He/she is allowed to apply for a key senior high school.
2. He/she is allowed to apply for an ordinary senior high school, but not a key one.
3. He/she is not allowed to apply for either an ordinary senior high school or a key one. **(SKIP TO C9)**
4. Not clear **(SKIP TO C9)**

C8. Are there any special requirements that this child needs to meet in order to apply for an ordinary senior high school in this city (prefecture-level city)? (Please mark all that apply.)

0. There are no special requirements
1. Studying in the local (prefecture-level city) junior high school for three years
2. Local Hukou of this city (prefecture-level city) or enough "score" to obtain it
3. Local Hukou of this province
4. The property ownership certificate or lease contract of parents' house/apartment
5. (Temporary) residential permission
6. Social security for at least one year
7. Family planning certificate
8. Business license or employment certificate
9. Other (Please specify :______________)

C9. What is your plan for this child after he/she graduates from junior high school?

1. To study in a senior high school in this city
2. To study in a senior high school in his/her hometown
3. To study in a senior high school in another place
4. To study in a vocational high school, technical secondary school or technical school in this city
5. To get a job

6 Other (Please specify: ______________)

(Please continue to finish Part D.)

Part D: Basic Information of This Child

D1. The location of his/her Hukou *AT PRESENT* is:

1. In the local county/district
2. Not in the local county/district: County (District): [_________], City: [_________], Province (Autonomous Region/Municipality): [_________]

D2. What is the type of his/her Hukou *AT PRESENT*? (Agricultural Hukou refers to rural Hukou, a record that identifies a person as a rural resident. Non-agricultural Hukou refers to urban Hukou. Residential Hukou is a general record of residence assigned to all residents of a certain region, regardless of rural or urban background)

1. Agricultural Hukou **(SKIP TO D4)**
2. Non-agricultural Hukou
3. Residential Hukou
4. Other **(SKIP TO D4)**

D3. In which year did this child obtain the non-agricultural Hukou?

In the year of [__|__|__|__]

(Please fill in 9997 if this child obtained the non-agricultural Hukou since the beginning.)

D4. *ACCORDING TO THE BIRTH CERTIFICATE*, what is the *BIRTHWEIGHT* of this child?

[__]. [__] kilograms

(Please notice that the unit of the weight is kilogram, and 1 decimal digit is reserved. Please fill in 0.0 if you are not clear about the exact birthweight of this child.)

D5. *ACCORDING TO THE BIRTH CERTIFICATE*, how old are the parents when they *HAVE THIS CHILD*?

Father: [___|___] years old

Mother: [___|___] years old

D6. *UP TILL NOW*, has the child ever had one of the following serious illnesses?

Kidney disease:	1. No, he/she hasn't<br>2. Yes, he/she had it when he/she was [__|__] years old and recovered when he/she was [__|__] years old (Fill in 99 if he/she hasn't recovered yet)
Lung disease:	1. No, he/she hasn't<br>2. Yes, he/she had it when he/she was [__|__] years old and recovered when he/she was [__|__] years old (Fill in 99 if he/she hasn't recovered yet)

Heart disease:	1. No, he/she hasn’t<br>2. Yes, he/she had it when he/she was [__|__] years old and recovered when he/she was [__|__] years old (Fill in 99 if he/she hasn’t recovered yet)
Brain disease:	1. No, he/she hasn’t<br>2. Yes, he/she had it when he/she was [__|__] years old and recovered when he/she was [__|__] years old (Fill in 99 if he/she hasn’t recovered yet)
Upper limb fracture:	1. No, he/she hasn’t<br>2. Yes, he/she had it when he/she was [__|__] years old and recovered when he/she was [__|__] years old (Fill in 99 if he/she hasn’t recovered yet)
Lower limb fracture:	1. No, he/she hasn’t<br>2. Yes, he/she had it when he/she was [__|__] years old and recovered when he/she was [__|__] years old (Fill in 99 if he/she hasn’t recovered yet)

D7. What is the current height and weight of this child?

Height: [___|___|___] centimeters

Weight: [___|___|___] kilograms

(Please notice that the unit of the height and weight is centimeter and kilogram, respectively.)

D8. Which one of the following best describes the general health condition of this child *AT PRESENT*?

1. Very poor   2. Not very good   3. Moderate

4. Good   5. Very good

D9. Is this child short-sighted *AT PRESENT*?

1. Yes. he/she is.

The strength of his/her left eyeglass is [__|__|__] degrees.

The strength of his/her right eyeglass is [__|__|__] degrees.

2. Yes, he/she is But I am not clear about the strength of his/her glasses/lens.

3. No. he/she isn’t. **(SKIP TO D11)**

D10. Is this child born with myopia?

1. Yes, he/she is.   2. No, he/she isn’t.   3. Not clear.

D11. Does this child have the following disorders *AT PRESENT*? (Please mark all that apply.)

0. None

1. Vision disorders (not including short-sighted)

2. Hearing disorders

3. Extremity Disabled

4. Language Disorders

5. Autism or other mental disorders
6. Attention deficit hyperactivity disorder (ADHD)
7. Epilepsy
8. Other (Please specify: _____________)

D12. Which of the following medical insurance does this child have *AT PRESENT*? (Please mark all that apply.)

1. Reimburse for child's medical expenses by parents' work units
   (The proportion of the reimbursement of the total medical expenses is [__|__|__]%.)
2. The basic medical insurance for urban residents
3. The new rural cooperative medical insurance
4. The medical insurance for children
5. Accidental injury insurance inside/outside school
6. Commercial medical insurance
7. Other medical insurance (Please specify: _____________________)
8. This child doesn't have any medical insurance at present.

D13. Does this child fit in the following descriptions *AT PRESENT*?

	**Not fit at all**	**Somewhat not fit**	**Somewhat fit**	**Exactly fit**
Able to express himself/ herself clearly	1	2	3	4
Able to give quick responses	1	2	3	4
A faster learner	1	2	3	4
Curious about new stuff	1	2	3	4

(Please continue to finish Part E.)

Part E: Parents, Family and Community

E1. In which year was this child's father born?

In the year of [___|___|___|___]

E2. The ethnic nationality of this child's father is:

1. The Han nationality
2. The Mongol nationality
3. The Manchu nationality
4. The Hui nationality
5. The Tibetan nationality
6. The Zhuang nationality
7. The Uygur nationality
8. Other (Please specify: __________)

E3. Where does this child's father live *AT PRESENT*?

1. In the local county/district
2. Not in the local county/district

E4. What is the type of this child's father's Hukou *AT PRESENT*?

1. Agricultural Hukou **(SKIP TO E6)**
2. Non-agricultural Hukou
3. Residential Hukou
4. Other **(SKIP TO E6)**

E5. In which year did this child's father obtain the non-agricultural Hukou?

In the year of [__|__|__|__]

(Please fill in 9997 if this child's father obtained the non-agricultural Hukou since the beginning.)

E6. The location of this child's father's Hukou is:

1. In the local county/district
2. Not in the local county/district: County/district: [_________], City: [_________], Province (Autonomous Region/Municipality): [_________]

E7. What is the political affiliation of this child's father?

1. Member of the Communist Party
2. Member of a Democratic Party
3. The masses

E8. What is the highest education level of this child's father?

1. None
2. Finished elementary school
3. Junior high school degree
4. Technical secondary school or technical school degree
5. Vocational high school degree
6. Senior high school degree
7. Junior college degree
8. Bachelor degree
9. Master degree or higher

E9. What is the *CURRENT* occupation of this child's father?

1. Government official/cadre
2. Cadre/Official/Administrator of public institutions, enterprises/corporations
3. Scientist, engineer, university professor or other professionals
4. Doctor, lawyer, high school/primary school teacher
5. Accountant, nurse, computer programmer or other technical staff
6. Ordinary staff or worker (for example: secretary, bank clerk, or librarian)
7. Ordinary staff or worker in business or service (for example: salesperson, agent, cook, barber, or cosmetologist)
8. Technical worker (for example: driver, electrician, plumber, or mechanist)
9. Ordinary worker (for example: porter, or production line worker)
10. Farmer, herdsman, fisherman
11. Elementary worker (for example: cleaner, guard, housekeeper, or sanitation worker)
12. Self-employed worker
13. Unemployed or laid-off worker
14. Other (Please specify_________)

E10. In which year was this child's mother born?

In the year of [___|___|___|___]

E11. The ethnic nationality of this child's mother is:

1. The Han nationality 2. The Mongol nationality
3. The Manchu nationality 4. The Hui nationality
5. The Tibetan nationality 6. The Zhuang nationality
7. The Uygur nationality 8. Other (Please specify: __________)

E12. Where does this child's mother live *AT PRESENT*?

1. In the local county/district 2. Not in the local county/district

E13. What is the type of this child's mother's Hukou *AT PRESENT*?

1. Agricultural Hukou **(SKIP TO E15)** 2. Non-agricultural Hukou
3. Residential Hukou 4. Other **(SKIP TO E15)**

E14. In which year did this child's mother obtain the non-agricultural Hukou?

In the year of [__|__|__|__]

(Please fill in 9997 if this child's mother obtained the non-agricultural Hukou since the beginning.)

E15. The location of this child's mother's Hukou is:

1. In the local county/district
2. Not in the local county/district: County/district: [_________], City: [_________], Province (Autonomous Region/Municipality): [_________]

E16. What is the political affiliation of this child's mother?

1. Member of the Communist Party
2. Member of a Democratic Party
3. The masses

E17. What is the highest education level of this child's mother?

1. None
2. Finished elementary school
3. Junior high school degree
4. Technical secondary school or technical school degree
5. Vocational high school degree
6. Senior high school degree
7. Junior college degree
8. Bachelor degree
9. Master degree or higher

E18. What is the *CURRENT* occupation of this child's mother?

1. Government official/cadre
2. Cadre/Official/Administrator of staff of public institutions, enterprises/corporations
3. Scientist, engineer, university professor or other professionals
4. Doctor, lawyer, high school/primary school teacher
5. Accountant, nurse, computer programmer or other technical staff
6. Ordinary staff or worker (for example: secretary, bank clerk, or librarian)
7. Ordinary staff or worker in business or service (for example: salesperson, agent, cook, barber, or cosmetologist)
8. Technical worker (for example: driver, electrician, plumber, or mechanist)
9. Ordinary worker (for example: porter, or production line worker)
10. Farmer, herdsman, fisherman
11. Elementary worker (for example: cleaner, guard, housekeeper, or sanitation worker)
12. Self-employed worker
13. Unemployed or laid-off worker
14. Other (Please specify_________)

Please continue to answer E19 if you are not this child's parent. And please skip to E23 if you are this child's parent.

E19. In which year were you born?

In the year of [___|___|___|___]

E20. What is the type of your Hukou at present?

1. Agricultural Hukou   2. Non-agricultural Hukou
3. Residential Hukou   4. Other

E21. What is the highest education level you have completed?

1. None   2. Finished elementary school
3. Junior high school degree   4. Technical secondary school or technical school degree
5. Vocational high school degree   6. Senior high school degree
7. Junior college degree   8. Bachelor degree
9. Master degree or higher

E22. What is your *CURRENT* occupation?

1. Government official/cadre
2. Cadre/Official/Administrator of staff of public institutions, enterprises/corporations
3. Scientist, engineer, university professor or other professionals
4. Doctor, lawyer, high school/primary school teacher
5. Accountant, nurse, computer programmer or other technical staff
6. Ordinary staff or worker (for example: secretary, bank clerk, or librarian)
7. Ordinary staff or worker in business or service (for example: salesperson, agent, cook, barber, or cosmetologist)
8. Technical worker (for example: driver, electrician, plumber, or mechanist)
9. Ordinary worker (for example: porter, or production line worker)
10. Farmer, herdsman, fisherman
11. Elementary worker (for example: cleaner, guard, housekeeper, or sanitation worker)
12. Self-employed worker
13. Unemployed or laid-off worker
14. Other (Please specify_________)

E23. Which one of the following best describes the financial conditions of your family *AT PRESENT*?

1. Very poor   2. Somewhat poor   3. Moderate
4. Somewhat rich   5. Very rich

E24. Do your family receive subsistence allowance *AT PRESENT*?

1. Yes, we do.   2. No, we don’t.

E25. Which one of following best describes the income level of your family compared with that of others in your community?

1. Very Low 2. Somewhat low 3. Average
4. Somewhat high 5. Very high

E26. Is there anyone in your family that needs long-term care because of illness or mobility problems?
1. Yes, there is. 2. No, there isn't.

E27. *REGARDLESS OF BUYING OR RENTING A HOUSE*, what type of house/apartment do you live in *AT PRESENT*?
1. Temporary work shed 2. Basement
3. Rural bungalow 4. Rural building with more than one story
5. Urban bungalow 6. Ordinary urban apartment
7. Urban housing estate 8. Other (Please specify: ____________)

E28. Is the building/house/apartment you are living in also used for production/business?
1. Yes, it is. 2. No, it isn't.

E29. How many rooms are there in the building/house/apartment you are living in? (Including living rooms and bedrooms, but not including kitchens or bathrooms)
[__|__] room(s)

E30. Is there tap water (running water) in your building/house/apartment?
1. Yes, there is. 2. No, there isn't.

E31. Is there a private toilet in your building/house/apartment?
1. Yes, there is. 2. No, there isn't.

E32. Which kind of the following toilets is used by your family at present?
1. Flush Toilet 2. Squat Toilet 3. Cement Cesspit 4. Soil Cesspit

E33. Which kind of area are you living in?
1. Central area of the city/county
2. Outskirts of the city/county
3. The "rural-urban continuum" area of the city/county
4. Towns outside the city/county
5. Rural area
6. Other (Please specify: ____________)

E34. Which kind of community are you living in?

1. Old-style city community without reconstruction
2. Single or compound community affiliated to a work unit (Danwei)
3. Ensuring housing supplied by social secure/subsidy system
4. Ordinary commercial residential building
5. Elite housing
6. Villages inside the city ("Cheng Zhong Cun")
7. Rural Area
8. Other (Please specify: ____________)

E35. Which kind of neighborhood are you living with?

1. Mostly local people   2. Mostly migrant people

E36. What is the social status of most of your neighbors?

1. Lower class   2. Middle class   3. Upper class

E37.Are there any of the following facilities provided in the community you live in? (Please mark all that apply.)

1. Public transportation   2. Kindergarten   3. Hospital   4. Park

E38. What do you think of the safety of the community you live in?

1. Very bad   2. Somewhat bad   3. Somewhat good   4. Very good

E39. What do you think of the cleanness of the community you live in?

1. Very bad   2. Somewhat bad   3. Somewhat good   4. Very good

E40. What do you think of the pollution condition of the community you live in?

1. Very bad   2. Somewhat bad   3. Somewhat good   4. Very good

Part F: Contact Information

We hope to continue the tracking of the growth and improvement of this child in the next year by follow-up surveys. Please leave the contact information of the parents, so that we could keep in touch with this child and his/her parents:

F1. Telephone number of this child's family:

Area code: [___|___|___|___] Telephone number: [___|___|___|___|___|___|___|___]

F2. The contact information of this child's father:

Name: ____________ Mobile phone number: [___|___|___|___|___|___|___|___|___|___|___]

F3. The contact information of this child's mother:

Name: ____________ Mobile phone number: [___|___|___|___|___|___|___|___|___|___|___]

This is the end of the questionnaire. Thank you very much for your support!

We wish you a healthy and happy life!

Class: ________

Name of Subject Teacher: ____________

Name of School: ____________________

Location of School: County/District: ___________ City: ___________

Province (Autonomous Region/Municipality): ____________

China Education Panel Survey

Academic Year 2014-2015

Subject Teacher Questionnaire

Teachers answering this questionnaire do not need to fill in the blanks in the box below.

1. Code of Questionnaire: [____|____|____|____|____|____]

2. Code of County/District: [____|____|____|____|____|____]

Distinguished teachers,

Hello!

China Education Panel Survey (CEPS) is the first large-scale, nationwide, and longitudinal social survey of junior high students in China. It is jointly conducted by the National Survey Research Center (NSRC) at Renmin University of China and academic institutions throughout the country. During the academic year of 2013-2014, we surveyed nearly 20,000 students as well as their parents, homeroom teachers, subject teachers and school administrators. Through scientific sampling, your class has been chosen as the sample class. This year, we hope this follow-up survey would help us understand the growth and progress of your students. We would appreciate it very much if you could help us finish this questionnaire. Thank you for your understanding and support.

There are neither right nor wrong answers to these questions and your answers will not be evaluated as part of your work performance or that of your school. Therefore, please write down your honest ideas and the reality of the sample class. We pledge here that under *the Statistics Law of the People's Republic of China*, we will hold all the information you are going to provide in strict confidence and it will never be given away to any individual or institution. Thank you for your cooperation.

National Survey Research Center (NSRC), Renmin University of China

March, 2015

[Answering Instruction]

When answering a multiple-choice question, please circle the number of your choice; when answering a blank-filling question, please write down words or numbers on the lines in the square brackets.

Part A: Teaching

A1. What subject do you teach for this class?
1. Math 2. Chinese 3. English

A2. When did you begin to teach this class?
In the year of [__|__|__|__]

A3. How was the academic performance of your class compared with other classes of the same grade *WHEN YOU BEGAN TEACHING THIS CLASS*?
1. Near the bottom 2. Below average 3. Average
4. Above average 5. Among the best

A4. *AT PRESENT,* how is the academic performance of your class compared with other classes of the same grade?
1. Near the bottom 2. Below average 3. Average
4. Above average 5. Among the best

A5. *AT PRESENT* is the grade to which the sample class belongs divided according to the total score or the score of a single subject?
1. Yes 2. No

A6. How many *OTHER CLASSES* do you teach besides the sample class?
[__|__] class(s)

A7. How many class hours did you teach *LAST WEEK*?
[__|__] hour(s)

A8. How many hours did you spend on preparing lessons *LAST WEEK*?
[__|__] hour(s) (Please fill in 00 if you don't need to prepare lessons.)

A9. How many hours did you spend on correcting homework and/or test papers *LAST WEEK*?
[__|__] hour(s) (Please fill in 00 if you don't need to correct homework or test papers.)

A10. How long was your total working hours *LAST WEEK*?
[___|___|___] hour(s) (including teaching, preparing lessons, correcting homework and/or test papers, and home visiting.)

A11. Do you often discuss the subject you teach with your colleagues *SINCE THE BEGINNING OF THIS SEMESTER*?
1. Yes, I do. 2. No, I don't.

A12. What's the most discussed teaching-related topic between you and your colleagues? (Please mark all that apply)
1. Content of textbooks 2. Teaching methods
3. Exam paper design 4. Students' tutoring

A13. How often do you apply each of the following teaching methods to the sample class?

	Never	Seldom	Sometimes	Often	Always
Lectures	1	2	3	4	5
Group discussion	1	2	3	4	5
Interaction between the teacher and students	1	2	3	4	5

A14. How often do you adopt each of the following facilities in your teaching?

	Never	Seldom	Sometimes	Often	Always
Multimedia projector	1	2	3	4	5
Internet	1	2	3	4	5
Wall maps, models, posters	1	2	3	4	5
Personal teaching website, blog or microblog	1	2	3	4	5

A15. What's the proportion of each of the following teaching materials used in your teaching?

	Percentage
Domestic textbooks	[__|__|__]%
Foreign textbooks	[__|__|__]%
Reference materials	[__|__|__]%
Teaching materials designed by yourself	[__|__|__]%
Total	100%

A16. How much pressure do you have concerning the following aspects?

	None	Low	Moderate	High	Very high
Students' poor academic records	1	2	3	4	5
Students' uneven academic records	1	2	3	4	5
School's evaluation of teachers	1	2	3	4	5
School's administrative measures	1	2	3	4	5
Requirements of students' parents	1	2	3	4	5
Students' enrollment rate	1	2	3	4	5
Public opinions about teachers	1	2	3	4	5
Publication of academic papers	1	2	3	4	5

A17. Take the main subject you teach as an example, how great is the influence of each of the following elements on students' academic record?

	Almost no influence	Some influence	Very great influence
Students' talent	1	2	3
Students' attitude to learning	1	2	3
Students' method of learning	1	2	3
Students' family background	1	2	3
Students' circle of friends	1	2	3
Teachers' teaching method	1	2	3
The amount of attention paid to students by the teacher	1	2	3
Teachers' salary	1	2	3
The school's management	1	2	3
The school's teaching facilities	1	2	3

A18. Generally speaking, how many students in this class pay respect to you?

1. Very few
2. Less than half
3. Half of the class
4. More than half
5. Almost everyone

A19. What kind of teaching method do you think best suits the following types of classes?

	Lectures	Group discussion	Interaction between the teacher and students
Classes composed of only local students	1	2	3
Classes in which the majority are local students and only a small number are from non-local counties/districts	1	2	3
Classes in which more than one third are from non-local counties/districts	1	2	3

A20. What's your opinion of the following aspects of each type of classes?

	**Effect of teaching:**<br>1. Very bad<br>2. Bad<br>3. Moderate<br>4. Good<br>5. Very good	**Class discipline:**<br>1. Very bad<br>2. Bad<br>3. Moderate<br>4. Good<br>5. Very good	**Relations among students:**<br>1. Very bad<br>2. Bad<br>3. Moderate<br>4. Good<br>5. Very good
Classes composed of only local students	[___]	[___]	[___]
Classes in which the majority are local students and only a small number are from non-local counties/districts	[___]	[___]	[___]

	**Effect of teaching:**<br>1. Very bad<br>2. Bad<br>3. Moderate<br>4. Good<br>5. Very good	**Class discipline:**<br>1. Very bad<br>2. Bad<br>3. Moderate<br>4. Good<br>5. Very good	**Relations among students:**<br>1. Very bad<br>2. Bad<br>3. Moderate<br>4. Good<br>5. Very good
Classes in which more than one third are from non-local counties/districts	[___]	[___]	[___]

A21. Which type of class would you like to be in charge of if you can make a choice?

1. Classes composed of only local students
2. Classes in which the majority are local students and only a small number are from non-local counties/districts
3. Classes in which more than one third are from non-local counties/districts
4. I don't care

A22. Assuming that a student named Xiaoming in Grade 8 cannot stay focused in class or sit still, and is always playing while studying, overactive and impulsive, what would you do if you met a student like him? (Please mark all that apply)

1. I would be unconcerned and isolate Xiaoming
2. I would criticize Xiaoming in public
3. I would criticize Xiaoming in private
4. I would try to help Xiaoming
5. I would ask Xiaoming's parents to help him together
6. I would tell other students not to isolate Xiaoming
7. I would treat it as a normal phenomenon and do nothing

A23. Assuming that a student named Xiaohong in Grade 8 recently become depressed, passionless, and slow in thinking and acting, what would you do if you met a student like her? (Please mark all that apply)

1. I would be unconcerned and isolate Xiaohong
2. I would criticize Xiaohong in public
3. I would criticize Xiaohong in private
4. I would try to help Xiaohong
5. I would ask Xiaohong's parents to help her together
6. I would tell other students not to isolate Xiaohong
7. I would treat it as a normal phenomenon and do nothing

Part B: Background and Opinions

B1. Your sex is:

1. Male 2.Female

B2. Your year of birth is: [__|__|__|__]

B3. What is your current marital status?

1. Not married 2. Married 3. Widowed
4. Divorced/separated 5. Other

B4. Up till now, what is the highest educational diploma you have attained?

1. Junior high school degree or below
2. Vocational high school/technical secondary school/technical school degree
3. Senior high school degree
4. Junior college degree
5. Bachelor degree (attained through adult higher education)
6. Bachelor degree (attained through regular higher education)
7. Master degree or higher

B5. Are you a graduate from pedagogical universities or pedagogical majors?

1. Yes. 2. No.

B6. Do you have a teacher certification?

1. Yes. 2. No.

B7. Have you ever received psychological health training?

1. Yes, and I have license A. 2. Yes, and I have license B. 3. No.

B8. How many years of experience do you have in teaching?

[__|__] year(s)

B9. Have you ever taught in other schools before you came to this school?

1. No 2. Yes

B10. When did you begin to teach in this school?

[__|__|__|__][__|__] ([YYYY] [MM])

B11. Are you an institutionally registered teacher admitted by the government?

1. Yes, I became a registered teacher in the year [__|__|__|__].
2. No, I'm not.

B12. Do you have a professional title in teaching? If yes, what is it?

0. No, I don't.
1. Level-C Teacher 2. Level-B Teacher 3. Level-A Teacher
4. Senior-Level Teacher 5. Principal Senior-Level Teacher

B13. Are you holding other concurrent administrative posts? If yes, what is it?

0. No, I'm not
1. Head of the grade
2. Head of the teaching & research group of a subject
3. Teaching director or deputy teaching director
4. Deputy principal
5. Principal

B14. Do you receive government subsidies?

1. Yes 2. No

B15. Which of the following grades have you ever taught? (Please mark all that apply.)

1. The seventh grade 2. The eighth grade 3. The ninth grade
4. Elementary school grades 5. Senior high school grades

B16.Which of the following awards have you ever won because of your teaching performance? (Please mark all that apply.)

1. National award 2. Province-level award 3. City-level award
4. County-level award 5. School-level award 6. None

B17. Have you ever won the title of Excellent Teacher over the past three years?

1. Yes, more than once 2. Yes, I got the title once
3. No 4. There is no such title in this school's evaluation system

B18. In general, are you satisfied with your current job as a teacher?

1. Strongly unsatisfied 2. Somewhat unsatisfied 3. Moderate
4. Somewhat satisfied 5. Strongly satisfied

B19. How many close friends do you have among your colleagues in this school?

1. None 2. Just a few 3. Some 4. Many

B20. Have you ever feel tired of being a teacher?

1. Never 2. Seldom 3. Sometimes 4. Often

B21. Are you still willing to choose teacher as your job if you have a second chance to make a choice?

1. No 2. Not sure 3. Definitely yes

B22. Are you still willing to become a junior-high teacher if you have a second chance to make a choice?

1. No 2. Not sure 3. Definitely yes

B23. Are you still willing to teach in this school if you have a second chance to choose an institution to work for?

1. No 2. Not sure 3. Definitely yes

B24. Are you satisfied with each of the following aspects of this school?

	Strongly unsatisfied	Somewhat unsatisfied	Moderate	Somewhat satisfied	Strongly satisfied
Salary	1	2	3	4	5
School management	1	2	3	4	5
Facility	1	2	3	4	5
Students' quality	1	2	3	4	5

B25. What do you think of the school's academic atmosphere?

1. Very bad   2. Bad   3. Moderate   4. Good   5. Very good

B26. What do you think of the school's management of students?

1. Very loose   2.Loose   3. Moderate   4.Strict   5. Very strict

B27. In your opinion, what are the capabilities or qualities of students that the school needs to improve its educating further? (Please mark all that apply.)

1. The capability of analyzing and solving problems
2. Creativity and imagination
3. Self-discipline and perseverance
4. Positive values and morality
5. Interpersonal relationship, communication and cooperation
6. Mental health
7. Concerns for and engagement in public affairs

B28. In what aspects do you think the school should bear more responsibility than students' family? (Please mark all that apply.)

1. The capability of analyzing and solving problems
2. Creativity and imagination
3. Self-discipline and perseverance
4. Positive values and morality
5. Interpersonal relationship, communication and cooperation
6. Mental health
7. Concerns for and engagement in public affairs

B29. What do you think is the most important goal of school education? (Please mark all that apply.)

1. To foster virtue
2. To pass on basic knowledge
3. To foster comprehensive capabilities
4. To increase senior high school/college/university acceptance rate
5. To make sure students can grow up happily and safely

Part C: Contact Information

We hope to continue the tracking of the students' future progress through this panel survey. Please leave your contact information so that we could keep in touch with you and your students:

C1. Your office phone number:

C1a. Area code: [__| __ | __ | __]

C1b. Phone number: [__| __ | __ | __ | __ | __ | __ | __]

C2. Your mobile phone number: [__| __ | __ | __ | __ | __ | __ | __ | __ | __ | __]

C3. Your Email address: ___________________

C4. Your QQ number: [__ | __ | __ | __ | __ | __ | __ | __ | __ | __]

This is the end of the questionnaire. Thank you for your engagement and cooperation! We wish you a good health and successful career!

Class: ________

Name of Homeroom Teacher: ____________

Name of School: ___________________

Location of School: County/District: ___________ City: ___________

Province (Autonomous Region/Municipality): ___________

China Education Panel Survey

Academic Year 2014-2015

Homeroom Teacher Questionnaire

Teachers answering this questionnaire do not need to fill in the blanks in the box below.

1. Code of Questionnaire: [____|____|____|____|____|____]

2. Code of County/District: [____|____|____|____|____|____]

Distinguished teachers,

Hello!

China Education Panel Survey (CEPS) is the first large-scale, nationwide, and longitudinal social survey of junior high students in China. It is jointly conducted by the National Survey Research Center (NSRC) at Renmin University of China and academic institutions throughout the country. During the academic year of 2013-2014, we surveyed nearly 20,000 students as well as their parents, homeroom teachers, subject teachers and school administrators. Through scientific sampling, your class has been chosen as the sample class. This year, we hope this follow-up survey would help us understand the growth and progress of your students. We would appreciate it very much if you could help us finish this questionnaire. Thank you for your understanding and support.

There are neither right nor wrong answers to these questions and your answers will not be evaluated as part of your work performance or that of your school. Therefore, please write down your honest ideas and the reality of the sample class. We pledge here that under *the Statistics Law of the People's Republic of China*, we will hold all the information you are going to provide in strict confidence and it will never be given away to any individual or institution. Thank you for your cooperation.

National Survey Research Center (NSRC), Renmin University of China

March, 2015

[Answering Instruction]

When answering a multiple-choice question, please circle the number of your choice; when answering a blank-filling question, please write down words or numbers on the lines in the square brackets.

Part A: Teaching

A1. What subject(s) do you teach as the homeroom teacher of this class? (Please mark all that apply.)

1. Math 2. Chinese 3. English 4. Physics
5. Chemistry 6. Biology 7. History 8. Geography
9. Politics 10. Other (Please specify: __________)

A2. When did you take the post of homeroom teacher of this class?

In the year of [__|__|__|__]

A3. How was the academic performance of your class compared with other classes of the same grade *WHEN YOU TOOK THE POST* of homeroom teacher of *THIS CLASS*?

1. Near the bottom 2. Below average 3. Average
4. Above average 5. Among the best

A4. *AT PRESENT,* how is the academic performance of your class compared with other classes of the same grade?

1. Near the bottom 2. Below average 3. Average
4. Above average 5. Among the best

A5. *AT PRESENT,* is the grade to which the sample class belongs divided according to the total score or the score of a single subject?

1. Yes 2. No

A6. How many *OTHER CLASSES* do you teach besides the sample class?

[__|__] class(s)

A7. How many class hours did you teach *LAST WEEK*?

[__|__] hour(s)

A8. How many hours did you spend on preparing lessons *LAST WEEK*?

[__|__] hour(s) (Please fill in 00 if you don't need to prepare lessons.)

A9. How many hours did you spend on correcting homework and/or test papers *LAST WEEK*?

[__|__] hour(s) (Please fill in 00 if you don't need to correct homework or test papers.)

A10. How long was your total working hours *LAST WEEK*?

[___|___|___] hour(s) (including teaching, preparing lessons, correcting homework and/or test papers, and home visiting.)

A11. Do you often discuss the subject you teach with your colleagues *SINCE THE BEGINNING OF THIS SEMESTER*?

1. Yes, I do. 2. No, I don't.

A12. What's the most discussed teaching-related topic between you and your colleagues? (Please mark all that apply.)

1. Content of textbooks
2. Teaching methods
3. Exam paper design
4. Students' tutoring

A13. How often do you apply each of the following teaching methods to the sample class?

	Never	Seldom	Sometimes	Often	Always
Lectures	1	2	3	4	5
Group discussion	1	2	3	4	5
Interaction between the teacher and students	1	2	3	4	5

A14. How often do you adopt each of the following facilities in your teaching?

	Never	Seldom	Sometimes	Often	Always
Multimedia projector	1	2	3	4	5
Internet	1	2	3	4	5
Wall maps, models, posters	1	2	3	4	5
Personal teaching website, blog or microblog	1	2	3	4	5

A15. What's the proportion of each of the following teaching materials used in your teaching?

	Percentage
Domestic textbooks	[__|__|__]%
Foreign textbooks	[__|__|__]%
Reference materials	[__|__|__]%
Teaching materials designed by yourself	[__|__|__]%
Total	100%

A16. How much pressure do you have concerning the following aspects?

	None	Low	Moderate	High	Very high
Students' poor academic records	1	2	3	4	5
Students' uneven academic records	1	2	3	4	5
School's evaluation of teachers	1	2	3	4	5
School's administrative measures	1	2	3	4	5

	None	Low	Moderate	High	Very high
Requirements of students' parents	1	2	3	4	5
Students' enrollment rate	1	2	3	4	5
Public opinions about teachers	1	2	3	4	5
Publication of academic papers	1	2	3	4	5

A17. Take the main subject you teach as an example, how great is the influence of each of the following elements on students' academic record?

	Almost no influence	Some influence	Very great influence
Students' talent	1	2	3
Students' attitude to learning	1	2	3
Students' method of learning	1	2	3
Students' family background	1	2	3
Students' circle of friends	1	2	3
Teachers' teaching method	1	2	3
The amount of attention paid to students by the teacher	1	2	3
Teachers' salary	1	2	3
The school's management	1	2	3
The school's teaching facilities	1	2	3

A18. Generally speaking, how many students in this class pay respect to you?

1. Very few 2. Less than half 3. Half of the class
4. More than half 5. Almost everyone

A19. What kind of teaching method do you think best suits the following types of classes?

	Lectures	Group discussion	Interaction between the teacher and students
Classes composed of only local students	1	2	3
Classes in which the majority are local students and only a small number are from non-local counties/districts	1	2	3
Classes in which more than one third are from non-local counties/districts	1	2	3

A20. What's your opinion of the following aspects of each type of classes?

	**Effect of teaching:**<br>1. Very bad<br>2. Bad<br>3. Moderate<br>4. Good<br>5. Very good	**Class discipline:**<br>1. Very bad<br>2. Bad<br>3. Moderate<br>4. Good<br>5. Very good	**Relations among students:**<br>1. Very bad<br>2. Bad<br>3. Moderate<br>4. Good<br>5. Very good
Classes composed of only local students	[___]	[___]	[___]
Classes in which the majority are local students and only a small number are from non-local counties/districts	[___]	[___]	[___]
Classes in which more than one third are from non-local counties/districts	[___]	[___]	[___]

A21. Which type of class would you like to be in charge of if you can make a choice?

1. Classes composed of only local students
2. Classes in which the majority are local students and only a small number are from non-local counties/districts
3. Classes in which more than one third are from non-local counties/districts
4. I don't care

A22. Assuming that a student named Xiaoming in Grade 8 cannot stay focused in class or sit still, and is always playing while studying, overactive and impulsive, what would you do if you met a student like him? (Please mark all that apply)

1. I would be unconcerned and isolate Xiaoming
2. I would criticize Xiaoming in public
3. I would criticize Xiaoming in private
4. I would try to help Xiaoming
5. I would ask Xiaoming's parents to help him together
6. I would tell other students not to isolate Xiaoming
7. I would treat it as a normal phenomenon and do nothing

A23. Assuming that a student named Xiaohong in Grade 8 recently become depressed, passionless, and slow in thinking and acting, what would you do if you met a student like her? (Please mark all that apply)

1. I would be unconcerned and isolate Xiaohong
2. I would criticize Xiaohong in public
3. I would criticize Xiaohong in private
4. I would try to help Xiaohong
5. I would ask Xiaohong's parents to help her together
6. I would tell other students not to isolate Xiaohong
7. I would treat it as a normal phenomenon and do nothing

(Please continue to finish Part B.)

Part B: Class Management

B1. How many parents of your students do you know about? (Here "know about" means that you have met and talked with them, and know their basic situation.)

1. None 2. Just a few 3. About a half 4. More than half 5. All of them

B2. How do you usually contact the parents of your students? (Please mark all that apply)

1. Home visits 2. Phone calls 3.Text messages 4. E-mails
5. QQ 6. Other 7. There's no need to contact parents

B3. How often have you had conflicts with the parents of your students *SINCE THE BEGINNING OF THIS SEMESTER*? (Such as quarrelling with the parents due to issues concerning students' academic record, class management, etc. or being reported to the school administrators by the parents.)

	**The number of parents you have had conflicts with:**<br><br>1. None<br>2. One to four<br>3. Five or above	**The major means you have adopted to settle these conflicts:**<br>1. Your negotiation with the parents<br>2. The school's negotiation with the parents<br>3. Higher educational departments' negotiation with the parents<br>4. Other
Students' academic performance	[__]	[__]
Students' discipline	[__]	[__]
Class management	[__]	[__]
Tuition or other charges	[__]	[__]
Other (Please specify: ________)	[__]	[__]

B4. Generally speaking, how many parents of your students pay respect to you?

1. None 2. Just a few 3. About a half 4. More than half 5. All of them

B5. How many students in your class have a habit of smoking?

1. None 2. A few 3. Many 4. A great number

B6. How many students in your class have a habit of drinking alcohol?

1. None 2. A few 3. Many 4. A great number

B7. How many students in your class frequently go to Internet cafes or video arcades?

1. None 2. A few 3. Many 4. A great number

B8. How much time do you spend on communicating with your students after class everyday as a homeroom teacher?

[__|__] hours and [__|__] minutes

B9. In your class, what are the numbers of students with each of the following types of Hukou? (Please fill in 000 if none.)

	**The number of students**
Hukou of the local county/district	[__|__|__]
Hukou of non-local counties/districts of this province	[__|__|__]
Hukou of other provinces	[__|__|__]

B10. Do you think the parents of local students would support or oppose that there are non-local students in class?

1. Strongly oppose 2. Somewhat oppose 3. Neither oppose nor support
4. Somewhat support 5. Strongly support

B11. Do you think the parents of local students would be willing to communicate with the parents of non-local students?

1. Very unwilling to 2. Somewhat unwilling to 3. Moderate
4. Somewhat willing to 5. Very willing to

(Please continue to finish Part C.)

Part C: Background and Opinions

C1. Your sex is:

1. Male　　　2.Female

C2. Your year of birth is: [__|__|__|__]

C3. What is your current marital status?

1. Not married　　　2. Married　　　3. Widowed
4. Divorced/separated　　　5. Other

C4. Up till now, what is the highest educational diploma you have attained?

1. Junior high school degree or below
2. Vocational high school/technical secondary school/technical school degree
3. Senior high school degree
4. Junior college degree
5. Bachelor degree (attained through adult higher education)
6. Bachelor degree (attained through regular higher education)
7. Master degree or higher

C5. Are you a graduate from pedagogical universities or pedagogical majors?

1. Yes.　　　2. No.

C6. Do you have a teacher certification?

1. Yes.　　　2. No.

C7. Have you ever received psychological health training?

1. Yes, and I have license A.　　　2. Yes, and I have license B.　　　3. No.

C8. How many years of experience do you have in teaching?

[__|__] year(s)

C9. Have you ever taught in other schools before you came to this school?

1. No　　　2. Yes

C10. When did you begin to teach in this school?

[__|__|__|__][__|__] ([YYYY] [MM])

C11. Are you an institutionally registered teacher admitted by the government?

1. Yes, I became a registered teacher in the year [__|__|__|__].
2. No, I'm not.

C12. Do you have a professional title in teaching? If yes, what is it?

0. No, I don't.
1. Level-C Teacher　　　2. Level-B Teacher　　　3. Level-A Teacher
4. Senior-Level Teacher　　　5. Principal Senior-Level Teacher

C13. Are you holding other concurrent administrative posts? If yes, what is it?

0. No, I’m not
1. Head of the grade
2. Head of the teaching & research group of a subject
3. Teaching director or deputy teaching director
4. Deputy principal
5. Principal

C14. Do you receive government subsidies?

1. Yes 2. No

C15. When did you first take the post of a homeroom teacher?

[__|__|__|__][__|__] ([YYYY] [MM])

C16. Which of the following grades have you ever taught? (Please mark all that apply.)

1. The seventh grade 2. The eighth grade 3. The ninth grade
4. Elementary school grades 5. Senior high school grades

C17. Which of the following awards have you ever won because of your teaching performance? (Please mark all that apply.)

1. National award 2. Province-level award 3. City-level award
4. County-level award 5. School-level award 6. None

C18. Have you ever won the title of Excellent Homeroom Teacher over the past three years?

1. Yes, more than once 2. Yes, I got the title once
3. No 4. There is no such title in this school’s evaluation system

C19. In general, are you satisfied with your current job as a homeroom teacher?

1. Strongly unsatisfied 2. Somewhat unsatisfied 3. Moderate
4. Somewhat satisfied 5. Strongly satisfied

C20. To what extent are you willing to take the post again as a homeroom teacher after you finished teaching this class?

1. Strongly unwilling to 2. Somewhat unwilling to 3. I don’t care
4. Somewhat willing to 5. Strongly willing to

C21. How many close friends do you have among your colleagues in this school?

1. None 2. Just a few 3. Some 4. Many

C22. Have you ever feel tired of being a teacher?

1. Never 2. Seldom 3. Sometimes 4. Often

C23. Are you still willing to choose teacher as your job if you have a second chance to make a choice?

1. No 2. Not sure 3. Definitely yes

C24. Are you still willing to become a junior-high teacher if you have a second chance to make a choice?

1. No 2. Not sure 3. Definitely yes

C25. Are you still willing to teach in this school if you have a second chance to choose an institution to work for?

1. No 2. Not sure 3. Definitely yes

C26. Are you satisfied with each of the following aspects of this school?

	Strongly unsatisfied	Somewhat unsatisfied	Moderate	Somewhat satisfied	Strongly satisfied
Salary	1	2	3	4	5
School management	1	2	3	4	5
Facility	1	2	3	4	5
Students' quality	1	2	3	4	5

C27. What do you think of the school's academic atmosphere?

1. Very bad 2. Bad 3. Moderate 4. Good 5. Very good

C28. What do you think of the school's management of students?

1. Very loose 2.Loose 3. Moderate 4.Strict 5. Very strict

C29. In your opinion, what are the capabilities or qualities of students that the school needs to improve its educating further? (Please mark all that apply.)

1. The capability of analyzing and solving problems
2. Creativity and imagination
3. Self-discipline and perseverance
4. Positive values and morality
5. Interpersonal relationship, communication and cooperation
6. Mental health
7. Concerns for and engagement in public affairs

C30. In what aspects do you think the school should bear more responsibility than students' family? (Please mark all that apply.)

1. The capability of analyzing and solving problems
2. Creativity and imagination
3. Self-discipline and perseverance
4. Positive values and morality
5. Interpersonal relationship, communication and cooperation
6. Mental health
7. Concerns for and engagement in public affairs

C31. What do you think is the most important goal of school education? (Please mark all that apply.)

1. To foster virtue

2. To pass on basic knowledge
3. To foster comprehensive capabilities
4. To increase senior high school/college/university acceptance rate
5. To make sure students can grow up happily and safely

Part D: Contact Information

We hope to continue the tracking of the students' future progress through this panel survey. Please leave your contact information so that we could keep in touch with you and your students:

D1. Your office phone number:

D1a. Area code: [__| __ | __ | __]

D1b. Phone number: [__| __ | __ | __ | __ | __ | __ | __]

D2. Your mobile phone number: [__| __ | __ | __ | __ | __ | __ | __ | __ | __ | __]

D3. Your Email address: ___________________

D4. Your QQ number: [__| __ | __ | __ | __ | __ | __ | __ | __ | __]

This is the end of the questionnaire. Thank you for your engagement and cooperation! We wish you a good health and successful career!

Class: ________

Name of Homeroom Teacher: ____________

Name of School: ____________________

Location of School: County/District: ___________ City: ___________

Province (Autonomous Region/Municipality): ___________

China Education Panel Survey

Academic Year 2014-2015

Homeroom Teacher Questionnaire

Teachers answering this questionnaire do not need to fill in the blanks in the box below.

1. Code of Questionnaire: [____|____|____|____|____|____]

2. Code of County/District: [____|____|____|____|____|____]

Distinguished teachers,

Hello!

China Education Panel Survey (CEPS) is the first large-scale, nationwide, and longitudinal social survey of junior high students in China. It is jointly conducted by the National Survey Research Center (NSRC) at Renmin University of China and academic institutions throughout the country. During the academic year of 2013-2014, we surveyed nearly 20,000 students as well as their parents, homeroom teachers, subject teachers and school administrators. Through scientific sampling, your class has been chosen as the sample class. This year, we hope this follow-up survey would help us understand the growth and progress of your students. We would appreciate it very much if you could help us finish this questionnaire. Thank you for your understanding and support.

There are neither right nor wrong answers to these questions and your answers will not be evaluated as part of your work performance or that of your school. Therefore, please write down your honest ideas and the reality of the sample class. We pledge here that under *the Statistics Law of the People's Republic of China*, we will hold all the information you are going to provide in strict confidence and it will never be given away to any individual or institution. Thank you for your cooperation.

National Survey Research Center (NSRC), Renmin University of China

March, 2015

[Answering Instruction]

When answering a multiple-choice question, please circle the number of your choice; when answering a blank-filling question, please write down words or numbers on the lines in the square brackets.

Part A: Teaching

A1. What subject(s) do you teach as the homeroom teacher of this class? (Please mark all that apply.)

1. Math　2. Chinese　3. English　4. Physics
5. Chemistry　6. Biology　7. History　8. Geography
9. Politics　10. Other (Please specify: __________)

A2. When did you take the post of homeroom teacher of this class?

In the year of [__|__|__|__]

A3. How was the academic performance of your class compared with other classes of the same grade *WHEN YOU TOOK THE POST* of homeroom teacher of *THIS CLASS*?

1. Near the bottom　2. Below average　3. Average
4. Above average　5. Among the best

A4. *AT PRESENT,* how is the academic performance of your class compared with other classes of the same grade?

1. Near the bottom　2. Below average　3. Average
4. Above average　5. Among the best

A5. *AT PRESENT,* is the grade to which the sample class belongs divided according to the total score or the score of a single subject?

1. Yes　2. No

A6. How many *OTHER CLASSES* do you teach besides the sample class?

[__|__] class(s)

A7. How many class hours did you teach *LAST WEEK*?

[__|__] hour(s)

A8. How many hours did you spend on preparing lessons *LAST WEEK*?

[__|__] hour(s) (Please fill in 00 if you don't need to prepare lessons.)

A9. How many hours did you spend on correcting homework and/or test papers *LAST WEEK*?

[__|__] hour(s) (Please fill in 00 if you don't need to correct homework or test papers.)

A10. How long was your total working hours *LAST WEEK*?

[___|___|___] hour(s) (including teaching, preparing lessons, correcting homework and/or test papers, and home visiting.)

A11. Do you often discuss the subject you teach with your colleagues *SINCE THE BEGINNING OF THIS SEMESTER*?

1. Yes, I do.　2. No, I don't.

A12. What's the most discussed teaching-related topic between you and your colleagues? (Please mark all that apply.)

1. Content of textbooks
2. Teaching methods
3. Exam paper design
4. Students' tutoring

A13. How often do you apply each of the following teaching methods to the sample class?

	Never	Seldom	Sometimes	Often	Always
Lectures	1	2	3	4	5
Group discussion	1	2	3	4	5
Interaction between the teacher and students	1	2	3	4	5

A14. How often do you adopt each of the following facilities in your teaching?

	Never	Seldom	Sometimes	Often	Always
Multimedia projector	1	2	3	4	5
Internet	1	2	3	4	5
Wall maps, models, posters	1	2	3	4	5
Personal teaching website, blog or microblog	1	2	3	4	5

A15. What's the proportion of each of the following teaching materials used in your teaching?

	Percentage
Domestic textbooks	[__|__|__]%
Foreign textbooks	[__|__|__]%
Reference materials	[__|__|__]%
Teaching materials designed by yourself	[__|__|__]%
Total	100%

A16. How much pressure do you have concerning the following aspects?

	None	Low	Moderate	High	Very high
Students' poor academic records	1	2	3	4	5
Students' uneven academic records	1	2	3	4	5
School's evaluation of teachers	1	2	3	4	5
School's administrative measures	1	2	3	4	5

	None	Low	Moderate	High	Very high
Requirements of students' parents	1	2	3	4	5
Students' enrollment rate	1	2	3	4	5
Public opinions about teachers	1	2	3	4	5
Publication of academic papers	1	2	3	4	5

A17. Take the main subject you teach as an example, how great is the influence of each of the following elements on students' academic record?

	Almost no influence	Some influence	Very great influence
Students' talent	1	2	3
Students' attitude to learning	1	2	3
Students' method of learning	1	2	3
Students' family background	1	2	3
Students' circle of friends	1	2	3
Teachers' teaching method	1	2	3
The amount of attention paid to students by the teacher	1	2	3
Teachers' salary	1	2	3
The school's management	1	2	3
The school's teaching facilities	1	2	3

A18. Generally speaking, how many students in this class pay respect to you?

1. Very few 2. Less than half 3. Half of the class
4. More than half 5. Almost everyone

A19. What kind of teaching method do you think best suits the following types of classes?

	Lectures	Group discussion	Interaction between the teacher and students
Classes composed of only local students	1	2	3
Classes in which the majority are local students and only a small number are from non-local counties/districts	1	2	3
Classes in which more than one third are from non-local counties/districts	1	2	3

A20. What's your opinion of the following aspects of each type of classes?

	**Effect of teaching:**<br>1. Very bad<br>2. Bad<br>3. Moderate<br>4. Good<br>5. Very good	**Class discipline:**<br>1. Very bad<br>2. Bad<br>3. Moderate<br>4. Good<br>5. Very good	**Relations among students:**<br>1. Very bad<br>2. Bad<br>3. Moderate<br>4. Good<br>5. Very good
Classes composed of only local students	[___]	[___]	[___]
Classes in which the majority are local students and only a small number are from non-local counties/districts	[___]	[___]	[___]
Classes in which more than one third are from non-local counties/districts	[___]	[___]	[___]

A21. Which type of class would you like to be in charge of if you can make a choice?

1. Classes composed of only local students
2. Classes in which the majority are local students and only a small number are from non-local counties/districts
3. Classes in which more than one third are from non-local counties/districts
4. I don’t care

A22. Assuming that a student named Xiaoming in Grade 8 cannot stay focused in class or sit still, and is always playing while studying, overactive and impulsive, what would you do if you met a student like him? (Please mark all that apply)

1. I would be unconcerned and isolate Xiaoming
2. I would criticize Xiaoming in public
3. I would criticize Xiaoming in private
4. I would try to help Xiaoming
5. I would ask Xiaoming’s parents to help him together
6. I would tell other students not to isolate Xiaoming
7. I would treat it as a normal phenomenon and do nothing

A23. Assuming that a student named Xiaohong in Grade 8 recently become depressed, passionless, and slow in thinking and acting, what would you do if you met a student like her? (Please mark all that apply)

1. I would be unconcerned and isolate Xiaohong
2. I would criticize Xiaohong in public
3. I would criticize Xiaohong in private
4. I would try to help Xiaohong
5. I would ask Xiaohong’s parents to help her together
6. I would tell other students not to isolate Xiaohong
7. I would treat it as a normal phenomenon and do nothing

(Please continue to finish Part B.)

Part B: Class Management

B1. How many parents of your students do you know about? (Here "know about" means that you have met and talked with them, and know their basic situation.)

1. None 2. Just a few 3. About a half 4. More than half 5. All of them

B2. How do you usually contact the parents of your students? (Please mark all that apply)

1. Home visits 2. Phone calls 3.Text messages 4. E-mails
5. QQ 6. Other 7. There's no need to contact parents

B3. How often have you had conflicts with the parents of your students *SINCE THE BEGINNING OF THIS SEMESTER*? (Such as quarrelling with the parents due to issues concerning students' academic record, class management, etc. or being reported to the school administrators by the parents.)

	**The number of parents you have had conflicts with:**<br><br>1. None<br>2. One to four<br>3. Five or above	**The major means you have adopted to settle these conflicts:**<br>1. Your negotiation with the parents<br>2. The school's negotiation with the parents<br>3. Higher educational departments' negotiation with the parents<br>4. Other
Students' academic performance	[__]	[__]
Students' discipline	[__]	[__]
Class management	[__]	[__]
Tuition or other charges	[__]	[__]
Other (Please specify: ________)	[__]	[__]

B4. Generally speaking, how many parents of your students pay respect to you?

1. None 2. Just a few 3. About a half 4. More than half 5. All of them

B5. How many students in your class have a habit of smoking?

1. None 2. A few 3. Many 4. A great number

B6. How many students in your class have a habit of drinking alcohol?

1. None 2. A few 3. Many 4. A great number

B7. How many students in your class frequently go to Internet cafes or video arcades?

1. None 2. A few 3. Many 4. A great number

B8. How much time do you spend on communicating with your students after class everyday as a homeroom teacher?

[__|__] hours and [__|__] minutes

B9. In your class, what are the numbers of students with each of the following types of Hukou? (Please fill in 000 if none.)

	The number of students
Hukou of the local county/district	[__|__|__]
Hukou of non-local counties/districts of this province	[__|__|__]
Hukou of other provinces	[__|__|__]

B10. Do you think the parents of local students would support or oppose that there are non-local students in class?

1. Strongly oppose 2. Somewhat oppose 3. Neither oppose nor support
4. Somewhat support 5. Strongly support

B11. Do you think the parents of local students would be willing to communicate with the parents of non-local students?

1. Very unwilling to 2. Somewhat unwilling to 3. Moderate
4. Somewhat willing to 5. Very willing to

(Please continue to finish Part C.)

Part C: Background and Opinions

C1. Your sex is:

1. Male 2.Female

C2. Your year of birth is: [__|__|__|__]

C3. What is your current marital status?

1. Not married 2. Married 3. Widowed
4. Divorced/separated 5. Other

C4. Up till now, what is the highest educational diploma you have attained?

1. Junior high school degree or below
2. Vocational high school/technical secondary school/technical school degree
3. Senior high school degree
4. Junior college degree
5. Bachelor degree (attained through adult higher education)
6. Bachelor degree (attained through regular higher education)
7. Master degree or higher

C5. Are you a graduate from pedagogical universities or pedagogical majors?

1. Yes. 2. No.

C6. Do you have a teacher certification?

1. Yes. 2. No.

C7. Have you ever received psychological health training?

1. Yes, and I have license A. 2. Yes, and I have license B. 3. No.

C8. How many years of experience do you have in teaching?

[__|__] year(s)

C9. Have you ever taught in other schools before you came to this school?

1. No 2. Yes

C10. When did you begin to teach in this school?

[__|__|__|__][__|__] ([YYYY] [MM])

C11. Are you an institutionally registered teacher admitted by the government?

1. Yes, I became a registered teacher in the year [__|__|__|__].
2. No, I'm not.

C12. Do you have a professional title in teaching? If yes, what is it?

0. No, I don't.
1. Level-C Teacher 2. Level-B Teacher 3. Level-A Teacher
4. Senior-Level Teacher 5. Principal Senior-Level Teacher

C13. Are you holding other concurrent administrative posts? If yes, what is it?

0. No, I’m not
1. Head of the grade
2. Head of the teaching & research group of a subject
3. Teaching director or deputy teaching director
4. Deputy principal
5. Principal

C14. Do you receive government subsidies?

1. Yes 2. No

C15. When did you first take the post of a homeroom teacher?

[__|__|__|__][__|__] ([YYYY] [MM])

C16. Which of the following grades have you ever taught? (Please mark all that apply.)

1. The seventh grade 2. The eighth grade 3. The ninth grade
4. Elementary school grades 5. Senior high school grades

C17.Which of the following awards have you ever won because of your teaching performance? (Please mark all that apply.)

1. National award 2. Province-level award 3. City-level award
4. County-level award 5. School-level award 6. None

C18. Have you ever won the title of Excellent Homeroom Teacher over the past three years?

1. Yes, more than once 2. Yes, I got the title once
3. No 4. There is no such title in this school’s evaluation system

C19. In general, are you satisfied with your current job as a homeroom teacher?

1. Strongly unsatisfied 2. Somewhat unsatisfied 3. Moderate
4. Somewhat satisfied 5. Strongly satisfied

C20. To what extent are you willing to take the post again as a homeroom teacher after you finished teaching this class?

1. Strongly unwilling to 2. Somewhat unwilling to 3. I don’t care
4. Somewhat willing to 5. Strongly willing to

C21. How many close friends do you have among your colleagues in this school?

1. None 2. Just a few 3. Some 4. Many

C22. Have you ever feel tired of being a teacher?

1. Never 2. Seldom 3. Sometimes 4. Often

C23. Are you still willing to choose teacher as your job if you have a second chance to make a choice?

1. No 2. Not sure 3. Definitely yes

C24. Are you still willing to become a junior-high teacher if you have a second chance to make a choice?

1. No 2. Not sure 3. Definitely yes

C25. Are you still willing to teach in this school if you have a second chance to choose an institution to work for?

1. No 2. Not sure 3. Definitely yes

C26. Are you satisfied with each of the following aspects of this school?

	Strongly unsatisfied	Somewhat unsatisfied	Moderate	Somewhat satisfied	Strongly satisfied
Salary	1	2	3	4	5
School management	1	2	3	4	5
Facility	1	2	3	4	5
Students' quality	1	2	3	4	5

C27. What do you think of the school's academic atmosphere?

1. Very bad 2. Bad 3. Moderate 4. Good 5. Very good

C28. What do you think of the school's management of students?

1. Very loose 2.Loose 3. Moderate 4.Strict 5. Very strict

C29. In your opinion, what are the capabilities or qualities of students that the school needs to improve its educating further? (Please mark all that apply.)

1. The capability of analyzing and solving problems
2. Creativity and imagination
3. Self-discipline and perseverance
4. Positive values and morality
5. Interpersonal relationship, communication and cooperation
6. Mental health
7. Concerns for and engagement in public affairs

C30. In what aspects do you think the school should bear more responsibility than students' family? (Please mark all that apply.)

1. The capability of analyzing and solving problems
2. Creativity and imagination
3. Self-discipline and perseverance
4. Positive values and morality
5. Interpersonal relationship, communication and cooperation
6. Mental health
7. Concerns for and engagement in public affairs

C31. What do you think is the most important goal of school education? (Please mark all that apply.)

1. To foster virtue

2. To pass on basic knowledge
3. To foster comprehensive capabilities
4. To increase senior high school/college/university acceptance rate
5. To make sure students can grow up happily and safely

Part D: Contact Information

We hope to continue the tracking of the students' future progress through this panel survey. Please leave your contact information so that we could keep in touch with you and your students:

D1. Your office phone number:

D1a. Area code: [__| __ | __ | __]

D1b. Phone number: [__| __ | __ | __ | __ | __ | __ | __]

D2. Your mobile phone number: [__| __ | __ | __ | __ | __ | __ | __ | __ | __ | __]

D3. Your Email address: ___________________

D4. Your QQ number: [__| __ | __ | __ | __ | __ | __ | __ | __ | __]

This is the end of the questionnaire. Thank you for your engagement and cooperation! We wish you a good health and successful career!

China Education Panel Survey
Academic Year 2014-2015
School Administrator Questionnaire

Province (Autonomous Region/Municipality):_______________

County (District): _____________ City: _______________

Name of School: ____

- -

School administrators answering this questionnaire do not need to fill in the blanks in the box below.

Code of Questionnaire: [____|____|____|____|____|____]

Code of County/District: [____|____|____|____|____|____]

Distinguished school administrators,

China Education Panel Survey (CEPS) is the first large-scale, nationwide, and longitudinal social survey of junior high students in China. It is jointly conducted by the National Survey Research Center (NSRC) at Renmin University of China and academic institutions throughout the country. During the academic year of 2013-2014, we surveyed nearly 20,000 students as well as their parents, homeroom teachers, subject teachers and school administrators. Through scientific sampling, your school has been chosen as the sample school. This year, we hope this follow-up survey would help us understand the growth and progress of your school and students. We would appreciate it very much if you could help us finish this questionnaire. Thank you for your understanding and support.

There are neither right nor wrong answers to these questions and your answers will not be evaluated as part of your work performance or that of your school. Therefore, please answer all the questions in this questionnaire truthfully based on your own ideas and the reality of the school. We pledge here that under *the Statistics Law of the People's Republic of China*, we will hold all the information you are going to provide in strict confidence and it will never be given away to any individual or institution. Thank you for your cooperation.

National Survey Research Center (NSRC), Renmin University of China

March, 2015

[Answering Instruction]

When answering a multiple-choice question, please circle the number of your choice; when answering a blank-filling question, please write down words or numbers on the lines in the square brackets.

Part A: Basic Information of the School

A1. Which category best describes your school:

1. Public school
2. Private school subsidized by the government
3. Ordinary private school
4. Private school for children of migrant workers →
5. Other (Please specify: __________)

Have you acquired the license from the government to run the school?
1. Yes, we got it in the year [__|__|__|__]
2. Not yet, but we have got governmental support.
3. Not yet, and we are not supported by the government

A2. Does the junior high department of your school enroll students from non-local counties / districts?

1. Yes 2. No

A3. Judging from the conditions of running schools, what is the current ranking of the junior high department of your school in the local county/district?

1. Near the bottom
2. Below average
3. Average
4. Above average
5. Among the best

A4. *IN THE PAST YEAR*, has your school been moved, expanded or reconstructed?

1. Yes 2. No

A5. *IN THE PAST YEAR*, has your school been merged with other schools?

1. Yes 2. No

A6. Does your school have a playground with circular lanes?

1. Yes, we have lanes of [__|__|__|__] meters 2. No

A7. Does your school offer students' dormitories?

1. Yes, all the students live in school
2. Yes, some students live in school
3. No

A8. How many dormitory rooms are there in your school? (Please fill in 0000 if the school does not provide dormitories.)

[__|__|__|__] rooms; [__|__] persons/room on average

A9. Does your school have the following facilities:

	No	Yes, but need to be improved	Yes, and well equipped
Laboratory	1	2	3
Computer room	1	2	3
Library	1	2	3
Music room	1	2	3
Student activity room	1	2	3
Psychological counseling room	1	2	3
Student cafeteria	1	2	3
Playground	1	2	3
Gymnasium	1	2	3
Swimming pool	1	2	3

A10. How many classrooms are there in your school at present?

[____|____|____] classrooms in total

A11. How many seats on average are there in each classroom?

[____|____|____] seats

A12. Has your school realized *the Class Access to ICTs*? (*the Class Access to ICTs* means that electronic teaching facilities, such as computers, Internet and projectors, have been installed in the classrooms.)

1. None of the classrooms has 2.Some classrooms have
3. Most classrooms have 4.All the classrooms have

A13. How many computers does your school provide for students to use?

[___|___|___|___] (Please fill in 0000 if no computer is provided.)

A14. Do you have school's library/reading room?

1. No 2. Yes, and there are [__ | __ | __ | __] volumes in this library/reading room.

A15. How much fiscal appropriation per student has your school received *THIS YEAR*? (including funding from both central and local government. Please fill in 0000 if the school does not receive any funding from the government.)

[____|____|____|____] yuan/student

A16. Are students from non-local counties/districts in your junior high department covered by the fiscal allocation as well?

1. Yes, and the quota is the same as that of the local students
2. Yes, but the quota is less than that of the local students
3. No
4. We do not enroll students from non-local counties/districts

A17. What is the percentage of each of the following items in your school's total funding sources?

	**Percentage**
Central or provincial-level fiscal appropriations	[_____|____|____]%
Prefecture-level fiscal appropriations	[_____|____|____]%
County/district-level fiscal appropriations	[_____|____|____]%
Tuition and other charges from students	[_____|____|____]%
Operational income like renting and contracting	[_____|____|____]%
Public donations	[_____|____|____]%
Other	[_____|____|____]%

A18. At present, is your school a member of an educational group?

1. Yes, the name of the group: [________]; the year of joining the group: [__|__|__|__]

2. No

A19. Which of the following best describes the community where the school is located:

1. Center of the city/town
2. Outskirts of the city/town
3. Rural-urban fringe zone of the city/town
4. Towns outside of the city/town
5. Rural areas

A20. What is the major type of communities surrounding your school?

1. Old urban areas without reconstruction (neighboring community)
2. Single or mixed unitary community
3. Affordable housing community
4. Ordinary commodity housing community
5. Exclusive residential district
6. "Urban village" (a village transformed into or merged with a community)
7. Rural areas
8. Other (Please specify: __________)

A21. Are there any of the following sites close to your school? (Please mark all that apply.)

1. Residential area
2. Cybercafé
3. Library
4. Canteen
5. Snack stands on the street
6. Public transport stations
7. Video arcades

A22. Does juvenile delinquency happen in the community where your school is located?

1. No 2. Seldom 3. Often 4.Always

(Please continue to finish Part B.)

Part B: Basic Information of JUNIOR HIGH Students at School:

B1. The number of classes and students of each grade in your junior high school is:

	Number of classes	Total number of students
Grade 7	[__|__]	[__|__|__|__]
Grade 8	[__|__]	[__|__|__|__]
Grade 9	[__|__]	[__|__|__|__]

B2. For students in *GRADE 8*, what is the percentage of each of the following ? (Please fill in 0 if you don't have that kind of student.)

	The percentage of students
Hukou of the local county/district	[__|__|__]%
Hukou of non-local counties/districts of this province	[__|__|__]%
Hukou of other provinces	[__|__|__]%
Total	100%

B3. What do you think of the quality of students enrolled this year compared to last year?

1. Worse than last year
2. Almost the same
3. Better than last year

B4. Compared to students from the local county/district in the junior high department of your school, how do students from non-local counties/districts perform in the following subjects when entering school?

	Much poorer	Somewhat poorer	About the same	Somewhat better	Much better	No students in this school are from non-local counties/districts
Math	1	2	3	4	5	9
Chinese	1	2	3	4	5	9
English	1	2	3	4	5	9

B5. On average, what is the educational level of the students' parents in the junior high department of your school compared to other schools in the local county/district?

1. Near the bottom   2. Below average   3. Average
4. Above average   5. Among the highest

B6. On average, what is the level of income of the students' parents in the junior high department of your school in the local county/district?

1. Near the bottom   2. Below average   3. Average
4. Above average   5. Among the highest

B7. What are the top 3 professions of the students' parents in the junior high department of your school? Please fill in the following blanks with numbers above:

Top 1st: [__]   Top 2nd: [__]   Top 3rd: [__]

1. Government official/cadre
2. Cadre/Official/Administrator of public institutions, enterprises/corporations
3. Scientist, engineer, university professor or other professionals
4. Doctor, lawyer, high school/primary school teacher
5. Accountant, nurse, computer programmer or other technical staff
6. Ordinary staff or worker
7. Ordinary staff or worker in business or service
8. Worker
9. Farmer, herdsman, fisherman
10. Elementary worker (for example: cleaner, guard, housekeeper, or sanitation worker)

B8. What are the top 3 professions of the *NON-LOCAL* students' parents in the junior high department of your school? (Please fill in the following blanks with numbers in Question B7. If no students in this school are from non-local counties/districts, please fill in "99" in each of the following blank.)

Top 1st: [__]   Top 2nd: [__]   Top 3rd: [__]

B9. What are the differences between parents from the local county/district and non-local counties/districts in the following situations?

	Those from the local county/district do better than those from non-local counties/districts	About the same	Those from the local county/district do worse than those from non-local counties/districts	No students in this school are from non-local counties/districts
Concern about child's academic record	1	2	3	9
Concern about child's daily life	1	2	3	9
Expectation on child's education	1	2	3	9
Management of child's behaviors and habits	1	2	3	9
Communication with school teachers	1	2	3	9
Cooperation with the school	1	2	3	9

B10. *IN THE LAST ACADEMIC YEAR* (2013-2014), how many students in your school have dropped out, transferred out of or into the school? (Please fill in 0 if no such student.)

	Drop out	Transfer out	Transfer in
Grade 7	[__|__|__]	[__|__|__]	[__|__|__]
Grade 8	[__|__|__]	[__|__|__]	[__|__|__]
Grade 9	[__|__|__]	[__|__|__]	[__|__|__]

B11. Does your school worry about the following situations that will possibly happen *IN FIVE YEARS*?

	Very much worried	Somewhat worried	Not very worried	Not worried at all
Increasing number of students	1	2	3	4
Difficulty to enroll students	1	2	3	4
Student loss	1	2	3	4
Teacher loss	1	2	3	4
Dismantlement and merging of the school	1	2	3	4

(Please continue to finish Part C.)

Part C: Basic Information of Teachers in the JUNIOR HIGH Department

C1. What is the number of teachers in your junior high department in each of the following categories? (Please fill in 0 if there is no teacher in your school fit a particular category.)

	**Number of teachers**
Total number of teachers in junior high department	[____|____|____]
Among them: the number of male teachers	[____|____|____]
The number of teachers in junior high department who have a Teacher Certification	[____|____|____]
The number of substitute teachers in junior high department	[____|____|____]
The number of teachers in junior high department resigned or retired in the academic year 2013-2014	[____|____|____]
The number of newly hired teachers in junior high department in the academic year 2013-2014	[____|____|____]
The ***STUDENTS TO TEACHERS RATIO*** at present	[____]:[____]

C2. Is it easy or difficult for your junior high department to hire new teachers in each of the following subjects?

	**Somewhat easy**	**Somewhat difficult**	**Very difficult**
Math	1	2	3
Chinese	1	2	3
English	1	2	3

C3. What is the number of teachers in your junior high department with different number of years' teaching experience? (Please fill in 000 if no teacher in your school fit a particular category.)

	**Number of teachers**
0~4 years of teaching experience	[____|____|____]
5~9 years of teaching experience	[____|____|____]
Over 10 years of teaching experience	[____|____|____]

C4. What is the number of teachers in your junior high department having the following educational background? (Please fill in 0 if no teacher in your school fit a particular category.)

	Total number of teachers	Number of teachers graduated from normal colleges or pedagogical majors:
Junior high school degree or below	[____|____|____]	(none)
Senior high school/technical secondary school/vocational high school degree	[____|____|____]	(none)
Junior college degree	[____|____|____]	[____|____|____]
Bachelor degree	[____|____|____]	[____|____|____]
Master degree	[____|____|____]	[____|____|____]

C5. What is the number of teachers in your junior high department having the following professional titles? (Please fill in 0 if no teacher in your school fit a particular category.)

	Number of teachers
Not rated	[____|____|____]
Level-C teacher	[____|____|____]
Level-B teacher	[____|____|____]
Level-A teacher	[____|____|____]
Senior-level teacher	[____|____|____]
Principal senior-level teacher	[____|____|____]

C6. What is the annual average income of the following types of teachers in your school?

	Annual average income
Senior-level teachers	[____|____|____|____|____|____] *yuan*
Teachers with a teaching experience of over 10 years	[____|____|____|____|____|____] *yuan*

C7. At present, does your junior high department hire teachers from all over the country?

1. Yes 2. No

C8. What is the minimum standard of educational background when your junior high department hire new teachers?

1. Junior high school degree or below
2. Senior high school/technical secondary school/vocational high school degree
3. Junior college degree
4. Bachelor degree
5. Master degree

C9. Is a teacher certification required when applying for the teaching posts in your school?

1. Yes 2. No

C10. Does your junior high department offer official teachers' establishment when hiring new teachers?

1. Yes 2. No

C11. In which of the following subjects has your junior high department ever lowered the employment standards because of the shortage of teachers? (Please mark all that apply.)

1. Chinese
2. Math
3. English
4. Other (Please specify: __________)
5. No, we have never done that

C12. Does your junior high department provide training programs for teachers? (including all kinds of training programs organized by your school, the local education commission or the local education bureau)

1. Yes, we had [__|__] training courses in total last year
2. No.

C13. Does your junior high department provide psychological health training program for teachers?

1. Yes, we had [__|__] training courses in total last year
2. No.

C14. Is the salary of teachers in your junior high department subsidized by the government?

1. Yes 2. No

(Please continue to finish Part D.)

Part D: Enrollment of Students in the JUNIOR HIGH Department

D1. How much does a junior high student *FROM LOCAL COUNTY/DISTRICT* in Grade 8 have to pay for each of the following items *LAST SEMESTER*? (Please fill in 0 if there is no charge for a particular item.)

	**Students from the local county/district**
Tuition fees	[___|___|___|___|___] Yuan
Book fees	[___|___|___|___|___] Yuan
Supplementary education material fees	[___|___|___|___|___] Yuan
Uniform fees	[___|___|___|___|___] Yuan
Activity fees	[___|___|___|___|___] Yuan
Insurance fees	[___|___|___|___|___] Yuan
Meals charge	[___|___|___|___|___] Yuan
Boarding fees	[___|___|___|___|___] Yuan
Other (Please specify: ______)	[___|___|___|___|___] Yuan

D2. *LAST SEMESTER*, which of the following do your junior high department charge differently in grade 8 between students from the local county/district and students from non-local counties/districts? (Please mark all that apply)

0. All fees are the same
1. Tuition fees
2. Book fees
3. Supplementary education material fees
4. Uniform fees
5. Activity fees
6. Insurance fees
7. Meals charge
8. Boarding fees
9. Other (Please specify: _______)

D3. *LAST SEMESTER*, are students in your junior high department from the *LOCAL COUNTY/DISTRICT* offered the following governmental subsidies in education? (Please mark all that apply.)

1. Exemption of book fees
2. Free breakfast/lunch offered by the school
3. Grants for poor students
4. Other (Please specify: __________)
5. No subsidies
6. No students in junior high department are from the local county/district

D4. *LAST SEMESTER*, are students in your junior high department from the *NON-LOCAL* county/district offered the following governmental subsidies in education? (Please mark all that apply.)

1. Exemption of book fees
2. Free breakfast/lunch offered by the school
3. Grants for poor students
4. Other (Please specify: __________)
5. No subsidies
6. No students in junior high department are from the non-local county/district

(Please continue to finish Part E.)

Part E: Management of the JUNIOR HIGH Department

E1. Generally speaking, how many days are students in Grade 8 required to attend school every week in your school? (1 decimal digit is reserved.)
[__].[__]days

E2. At what time are students in Grade 8 required to arrive and leave school every day? (24-hour time system)
Time required to arrive at school: [__|__]:[__|__]
Time required to leave school: [__|__]:[__|__]

E3. How many classes are students in Grade 8 required to take every day and how long does each class last?
Number of classes everyday: [__|__]
Lasting period of each class: [__|__]

E4. Does your school offer classrooms for students in Grade 8 to self-study at night?
1. Yes 2. No **(SKIP TO E8)**

E5. Does your school require students in Grade 8 to self-study at night in school?
1. Yes 2. No **(SKIP TO E8)**

E6. The time arrangement of self-studying at night for students in Grade 8: (24-hour time system)
Starting time: [__|__]: [__|__]
Ending time: [__|__]: [__|__]

E7. Will there be teachers in classroom to monitor students' self-study for Grade 8 at night in school?
1. Yes 2. No

E8. Does your school organize the following activities for students in Grade 8 in the summer or winter vacation? (Please mark all that apply.)
0. No
1. Cram classes
2. Advanced classes
3. Summer/winter camps
4. Other (Please specify: __________)

E9. How many students in Grade 8 can you name in your school? (Please fill in the percentage)
[__|__|__]%

E10. Does Grade 8 in your school fit in each of the following descriptions?

	Not fit at all	Somewhat not fit	Somewhat fit	Exactly fit
Parents do not cooperate with the school.	1	2	3	4
Parents demand their children to be in specific classes or be taught by specific teachers.	1	2	3	4
Students often skip school.	1	2	3	4
It is hard to manage the school discipline.	1	2	3	4
The school is crowded.	1	2	3	4
The flow of teachers is frequent.	1	2	3	4

E11. How often did each of the following happen in Grade 8 *LAST WEEK*?

	Never	1-4 times	5-10 times	Over 10 times
Students' fight with each other	1	2	3	4
Students' vandalism	1	2	3	4
Students' smoking	1	2	3	4
Students' drinking	1	2	3	4
Gang activities within or outside the campus	1	2	3	4
Chaotic classroom atmosphere	1	2	3	4
Teachers scolding at students	1	2	3	4
Corporal punishment of students by teachers	1	2	3	4

E12. If students in Grade 8 *FROM OTHER PROVINCES* are transferred to another school or drop out, how will their school rolls/student files be handled?

1. Transfer them to the new school
2. Transfer them to its province-level educational departments
3. Offer them a school transfer certificate
4. Other (Please specify: __________)

E13. Does your school accept transferred-in students in Grade 8 (from other schools)?

1. Yes 2. No **(SKIP TO E16)**

E14. How does your school manage the school rolls/student files of transferred-in students in Grade 8 *FROM OTHER PROVINCES*?

1. To continue the records and files transferred from the previous school
2. To create new records and files
3. Other (Please specify: __________)

E15. What are the standards for arranging classes for transferred-in students in Grade 8? (Please mark all that apply.)

1. Grades they have got in the entrance exam
2. Location of their hukou
3. Randomly or evenly assign them into different classes
4. Other

E16. Will your school rearrange the students in Grade 8 into different classes at the beginning of this semester?
1. Yes 2. No **(SKIP TO E18)**

E17. This rearrangement of classes is based on:
1. Chinese scores
2. Math scores
3. English scores
4. Total scores
5. Not based on academic records

E18. What are the major teaching methods used by teachers for students in Grade 8 in your school? (Please mark all that apply.)
1. Lectures
2. Group discussion
3. Bilingual teaching
4. Students choosing classes in different levels according to their own capabilities
5. Other (Please specify: __________)

E19. What measure will be taken by the school if students in Grade 8 fail to pass exams? (Please mark all that apply.)
0. No particular measure
1. Requiring them to repeat a year
2. After-class tutoring
3. Other (Please specify: __________)

E20. For students in Grade 8 performing outstandingly well in a certain subject, does your school offer opportunities for further improvement?
1. Yes 2. No

E21. On average, how many classes does a major subject (math, Chinese or English) teacher (not a homeroom one) teach in Grade 8?
[___|___] class(s) in total

E22. Is it difficult for your school to arrange teachers in Grade 8 in the following subjects?

	Not difficult	Somewhat difficult	Very difficult
Math	1	2	3
Chinese	1	2	3
English	1	2	3

E23. In which grade does your junior high department provide 'health education courses'? (Please mark all that apply)

0.No 1. Grade 7 2. Grade 8 3. Grade 9

E24. Does your junior high department arrange physical examination for all students?

0.No. 1. Yes, once an academic year. 2. Yes, but not once an academic year.

E25. Assuming that a student named Xiaoming in Grade 8 cannot stay focused in class or sit still, and is always playing while studying, overactive and impulsive, what would most of the teachers in your junior high department do if they met a student like him? (Please mark all that apply)

1. They would be unconcerned and isolate Xiaoming
2. They would criticize Xiaoming in public
3. They would criticize Xiaoming in private
4. They would try to help Xiaoming
5. They would ask Xiaoming's parents to help him together
6. They would tell other students not to isolate Xiaoming
7. They would treat it as a normal phenomenon and do nothing

E26. Assuming that a student named Xiaohong in Grade 8 recently become depressed, passionless, and slow in thinking and acting, what would most of the teachers in your junior high department do if they met a student like her? (Please mark all that apply)

1. They would be unconcerned and isolate Xiaohong
2. They would criticize Xiaohong in public
3. They would criticize Xiaohong in private
4. They would try to help Xiaohong
5. They would ask Xiaohong's parents to help her together
6. They would tell other students not to isolate Xiaohong
7. They would treat it as a normal phenomenon and do nothing

E27. At present, what are the challenges that your school is facing with in management? (Please mark all that apply.)

1. Poor quality of students enrolled
2. Too many students from non-local counties/districts
3. Poor quality of teachers
4. Lack of funding
5. Other (Please specify: __________)
6. None of the above

E28. During *THE LAST SEMESTER*, how many times did your school do each of the following?

	Never	1 time	2-4 times	Over 5 times
Parent meeting	1	2	3	4
Writing reports to parents about students' performance at school	1	2	3	4

	Never	1 time	2-4 times	Over 5 times
Inviting parents to attend classes	1	2	3	4
Inviting parents to have an informal discussion with teachers	1	2	3	4
Inviting parents to watch students' shows or to take part in extracurricular activities	1	2	3	4
Holding lectures for students on daily-life guidance like emotional management and parent-child relationship	1	2	3	4

E29. In the 2013-2014 academic year, how many students have graduated from the junior high department in your school? (Please circle "9" if no students in this school are from non-local counties/districts.)

	Students from the local county/district	Students from non-local counties/districts	No students in this school are from non-local counties/districts
Total number of graduates in the junior high department	[__|__|__|__]	[__|__|__|__]	9
Number of students admitted to key senior high schools	[__|__|__|__]	[__|__|__|__]	9
Number of students admitted to regular senior high schools	[__|__|__|__]	[__|__|__|__]	9
Number of students admitted to vocational high schools/technical secondary schools /technical schools	[__|__|__|__]	[__|__|__|__]	9
Number of students entering labor market	[__|__|__|__]	[__|__|__|__]	9

(Please continue to finish Part F.)

Part F: Personal Information

F1. What is your administrative post in this school?

1. Principal
2. Vice-principal
3. Dean
4. Other (Please specify: __________)

F2. When did you begin to hold the post in this school?

In the year [__|__|__|__]

F3. Your sex is:

1. Male 2. Female

F4. Your year of birth is: [__|__|__|__]

F5. What is your political affiliation at present?

1. Member of the Communist Party
2. Member of a Democratic Party
3. The masses

F6. What is the highest education level you have completed or are working on at present?

1. Junior high school degree or below
2. Vocational high school/technical secondary school/technical school degree
3. Senior high school degree
4. Junior college degree
5. Bachelor degree (attained through adult higher education)
6. Bachelor degree (attained through regular higher education)
7. Master degree or higher

F7. Are you a graduate from normal universities or pedagogical majors?

1. Yes 2. No

F8. Do you have a teacher certification?

1. Yes, I obtained it in the year [__|__|__|__]. 2. No

F9. Before taking the administrative post in this school, have you ever worked in an administrative department of education like the Education Bureau or the Education Commission?

1. Yes, I have worked in such department for [___|___] years. 2. No.

F10. Before taking the administrative post in this school, have you ever been a teacher at school?

1. Yes, I have been a teacher for [___|___] years. 2. No.

F11. How many years have you engaged yourself in education?

[____|____] years

F12. How many years have you worked in this school?
[____|____] years

F13. How many years have you ever served as head of schools? (including head of other schools)
[____|____] years

F14. In your opinion, what are the capabilities or qualities of students that the school needs to improve its educating further? (Please mark all that apply.)
1. The capability of analyzing and solving problems
2. Creativity and imagination
3. Self-discipline and perseverance
4. Positive values and morality
5. Interpersonal relationship, communication and cooperation
6. Mental health
7. Concerns for and engagement in public affairs

F15. In what aspects do you think the school should bear more responsibility than students' family? (Please mark all that apply.)
1. The capability of analyzing and solving problems
2. Creativity and imagination
3. Self-discipline and perseverance
4. Positive values and morality
5. Interpersonal relationship, communication and cooperation
6. Mental health
7. Concerns for and engagement in public affairs

F16. What do you think is the most important goal of school education? (Please mark all that apply.)
1. To foster virtue
2. To pass on basic knowledge
3. To foster comprehensive capabilities
4. To increase senior high school/college/university acceptance rate
5. To make sure students can grow up happily and safely

(Please continue to finish Part G.)

Part G: Contact Information

We hope to continue the tracking of the students' future progress and the development of your school through this panel survey. Please leave the contact information of the school principal and that of another person in charge so that we could keep in touch with your school and students:

G1. Contact information of you

Name: ____________________________

Office phone number: _______________ Mobile phone number: ______________

E-mail: ____________________________ QQ: ______________________________

G2. Contact information of the school principal (If you are the principal, please skip to G3)

Name: ____________________________

Office phone number: _______________ Mobile phone number: ______________

E-mail: ____________________________ QQ: ______________________________

G3. Contact information of another person in charge

Name: ____________________________

Office phone number: _______________ Mobile phone number: ______________

E-mail: ____________________________ QQ: ______________________________

This is the end of the questionnaire. Thank you for your engagement and cooperation! We wish you a good health and a successful career!
